# Identification of eight genetic variants as novel determinants of dyslipidemia in Japanese by exome-wide association studies

**DOI:** 10.18632/oncotarget.17159

**Published:** 2017-04-17

**Authors:** Yoshiji Yamada, Jun Sakuma, Ichiro Takeuchi, Yoshiki Yasukochi, Kimihiko Kato, Mitsutoshi Oguri, Tetsuo Fujimaki, Hideki Horibe, Masaaki Muramatsu, Motoji Sawabe, Yoshinori Fujiwara, Yu Taniguchi, Shuichi Obuchi, Hisashi Kawai, Shoji Shinkai, Seijiro Mori, Tomio Arai, Masashi Tanaka

**Affiliations:** ^1^ Department of Human Functional Genomics, Advanced Science Research Promotion Center, Mie University, Tsu, Japan; ^2^ CREST, Japan Science and Technology Agency, Kawaguchi, Japan; ^3^ Computer Science Department, College of Information Science, University of Tsukuba, Tsukuba, Japan; ^4^ RIKEN Center for Advanced Intelligence Project, Tokyo, Japan; ^5^ Department of Computer Science, Nagoya Institute of Technology, Nagoya, Japan; ^6^ Department of Internal Medicine, Meitoh Hospital, Nagoya, Japan; ^7^ Department of Cardiology, Kasugai Municipal Hospital, Kasugai, Japan; ^8^ Department of Cardiovascular Medicine, Inabe General Hospital, Inabe, Japan; ^9^ Department of Cardiovascular Medicine, Gifu Prefectural Tajimi Hospital, Tajimi, Japan; ^10^ Department of Molecular Epidemiology, Medical Research Institute, Tokyo Medical and Dental University, Tokyo, Japan; ^11^ Section of Molecular Pathology, Graduate School of Health Care Sciences, Tokyo Medical and Dental University, Tokyo, Japan; ^12^ Research Team for Social Participation and Community Health, Tokyo Metropolitan Institute of Gerontology, Tokyo, Japan; ^13^ Research Team for Promoting Support System for Home Care, Tokyo Metropolitan Institute of Gerontology, Tokyo, Japan; ^14^ Research Team for Social Participation and Health Promotion, Tokyo Metropolitan Institute of Gerontology, Tokyo, Japan; ^15^ Center for Promotion of Clinical Investigation, Tokyo Metropolitan Geriatric Hospital, Tokyo, Japan; ^16^ Department of Pathology, Tokyo Metropolitan Geriatric Hospital, Tokyo, Japan; ^17^ Department of Clinical Laboratory, Tokyo Metropolitan Geriatric Hospital, Tokyo, Japan

**Keywords:** triglyceride, HDL-cholesterol, LDL-cholesterol, dyslipidemia, exome-wide association study

## Abstract

We have performed exome-wide association studies to identify single nucleotide polymorphisms that influence serum concentrations of triglycerides, high density lipoprotein (HDL)–cholesterol, or low density lipoprotein (LDL)–cholesterol or confer susceptibility to hypertriglyceridemia, hypo–HDL-cholesterolemia, or hyper–LDL-cholesterolemia in Japanese. Exome-wide association studies for serum triglycerides (13,414 subjects), HDL-cholesterol (14,119 subjects), LDL-cholesterol (13,577 subjects), hypertriglyceridemia (4742 cases, 8672 controls), hypo–HDL-cholesterolemia (2646 cases, 11,473 controls), and hyper–LDL-cholesterolemia (4489 cases, 9088 controls) were performed with HumanExome-12 DNA Analysis BeadChip or Infinium Exome-24 BeadChip arrays. Twenty-four, 69, or 32 loci were significantly (*P* < 1.21 × 10^−6^) associated with serum triglycerides, HDL-cholesterol, or LDL-cholesterol, respectively, with 13, 16, or 9 of these loci having previously been associated with triglyceride-, HDL-cholesterol–, or LDL-cholesterol–related traits, respectively. Two single nucleotide polymorphisms (rs10790162, rs7350481) were significantly related to both serum triglycerides and hypertriglyceridemia; three polymorphisms (rs146515657, rs147317864, rs12229654) were significantly related to both serum HDL-cholesterol and hypo–HDL-cholesterolemia; and six polymorphisms (rs2853969, rs7771335, rs2071653, rs2269704, rs2269703, rs2269702) were significantly related to both serum LDL-cholesterol and hyper–LDL-cholesterolemia. Among polymorphisms identified in the present study, two polymorphisms (rs146515657, rs147317864) may be novel determinants of hypo–HDL-cholesterolemia, and six polymorphisms (rs2853969, rs7771335, rs2071653, rs2269704, rs2269703, rs2269702) may be new determinants of hyper–LDL-cholesterolemia. In addition, 12, 61, 23, or 3 polymorphisms may be new determinants of the serum triglyceride, HDL-cholesterol, or LDL-cholesterol concentrations or of hyper–LDL-cholesterolemia, respectively.

## INTRODUCTION

Dyslipidemia, including hypertriglyceridemia, hypo–high density lipoprotein (HDL)–cholesterolemia, and hyper–low density lipoprotein (LDL)–cholesterolemia, is a multifactorial disorder that results from an interaction between genetic background and environmental factors [1, 2]. Given that dyslipidemia is an important risk factor for coronary artery disease, ischemic stroke [3], and colorectal cancer [4], its personalized prevention is an important public health goal.

Genome-wide association studies (GWASs) [5–8] and gene-centric meta-analysis [9] have implicated various genes and loci as determinants of blood lipid levels or in predisposition to dyslipidemia in European-ancestry populations. Genetic variants associated with lipid profiles have been extensively investigated, with one recent study identifying 157 such loci, including 62 variants not previously reported [10]. Recent GWASs [11, 12] or studies based on exome [13] or whole-genome [14] sequencing in European-ancestry populations also identified low-frequency or rare variants related to circulating lipid levels. Although several polymorphisms have been shown to be related to blood lipid profiles in the Japanese population [15], genetic variants—including low-frequency or rare variants—that influence circulating lipid levels or contribute to genetic susceptibility to dyslipidemia in Japanese remain to be identified definitively.

We have now performed exome-wide association studies (EWASs) to identify single nucleotide polymorphisms (SNPs)—in particular, low-frequency or rare coding variants with moderate to large effect sizes—that influence the serum concentrations of triglycerides, HDL-cholesterol, or LDL-cholesterol or confer susceptibility to dyslipidemia in Japanese. We used Illumina arrays that provide coverage of functional SNPs in entire exons including such variants.

## RESULTS

### EWASs for serum concentrations of triglycerides, HDL-cholesterol, or LDL-cholesterol

We applied linear regression analysis to examine the relation of genotypes for 41,371 SNPs to the serum triglyceride concentration in 13,414 subjects, for 41,225 SNPs to the serum HDL-cholesterol concentration in 14,119 subjects, or for 41,347 SNPs to the serum LDL-cholesterol concentration in 13,577 subjects. Manhattan plots for the EWASs are shown in Supplementary Figure 1. After Bonferroni's correction, 46, 104, or 40 SNPs were significantly (*P* < 1.21 × 10^−6^) associated with the serum concentrations of triglycerides (Table 1), HDL-cholesterol (Supplementary Table 1), or LDL-cholesterol (Table 2), respectively.

**Table 1:** The 46 SNPs significantly (*P* < 1.21 × 10^−^^6^) associated with serum triglyceride concentration in the EWAS

Gene	dbSNP	Nucleotide (amino acid) substitution^a^	Chromosome: position	MAF (%)	*P* (genotype)
*APOA5*	rs2075291	C/A (G185C)	11: 116790676	7.3	2.98 × 10^−65^
	rs7350481	C/T	11: 116715567	27.7	3.10 × 10^−38^
*APOA5*	rs2266788	T/C	11: 116789970	26.2	1.03 × 10^−35^
*BUD13*	rs10790162	G/A	11: 116768388	26.3	1.11 × 10^−35^
*ZPR1*	rs964184	C/G	11: 116778201	26.3	2.17 × 10^−35^
	rs9326246	G/C	11: 116741017	26.5	6.24 × 10^−34^
*ZPR1*	rs2075290	T/C	11: 116782580	26.7	4.87 × 10^−33^
*MTFR2*	rs143974258	G/A (R360*)	6: 136231355	3.3	2.10 × 10^−19^
*APOA4*	rs5104	T/C (N147S)	11: 116821618	35.7	3.57 × 10^−14^
*C21orf59*	rs76974938	C/T (D67N)	21: 32609946	2.4	4.74 × 10^−14^
*LPL*	rs328	C/G (S474*)	8: 19962213	12.9	8.93 × 10^−13^
	rs10096633	C/T	8: 19973410	12.7	1.25 × 10^−12^
	rs17482753	G/T	8: 19975135	12.6	1.25 × 10^−12^
	rs12678919	A/G	8: 19986711	12.6	1.36 × 10^−12^
	rs10503669	C/A	8: 19990179	12.6	1.40 × 10^−12^
*SIK3*	rs2075292	G/T	11: 116861796	42.7	1.58 × 10^−12^
*GCKR*	rs1260326	T/C (L446P)	2: 27508073	43.6	1.62 × 10^−12^
*SIK3*	rs10047462	G/T	11: 116851325	47.6	1.78 × 10^−12^
*GCKR*	rs780093	A/G	2: 27519736	43.0	8.98 × 10^−12^
	rs1260333	T/C	2: 27525757	42.9	1.11 × 10^−11^
	rs7016880	G/C	8: 20019235	12.0	2.64 × 10^−11^
*TNC*	rs138406927	C/T (A1096T)	9: 115064848	2.1	2.66 × 10^−10^
*LAIR2*	rs34429135	T/A (F115Y)	19: 54508164	2.5	1.24 × 10^−9^
*PAFAH1B2*	rs7112513	G/A	11: 117166645	45.3	2.21 × 10^−9^
*PAFAH1B2*	rs4936367	A/G (V151M)	11: 117166645	45.3	2.24 × 10^−9^
	rs12269901	G/C	11: 117103213	36.6	2.33 × 10^−9^
*C2orf16*	rs1919128	G/A (V774I)	2: 27578892	42.2	2.45 × 10^−9^
*OR4F6*	rs141569282	G/A (A117T)	15: 101806068	1.7	8.08 × 10^−9^
	rs2954038	A/C	8: 125495147	30.3	1.20 × 10^−8^
	rs2197089	C/T	8: 19968862	27.6	3.31 × 10^−8^
*C2orf16*	rs1919127	C/T (A685V)	2: 27578626	42.2	4.30 × 10^−8^
*LOC101929011*	rs1240773	G/T	11: 116649810	29.0	5.77 × 10^−8^
*LPL*	rs15285	G/A	8: 19967156	19.2	6.16× 10^−8^
*LPL*	rs13702	A/G	8: 19966981	19.2	6.17 × 10^−8^
	rs2954026	G/T	8: 125472284	33.1	8.37 × 10^−8^
*LPL*	rs326	A/G	8: 19961928	19.4	8.74 × 10^−8^
*COL6A5*	rs200982668	G/A (E2501K)	3: 130470894	1.3	8.94 × 10^−8^
	rs1264429	A/G	6: 30597324	13.4	9.04 × 10^−8^
	rs1441756	T/G	8: 20010875	19.0	1.29 × 10^−7^
	rs2083637	T/C	8: 20007664	19.0	1.53 × 10^−7^
	rs2954033	G/A	8: 125481504	33.3	1.55 × 10^−7^
*LPL*	rs301	T/C	8: 19959423	19.3	1.62× 10^−7^
*MUC17*	rs78010183	A/T (T1305S)	7: 101035329	1.8	5.96 × 10^−7^
*MARCH1*	rs61734696	G/T (Q137K)	4: 164197303	1.2	7.44 × 10^−7^
	rs4938303	T/C	11: 116714271	43.0	8.13 × 10^−7^
*MRVI1*	rs4909945	C/T (V11I)	11: 10652192	0.3	1.03× 10^−6^

The relation of genotypes of SNPs to the serum concentration of triglycerides was examined by linear regression analysis. ^a^Major allele/minor allele.

**Table 2:** The 40 SNPs significantly (*P* < 1.21 × 10^−^^6^) associated with serum LDL-cholesterol concentration in the EWAS

Gene	dbSNP	Nucleotide (amino acid) substitution^a^	Chromosome: position	MAF (%)	*P* (genotype)
*APOE*	rs7412	C/T (R176C)	19: 44908822	4.3	6.42 × 10^−51^
*APOC1*	rs445925	C/T	19: 44912383	6.6	4.19 × 10^−18^
*APOB*	rs13306206	G/A (P955S)	2: 21019859	3.2	3.34 × 10^−16^
*PCSK9*	rs151193009	C/T (R93C)	1: 55043912	1.1	3.35 × 10^−14^
*APOE*	rs769449	G/A	19: 44906745	7.7	3.58 × 10^−11^
*PSRC1*	rs599839	A/G	1: 109279544	7.9	1.51 × 10^−10^
*CELSR2*	rs629301	A/C	1: 109275684	7.8	1.96 × 10^−10^
*APOB*	rs13306194	G/A (R532W)	2: 21029662	12.1	3.05 × 10^−10^
*CELSR2*	rs12740374	G/T	1: 109274968	7.7	4.45 × 10^−10^
	rs602633	C/A	1: 109278889	7.6	4.80 × 10^−10^
*CELSR2*	rs646776	A/G	1: 109275908	7.7	4.98× 10^−10^
*ABO*	rs1053878	G/A (P156L)	9: 133256264	22.8	1.63 × 10^−9^
	rs651007	G/A	9: 133278431	27.9	1.12 × 10^−8^
	rs579459	T/C	9: 133278724	27.9	1.18 × 10^−8^
	rs635634	G/A	9: 133279427	27.8	1.28 × 10^−8^
	rs507666	G/A	9: 136149399	27.8	1.36 × 10^−8^
*MUC22*	rs117024916	A/G (T71A)	6: 31025642	9.9	7.55 × 10^−8^
*VARS*	rs11751198	G/A	6: 31785749	9.5	1.67 × 10^−7^
*CCHCR1*	rs147733073	C/G (H486Q)	6: 31145462	10.2	1.94 × 10^−7^
	rs2853969	C/T	6: 31388797	9.7	2.02 × 10^−7^
*MSH5*	rs11754464	C/T	6: 31755958	9.5	2.88 × 10^−7^
*VARS*	rs5030798	C/T (V1055I)	6: 31779733	9.5	3.60 × 10^−7^
*PRRC2A*	rs11538264	G/A (V1774M)	6: 31635412	9.5	3.71 × 10^−7^
*FAM65B*	rs150142878	C/T (R371Q)	6: 24847657	5.6	3.72 × 10^−7^
*HSPA1B*	rs6457452	C/T	6: 31827773	9.7	3.85 × 10^−7^
*LY6G6C*	rs117894946	G/C (G75A)	6: 31719250	9.5	4.04 × 10^−7^
*C6orf48*	rs11968400	C/T	6: 31836952	9.7	4.13× 10^−7^
	rs12210887	G/T	6: 31847946	9.7	4.61 × 10^−7^
*KIAA0319*	rs4576240	G/T (P142T)	6: 24596250	5.5	4.85 × 10^−7^
	rs2596574	G/A	6: 31366397	9.7	5.67 × 10^−7^
*ZSCAN31*	rs6922302	C/G (P128A)	6: 28327533	9.6	6.40 × 10^−7^
*NEU1*	rs13118	T/A	6: 31859509	9.7	6.46 × 10^−7^
*ZSCAN26*	rs76463649	A/G (N15S)	6: 28271963	9.6	6.71 × 10^−7^
*LY6G6F*	rs17200983	C/A (P34Q)	6: 31707506	9.5	6.89 × 10^−7^
	rs3129029	A/C	6: 29694666	23.0	7.50 × 10^−7^
*LY6G6F*	rs9267546	G/A	6: 31705659	9.8	9.05 × 10^−7^
*LY6G6F*	rs9267547	G/A (A107T)	6: 31707724	10.0	9.88 × 10^−7^
*TNXB*	rs140770834	C/G (L2271V)	6: 32064851	8.8	1.14 × 10^−6^
*TNXB*	rs11751545	A/C	6: 32073266	8.8	1.14 × 10^−6^
*ABCF1*	rs4148249	C/A	6: 30590413	10.1	1.17 × 10^−6^

The relation of genotypes of SNPs to the serum concentration of LDL-cholesterol was examined by linear regression analysis. ^a^Major allele/minor allele.

### EWASs for hypertriglyceridemia, hypo–HDL-cholesterolemia, or hyper–LDL-cholesterolemia

We performed the EWAS for hypertriglyceridemia with 13,414 subjects (4742 cases, 8672 controls), that for hypo–HDL-cholesterolemia with 14,119 subjects (2646 cases, 11,473 controls), and that for hyper–LDL-cholesterolemia with 13,577 subjects (4489 cases, 9088 controls). Characteristics of the subjects are shown in Table 3. In the study of hypertriglyceridemia, age, the frequency of men, body mass index (BMI), and the prevalence of smoking, hypertension, diabetes mellitus, chronic kidney disease, and hyperuricemia as well as the serum concentrations of triglycerides and LDL-cholesterol and the ratio of LDL-cholesterol to HDL-cholesterol were greater, whereas the serum concentration of HDL-cholesterol was lower, in subjects with hypertriglyceridemia than in controls. In the study of hypo–HDL-cholesterolemia, age, the frequency of men, BMI, and the prevalence of smoking, hypertension, diabetes mellitus, chronic kidney disease, and hyperuricemia as well as the serum concentration of triglycerides and the ratio of LDL-cholesterol to HDL-cholesterol were greater, whereas the serum concentration of HDL-cholesterol was lower, in subjects with hypo–HDL-cholesterolemia than in controls. In the study of hyper–LDL-cholesterolemia, BMI, the prevalence of smoking, the serum concentrations of triglycerides and LDL-cholesterol, and the ratio of LDL-cholesterol to HDL-cholesterol were greater, whereas age, the prevalence of chronic kidney disease, and the serum concentration of HDL-cholesterol were lower, in subjects with hyper–LDL-cholesterolemia than in controls.

**Table 3:** Characteristics of the 14,337 study subjects

Characteristic	Hyper triglyceridemia	Controls	*P*	Hypo–HDL-cholesterolemia	Controls	*P*	Hyper–LDL-cholesterolemia	Controls	*P*
No. of subjects	4742	8672		2646	11,473		4489	9088	
Age (years)	62.0 ± 12.9	59.8 ± 13.8	< 0.0001	65.5 ± 14.1	60.4 ± 13.4	< 0.0001	60.0 ± 12.3	61.0 ± 13.9	< 0.0001
Sex (male/female, %)	67.3/32.7	51.2/48.8	< 0.0001	77.5/22.5	52.0/48.0	< 0.0001	53.6/46.4	57.5/42.5	0.0133
BMI (kg/m^2^)	24.4 ± 3.5	22.7 ± 3.3	< 0.0001	24.5 ± 3.6	23.0 ± 3.4	< 0.0001	23.6 ± 3.4	23.1 ± 3.5	< 0.0001
Current or former smoker (%)	44.9	31.5	< 0.0001	51.3	33.7	< 0.0001	38.5	35.1	0.0002
Hypertension (%)	63.7	47.1	< 0.0001	70.0	50.3	< 0.0001	51.0	53.6	0.0057
Diabetes mellitus (%)	31.8	19.8	< 0.0001	42.9	20.3	< 0.0001	22.0	23.8	0.0224
Chronic kidney disease (%)	27.8	21.5	< 0.0001	32.5	22.1	< 0.0001	21.4	24.9	< 0.0001
Hyperuricemia (%)	27.8	12.5	< 0.0001	27.5	15.6	< 0.0001	18.7	16.9	0.0180
Serum triglycerides (mmol/L)	2.34 ± 1.23	0.98 ± 0.33	< 0.0001	1.82 ± 1.32	1.37 ± 0.91	< 0.0001	1.52 ± 0.88	1.43 ± 1.10	< 0.0001
Serum HDL-cholesterol (mmol/L)	1.31 ± 0.37	1.61 ± 0.47	< 0.0001	0.91 ± 0.20	1.62 ± 0.41	< 0.0001	1.50 ± 0.41	1.52 ± 0.48	0.0007
Serum LDL-cholesterol (mmol/L)	3.29 ± 0.94	3.04 ± 0.79	< 0.0001	3.09 ± 0.96	3.12 ± 0.83	0.2553	3.91 ± 0.77	2.70 ± 0.56	< 0.0001
LDL-cholesterol/HDL-cholesterol	2.66 ± 1.05	2.06 ± 0.88	< 0.0001	3.37 ± 1.24	2.05 ± 0.76	< 0.0001	2.85 ± 1.11	1.95 ± 0.74	< 0.0001

Quantitative data are means ± SD and were compared between subjects with hypertriglyceridemia, hypo–HDL-cholesterolemia, or hyper–LDL-cholesterolemia and corresponding controls with the unpaired Student's *t* test. Categorical data were compared between two groups with Fisher's exact test. Based on Bonferroni's correction, a *P* value of < 0.0014 (0.05/36) was considered statistically significant.

We applied Fisher's exact test to examine the relation of allele frequencies of 41,371 SNPs to hypertriglyceridemia, of 41,225 SNPs to hypo–HDL-cholesterolemia, or of 41,347 SNPs to hyper–LDL-cholesterolemia. Manhattan plots for the EWASs are shown in Supplementary Figure 2. After Bonferroni's correction, 73, 87, or 114 SNPs were significantly (*P* < 1.21 × 10^−6^) associated with hypertriglyceridemia (Supplementary Table 2), hypo–HDL-cholesterolemia (Supplementary Table 3), or hyper–LDL-cholesterolemia (Supplementary Table 4), respectively. The genotype distributions of these SNPs were in Hardy-Weinberg equilibrium (*P* > 0.001) among the corresponding controls for hypertriglyceridemia (Supplementary Table 5), hypo–HDL-cholesterolemia (Supplementary Table 6), or hyper–LDL-cholesterolemia (Supplementary Table 7).

### Multivariable logistic regression analysis of hypertriglyceridemia, hypo–HDL-cholesterolemia, or hyper–LDL-cholesterolemia

The relation of the 73, 87, or 114 SNPs identified by the EWASs for dyslipidemia to hypertriglyceridemia, hypo–HDL-cholesterolemia, and hyper–LDL-cholesterolemia, respectively, was examined further by multivariable logistic regression analysis with adjustment for age and sex. Two SNPs—rs10790162 (G/A) of *BUD13* and rs7350481 (C/T) at 11q23.3—were significantly [*P* < 1.71 × 10^−4^ (0.05/292)] associated with hypertriglyceridemia (Table 4, Supplementary Table 8); three SNPs—rs146515657 [T/C (N650S)] of *USP4*, rs147317864 [C/T (A262T)] of *TRABD2B*, and rs12229654 (T/G) at 12q24.1—were significantly [*P* < 1.44 × 10^−4^ (0.05/348)] associated with hypo–HDL-cholesterolemia (Table 4, Supplementary Table 9); and nine SNPs—rs7771335 (A/G) at 6p22.1, rs76974938 [C/T (D67N)] of *C21orf59*, rs2071653 (C/T) of *MOG*, rs2853969 (C/T) at 6p21.3, rs2269704 (C/T) of *PPP1R18*, rs2269703 (G/A) of *NRM*, rs495089 (T/C) at 6p21.3, rs2269702 (A/G) of *MDC1*, and rs1233399 (C/T) at 6p22.1—were significantly [*P* < 1.10 × 10^−4^ (0.05/456)] associated with hyper–LDL-cholesterolemia (Table 4, Supplementary Table 10).

**Table 4:** Relation of SNPs to hypertriglyceridemia, hypo–HDL-cholesterolemia, or hyper–LDL-cholesterolemia as determined by multivariable logistic regression analysis

SNP		Dominant		Recessive		Additive 1		Additive 2	
		*P*	OR (95% CI)	*P*	OR (95% CI)	*P*	OR (95% CI)	*P*	OR (95% CI)
Hypertriglyceridemia									
rs10790162	G/A	<1.0 × 10^−23^	1.48 (1.38–1.59)	1.55 × 10–12	1.62 (1.42–1.85)	5.86 × 10–19	1.41 (1.31–1.53)	5.22 × 10–19	1.87 (1.63–2.15)
rs7350481	C/T	<1.0 × 10^−23^	1.50 (1.40–1.61)	9.45 × 10–11	1.54 (1.35–1.75)	1.62 × 10–21	1.45 (1.34–1.56)	6.31 × 10–18	1.81 (1.58–2.07)
Hypo–HDL-cholesterolemia									
rs146515657	T/C (N650S)	<1.0 × 10^−23^	35.37 (15.61–95.21)	ND		<1.0 × 10–23	35.37 (15.61–95.21)	ND	
rs147317864	C/T (A262T)	2.26 × 10^−14^	1.30 × 109 (ND)	ND		2.26 × 10–14	1.30 × 109 (ND)	ND	
rs12229654	T/G	5.45 × 10^−9^	1.30 (1.19–1.42)	0.0113	1.28 (1.06–1.53)	9.89 × 10–8	1.29 (1.17–1.41)	0.0005	1.41 (1.16–1.70)
Hyper–LDL-cholesterolemia									
rs7771335	A/G	8.86 × 10^−12^	1.29 (1.20–1.39)	7.50 × 10–7	1.45 (1.26–1.68)	2.48 × 10–8	1.24 (1.15–1.34)	3.81 × 10–9	1.58 (1.36–1.83)
rs76974938	C/T (D67N)	8.58 × 10^−13^	0.48 (0.39–0.59)	ND		8.58 × 10–13	0.48 (0.39–0.59)	ND	
rs2071653	C/T	8.58 × 10^−9^	1.23 (1.15–1.33)	7.00 × 10–8	1.42 (1.25–1.61)	2.20 × 10–5	1.18 (1.09–1.27)	4.26 × 10–10	1.52 (1.34–1.74)
rs2853969	C/T	2.11 × 10^−10^	1.34 (1.22–1.46)	0.0199	1.45 (1.06–1.96)	2.31 × 10–9	1.33 (1.21–1.45)	0.0080	1.53 (1.12–2.07)
rs2269704	C/T	3.83 × 10^−9^	1.28 (1.18–1.39)	0.1776		9.70 × 10–9	1.28 (1.18–1.39)	0.0707	
rs2269703	G/A	4.42 × 10^−9^	1.28 (1.18–1.39)	0.1911		1.05 × 10–8	1.28 (1.18–1.39)	0.0773	
rs495089	T/C	2.90 × 10^−6^	1.20 (1.11–1.29)	4.45 × 10–5	1.21 (1.11–1.33)	0.0005	1.15 (1.06–1.25)	1.87 × 10–7	1.32 (1.19–1.46)
rs2269702	A/G	2.74 × 10^−7^	1.22 (1.13–1.32)	0.0147	1.27 (1.05–1.54)	3.25 × 10–6	1.21 (1.11–1.30)	0.0027	1.35 (1.11–1.63)
rs1233399	C/T	3.11 × 10^−6^	0.84 (0.78–0.90)	0.0063	0.81 (0.69–0.94)	5.29 × 10–5	0.85 (0.79–0.92)	0.0006	0.76 (0.65–0.89)

Multivariable logistic regression analysis was performed with adjustment for age and sex. Based on Bonferroni's correction, *P* values of < 1.71 × 10^−4^ (0.05/292), of < 1.44 × 10^−4^ (0.05/348), or of < 1.10 × 10^−4^ (0.05/456) were considered statistically significant in the analysis of hypertriglyceridemia, hypo–HDL-cholesterolemia, or hyper–LDL-cholesterolemia, respectively, and are shown in bold. OR, odds ratio; CI, confidence interval; ND, not determined.

### Linkage disequilibrium and haplotype analysis

Given that eight SNPs (rs7771335, rs2071653, rs2853969, rs2269704, rs2269703, rs495089, rs2269702, rs1233399) associated with hyper–LDL-cholesterolemia were all located at chromosome 6p22.1-p21.3, we examined linkage disequilibrium among these SNPs as well as the relation of their haplotypes to this condition. The eight SNPs were all in strong linkage disequilibrium (Supplementary Table 11). Haplotype analysis revealed that the haplotypes A (rs7771335)–C (rs2071653)–C (rs2853969)–C (rs2269704)–G (rs2269703)–T (rs495089)–A (rs2269702)–T (rs1233399) and G (rs7771335)–T (rs2071653)–T (rs2853969)–T (rs2269704)–A (rs2269703)–C (rs495089)–G (rs2269702)–C (rs1233399) were significantly (*P* < 4.31 × 10^−4^) associated with hyper–LDL-cholesterolemia, with the former haplotype being protective against and the latter representing a risk factor for this condition (Supplementary Table 12).

### Relation of identified SNPs to serum triglyceride, HDL-cholesterol, or LDL-cholesterol levels

We examined the relation of genotypes of identified SNPs to serum triglyceride, HDL-cholesterol, or LDL-cholesterol levels by one-way analysis of variance. The 46 SNPs identified in the EWAS for serum triglyceride concentration, including the two SNPs also found to be associated with hypertriglyceridemia (rs10790162 of *BUD13*, rs7350481 at 11q23.3), were all significantly [*P* < 0.0011 (0.05/46)] associated with serum triglyceride level (Supplementary Table 13). The 104 SNPs identified in the EWAS for serum HDL-cholesterol concentration, including two of the three SNPs found to be associated with hypo–HDL-cholesterolemia (rs146515657 of *USP4*, rs12229654 at 12q24.1), were all significantly [*P* < 4.76 × 10^−4^ (0.05/105)] associated with the serum HDL-cholesterol level, as was the SNP associated with hypo–HDL-cholesterolemia alone (rs147317864 of *TRABD2B*) (Supplementary Table 14). The 40 SNPs identified in the EWAS for serum LDL-cholesterol concentration, including one SNP also found to be associated with hyper–LDL-cholesterolemia (rs2853969 at 6p21.3), were all significantly [*P* < 0.0010 (0.05/48)] associated with serum LDL-cholesterol level, as were five of the eight SNPs associated with hyper-LDL-cholesterolemia alone (rs7771335 at 6p22.1, rs2071653 of *MOG*, rs2269704 of *PPP1R18*, rs2269703 of *NRM*, rs2269702 of *MDC1*) (Supplementary Table 15).

### Relation of SNPs identified in the present study to dyslipidemia-related phenotypes examined in previous GWASs

We examined the relation of genes, chromosomal loci, and SNPs identified in the present study to dyslipidemia-related phenotypes included in previous GWASs deposited in GWAS Catalog (http://www.ebi.ac.uk/gwas). Among the 24 loci associated with triglyceride-related traits in the present study, 13 loci—*BUD13* [15], 11q23.3 [16], *APOA5* [15, 16], *ZPR1* [16], *APOA4* [15, 17], *LPL* [15, 17], 8p21.3 [15, 18], *SIK3* [19, 20], *GCKR* [16], 2p23 [17, 21], *C2orf16* [22], 8q24.1 [23], and *LOC101929011* [21]—were previously shown to be related to the circulating triglyceride level or hypertriglyceridemia (Supplementary Table 16). Among the 69 loci associated with HDL-cholesterol–related traits in the present study, 16 loci—12q24.1 [24], 16q13 [15, 16], *CETP* [15, 16], *APOA5* [16], *LIPC* [16, 17], *HECTD4* [24], *LILRB2* [8], *LPL* [15, 16], 8p21.3 [15, 16], *LOC101928635* [10, 17], *BUD13* [16], *ZPR1* [23], *ABCA1* [10, 16], 11q23.3 [16], *OAS3* [24], and *CD36* [25]—were previously shown to be related to the blood HDL-cholesterol level or hypo–HDL-cholesterolemia (Supplementary Table 17). Among the 32 loci associated with LDL-cholesterol–related traits in the present study, nine loci—*APOE* [26], *APOC1* [27], *APOB* [16, 17], *PCSK9* [17], *PSRC1* [12, 28], *CELSR2* [10, 16], 1p13.3 [16], *ABO* [17], and 9q34.2 [8, 17]—were previously shown to be related to the circulating LDL-cholesterol concentration or hyper–LDL-cholesterolemia (Supplementary Table 18).

## DISCUSSION

We found that two SNPs—rs10790162 of *BUD13* and rs7350481 at 11q23.3—were significantly related to both the serum triglyceride concentration and hypertriglyceridemia; three SNPs—rs146515657 of *USP4*, rs147317864 of *TRABD2B*, and rs12229654 at 12q24.1—were significantly related to both the serum HDL-cholesterol concentration and hypo–HDL-cholesterolemia; and six SNPs—rs2853969 at 6p21.3, rs7771335 at 6p22.1, rs2071653 of *MOG*, rs2269704 of *PPP1R18*, rs2269703 of *NRM*, and rs2269702 of *MDC1*—were significantly related to both the serum LDL-cholesterol concentration and hyper–LDL-cholesterolemia. Among these SNPs, rs146515657 of *USP4* and rs147317864 of *TRABD2B* may be novel determinants of hypo–HDL-cholesterolemia, whereas rs2853969 at 6p21.3, rs7771335 at 6p22.1, rs2071653 of *MOG*, rs2269704 of *PPP1R18*, rs2269703 of *NRM*, and rs2269702 of *MDC1* may be new determinants of hyper–LDL-cholesterolemia. We also found that 12, 61, 23, and 3 SNPs may be new determinants of the serum triglyceride, HDL-cholesterol, or LDL-cholesterol concentrations or of hyper–LDL-cholesterolemia, respectively.

### SNPs associated with serum HDL-cholesterol or LDL-cholesterol levels

The ubiquitin specific peptidase 4 gene (*USP4*) is located at chromosome 3p21.31 (NCBI Gene, https://www.ncbi.nlm.nih.gov/gene) and is expressed in various tissues and organs (The Human Protein Atlas, http://www.proteinatlas.org). Ubiquitin-specific proteases promote posttranslational protein modification by reversing protein ubiquitination and thereby activates multiple biological processes including the cell cycle, DNA repair, and intracellular signaling [29]. *USP4* accelerates the growth, invasion, and metastasis of colorectal cancer [29, 30]. We have now shown that rs146515657 [T/C (N650S)] of *USP4* was significantly associated with both the serum HDL-cholesterol concentration and hypo–HDL-cholesterolemia, with the minor *C* allele being related to a lower serum level of HDL-cholesterol, although the molecular mechanism underlying this association remains unclear.

The TraB domain containing 2B gene (*TRABD2B*) is located at chromosome 1p33 (NCBI Gene) and is expressed ubiquitously (The Human Protein Atlas). *TRABD2B* encodes the metalloprotease TIKI2 that inhibits the Wnt/β-catenin signaling by cleavage of the amino terminus of Wnt protein [31]. The expression of TIKI2 was reduced in osteosarcoma specimens and the increased expression of TIKI2 inhibited the growth of osteosarcoma *in vivo*, suggesting TIKI2 suppresses the growth of osteosarcoma [32]. We have now shown that rs147317864 [C/T (A262T)] of *TRABD2B* was significantly associated with both the serum concentration of HDL-cholesterol and hypo–HDL-cholesterolemia, with the minor *T* allele being related to a reduced level of serum HDL-cholesterol, although the functional relevance underlying this association remains unclear.

The myelin oligodendrocyte glycoprotein gene (*MOG*) is located at chromosome 6p22.1 (NCBI Gene) and is highly expressed in brain (The Human Protein Atlas). The MOG protein is localized to the outer surface of the myelin sheath of neurons in the central nervous system and is a key antigen for autoimmune responses that result in inflammation and demyelination [33, 34]. Both B cell responses and antibodies to MOG have also been detected in patients with demyelinating diseases such as multiple sclerosis and acute disseminating encephalomyelitis [34]. We have now shown that rs2071653 (C/T) of *MOG* was significantly associated with both the serum LDL-cholesterol concentration and hyper–LDL-cholesterolemia, with the minor *T* allele being related to an increased serum level of LDL-cholesterol, although the underlying molecular mechanism remains unknown.

The protein phosphatase 1 regulatory subunit 18 gene (*PPP1R18*) is located at chromosome 6p21.33 (NCBI Gene) and is expressed in various tissues (The Human Protein Atlas). Protein phosphatase 1 binds to regulatory subunits that target the enzyme to different intracellular locations to exert its activity toward specific substrates [35]. The PPP1R18 protein is a regulatory subunit that targets protein phosphatase 1 to the F-actin cytoskeleton [36]. We have now shown that rs2269704 (C/T) of *PPP1R18* was significantly associated with both the serum LDL-cholesterol concentration and hyper–LDL-cholesterolemia, with the minor *T* allele being related to an increased serum LDL-cholesterol level, although the molecular mechanism underpinning this association is unclear.

The nurim gene (*NRM*) is located at chromosome 6p21.33 (NCBI Gene) and is expressed ubiquitously (The Human Protein Atlas). The NRM protein contains transmembrane domains and resides within the inner nuclear membrane, where it is tightly bound to the nuclear envelope [37]. *NRM* is expressed in a broad range of cancers, with its expression level being correlated with tumor grade [38]. NRM deficiency was found to alter the shape of the nuclear envelope and to enhance ultraviolet light–induced apoptosis in HeLa cells, implicating NRM in suppression of apoptosis [38]. We have now shown that rs2269703 (G/A) of *NRM* was significantly associated with both the serum LDL-cholesterol concentration and hyper–LDL-cholesterolemia, with the minor *A* allele being related to an increased serum LDL-cholesterol level, although the molecular mechanism is unknown.

The mediator of DNA damage checkpoint 1 gene (*MDC1*) is located at chromosome 6p21.33 (NCBI Gene) and is expressed in various tissues and organs (The Human Protein Atlas). MDC1 is a nuclear protein required for activation of the intra–S phase and G_2_-M phase checkpoints of the cell cycle in response to DNA damage [39]. We have now shown that rs2269702 (A/G) of *MDC1* was significantly associated with both the serum LDL-cholesterol concentration and hyper–LDL-cholesterolemia, with the minor *G* allele being related to an increased serum LDL-cholesterol level, although the molecular mechanism remains unclear.

We also found that rs2853969 (C/T) at 6p21.3 and rs7771335 (A/G) at 6p22.1 were significantly associated with both the serum LDL-cholesterol concentration and hyper–LDL-cholesterolemia, with the minor *T* and *G* alleles, respectively, being related to an increased serum level of LDL-cholesterol. Eight SNPs (rs7771335, rs2071653, rs2853969, rs2269704, rs2269703, rs495089, rs2269702, rs1233399) associated with hyper–LDL-cholesterolemia are all located at chromosomal region 6p22.1-p21.3 and were in strong linkage disequilibrium.

### General considerations

In previous GWASs of blood lipid traits in East Asian populations [24], a minor allele frequency (MAF) and effect size of identified SNPs were 10% to 33% and −0.088 to −0.050 mg/dL for triglycerides, 12% to 15% and −0.035 to 0.043 mg/dL for HDL-cholesterol, and 26% and 2.203 mg/dL for LDL-cholesterol, respectively. In trans-ancestry GWASs for lipid profiles [10], the MAF and effect size of identified SNPs ranged from 9% to 49% and from −0.033 to 0.037 mg/dL for triglycerides, from 9% to 50% and from −0.051 to 0.034 mg/dL for HDL-cholesterol, and from 4% to 48% and from −0.051 to 0.103 mg/dL for LDL-cholesterol. In more recent GWASs that included low-frequency or rare variants, the MAF and effect size of identified SNPs ranged from 1.76% to 3.25% and from −30% to 21% for triglycerides, from 0.20% to 2.01% and from −3 to 17 mg/dL for HDL-cholesterol, and from 0.05% to 3.43% and from −40 to 71 mg/dL for LDL-cholesterol [11]; as well as from 1% to 47% and from −0.170 to 0.128 mmol/L for triglycerides, from 5% to 20% and from −0.141 to 0.044 mmol/L for HDL-cholesterol, and from 1% to 21.6% and from −0.049 to 0.648 mmol/L for LDL-cholesterol, respectively [12]. Studies based on exome and whole-genome sequencing identified SNPs with a MAF and effect size from 0.06% to 28.2% and from −98.0 to 51.5 mg/dL, respectively, for LDL-cholesterol [13]; as well as those with a MAF of 0.007% to 4.6% and effect size of −65.3 to 12.0% for triglycerides and −0.087 to 0.40 mmol/L for HDL-cholesterol [14].

In our study, among 46 SNPs associated with the serum triglyceride concentration, one SNP was a rare variant (MAF, 0.3%) with a large effect size (difference in serum triglyceride level among genotypes, 28.1%), eight SNPs were low-frequency variants (1.2–3.3%) with a moderate to large effect size (14.6–19.8%), and 37 SNPs were common variants (7.3–47.6%) with a small to large effect size (6.2–45.2%) (Supplementary Table 19). Among 104 SNPs associated with the serum HDL-cholesterol concentration, nine SNPs were rare variants (MAF, 0.2–0.3%) with a large effect size (difference in serum HDL-cholesterol level among genotypes, 17.3–29.3%), 32 SNPs were low-frequency variants (0.5–4.5%) with a moderate to large effect size (5.2–25.8%), and 63 SNPs were common variants (5.0–47.6%) with a small to large effect size (3.3–18.7%) (Supplementary Table 20). Among 40 SNPs associated with the serum LDL-cholesterol concentration, three SNPs were low-frequency variants (MAF, 1.1–4.3%) with a large effect size (difference in serum LDL-cholesterol level among genotypes, 16.8–24.8%) and 37 SNPs were common variants (6.6–27.9%) with a small to moderate effect size (2.8–9.2%) (Supplementary Table 21).

### Study limitations

There are several limitations to the present study. (1) Our results were not replicated and will therefore require validation in independent subject panels or in other ethnic groups. (2) Subjects who had treatment for other diseases such as diabetes mellitus were included in the study. It was possible that such treatment affected lipid profiles of the subjects. (3) SNPs identified in our study might be in linkage disequilibrium with other polymorphisms in the nearby genes that are actually responsible for the observed associations. (4) Three SNPs associated with hyper–LDL-cholesterolemia were not significantly related to the serum LDL-cholesterol concentration, which may be attributable to the effects of medical treatment. (5) The functional relevance of the observed associations remains to be determined.

## MATERIALS AND METHODS

### Study subjects

A total of 14,337 subjects (8354 individuals with dyslipidemia, 5983 controls) was recruited as described previously [40].

Venous blood was collected in the early morning after the subjects had fasted overnight. Blood samples were centrifuged at 1600 × *g* for 15 min at 4°C, and serum was separated for subsequent analysis. Serum concentrations of triglycerides, HDL-cholesterol, and LDL-cholesterol were measured at the clinical laboratory of each hospital. The 4742 subjects with hypertriglyceridemia and 8672 controls had serum triglyceride concentrations of ≥ 1.69 mmol/L (range, 1.69 to 20.14 mmol/L) and < 1.69 mmol/L (0.14 to 1.68 mmol/L), respectively; the 2646 subjects with hypo–HDL-cholesterolemia and 11,473 controls had serum HDL-cholesterol concentrations of < 1.03 mmol/L (0.26 to 1.01 mmol/L) and ≥1.03 mmol/L (1.03 to 4.73 mmol/L), respectively; and the 4489 subjects with hyper–LDL-cholesterolemia and 9088 controls had serum LDL-cholesterol concentrations of ≥ 3.62 mmol/L (3.62 to 12.31 mmol/L) and < 3.62 mmol/L (0.26 to 3.59 mmol/L), respectively. Individuals with dyslipidemia had at least one of hypertriglyceridemia, hypo–HDL-cholesterolemia, and hyper–LDL-cholesterolemia, or were taking anti-dyslidemic medications. The 1300 subjects with both hypertriglyceridemia and hypo–HDL-cholesterolemia as well as 7844 controls overlapped between the corresponding studies, as did the 2002 subjects with both hypertriglyceridemia and hyper–LDL-cholesterolemia and 6326 controls as well as the 712 subjects with both hypo–HDL-cholesterolemia and hyper–LDL-cholesterolemia and 7776 controls. Individuals with single-gene disorders such as familial hypercholesterolemia or with endocrinologic or metabolic diseases that cause dyslipidemia were excluded from the study. Those taking medications that may cause secondary dyslipidemia were also excluded. Autopsy cases were excluded from controls.

The study protocol complied with the Declaration of Helsinki and was approved by the Committees on the Ethics of Human Research of Mie University Graduate School of Medicine, Tokyo Metropolitan Institute of Gerontology, Hirosaki University Graduate School of Medicine, and participating hospitals. Written informed consent was obtained from each participant or families of the deceased subjects.

### EWASs

Methods for collection and extraction of genomic DNA samples were described previously [40]. EWASs for the serum concentrations of triglycerides (13,414 subjects), HDL-cholesterol (14,119 subjects), or LDL-cholesterol (13,577 subjects) or for hypertriglyceridemia (4742 cases, 8672 controls), hypo–HDL-cholesterolemia (2646 cases, 11,473 controls), or hyper-LDL-cholesterolemia (4489 cases, 9088 controls) were performed with HumanExome-12 v1.1 or v1.2 DNA Analysis BeadChip or Infinium Exome-24 v1.0 BeadChip arrays (Illumina, San Diego, CA). Detailed information of the exome arrays and methods of quality control were described previously [40]. Totals of 41,371, 41,225, and 41,347 SNPs passed quality control in the EWASs for hypertriglyceridemia, hypo–HDL-cholesterolemia, and hyper–LDL-cholesterolemia, respectively, and were included in the analysis.

### Statistical analysis

The relation of SNP genotypes to the serum concentrations of triglycerides, HDL-cholesterol, or LDL-cholesterol in the EWASs was examined with linear regression analysis. For analysis of characteristics of the study subjects, quantitative and categorical data were compared between cases and controls with the unpaired Student's *t* test and Fisher's exact test, respectively. Allele frequencies were estimated by the gene counting method, and departure from Hardy-Weinberg equilibrium was identified with Fisher's exact test. The relation of allele frequencies of SNPs to hypertriglyceridemia, hypo–HDL-cholesterolemia, or hyper–LDL-cholesterolemia in the EWASs was examined with Fisher's exact test. To compensate for multiple comparisons of genotypes or allele frequencies with lipid concentrations or dyslipidemia, we applied Bonferroni's correction for statistical significance of association. Given that 41,225 to 41,371 SNPs were analyzed, a *P* value of < 1.21 × 10^−6^ [0.05/(41,225 to 41,371] was considered statistically significant for the EWASs. Quantile-quantile plots for *P* values of genotypes or allele frequencies in the EWASs are shown in Supplementary Figures 3 and 4, respectively. The inflation factor (λ) was 1.05 for serum triglycerides, 0.97 for serum HDL-cholesterol, 1.06 for serum LDL-cholesterol, 1.20 for hypertriglyceridemia, 1.29 for hypo–HDL-cholesterolemia, and 1.20 for hyper–LDL-cholesterolemia. Multivariable logistic regression analysis was performed with hypertriglyceridemia, hypo–HDL-cholesterolemia, or hyper–LDL-cholesterolemia as a dependent variable and independent variables including age, sex (0, woman; 1, man), and genotype of each SNP. A detailed method of analysis was described previously [40]. Relations of genotypes of identified SNPs to serum concentrations of triglycerides, HDL-cholesterol, or LDL-cholesterol were examined by one-way analysis of variance. Bonferroni's correction was also applied to other statistical analysis as indicated. Statistical tests were performed with JMP Genomics version 6.0 software (SAS Institute, Cary, NC).

## CONCLUSIONS

The SNPs rs146515657 of *USP4* and rs147317864 of *TRABD2B* may be novel determinants of hypo–HDL-cholesterolemia whereas rs2853969 at 6p21.3, rs7771335 at 6p22.1, rs2071653 of *MOG*, rs2269704 of *PPP1R18*, rs2269703 of *NRM*, and rs2269702 of *MDC1* may be new determinants of hyper–LDL-cholesterolemia. In addition, 12, 61, 23, or 3 SNPs may be new determinants of the serum triglyceride, HDL-cholesterol, or LDL-cholesterol concentrations or of hyper–LDL-cholesterolemia, respectively. Determination of genotypes for these SNPs may prove informative for assessment of the genetic risk for dyslipidemia in Japanese.

## SUPPLEMENTARY TABLES AND FIGURES








































