# Supplementary material for: Identification of eight genetic variants as novel determinants of dyslipidemia in Japanese by exome-wide association studies
Source: Oncotarget. 2017 Apr 17;8(24):38950–61. doi: 10.18632/oncotarget.17159 (PMC5503585; doi:10.18632/oncotarget.17159)
Supplement: Supplementary file 1 [file oncotarget-08-38950-s001.pdf]

Identification of eight genetic variants as novel determinants of dyslipidemia in Japanese by exome-wide association studies

Supplementary Materials

Supplementary Table 1: The 104 SNPs significantly ($P < 1.21 \times 10^{-6}$) associated with serum HDL-cholesterol concentration in the EWAS. See Supplementary_Table_1

Supplementary Table 2: The 73 SNPs significantly ($P < 1.21 \times 10^{-6}$) associated with hypertriglyceridemia in the EWAS. See Supplementary_Table_2

Supplementary Table 3: The 87 SNPs significantly ($P < 1.21 \times 10^{-6}$) associated with hypo–HDL-cholesterolemia in the EWAS. See Supplementary_Table_3

Supplementary Table 4: The 114 SNPs significantly ($P < 1.21 \times 10^{-6}$) associated with hyper–LDL-cholesterolemia in the EWAS. See Supplementary_Table_4

Supplementary Table 5: Genotype distributions for SNPs associated ($P < 1.21 \times 10^{-6}$) with hypertriglyceridemia in the EWAS. See Supplementary_Table_5

Supplementary Table 6: Genotype distributions for SNPs associated ($P < 1.21 \times 10^{-6}$) with hypo-HDL-cholesterolemia in the EWAS. See Supplementary_Table_6

Supplementary Table 7: Genotype distributions for SNPs associated ($P < 1.21 \times 10^{-6}$) with hyper–LDL-cholesterolemia in the EWAS. See Supplementary_Table_7

Supplementary Table 8: Relation of SNPs to hypertriglyceridemia as determined by multivariable logistic regression analysis. See Supplementary_Table_8

Supplementary Table 9: Relation of SNPs to hypo–HDL-cholesterolemia as determined by multivariable logistic regression analysis. See Supplementary_Table_9

Supplementary Table 10: Relation of SNPs to hyper–LDL-cholesterolemia as determined by multivariable logistic regression analysis. See Supplementary_Table_10

Supplementary Table 11: Linkage disequilibrium of rs7771335, rs2071653, rs2853969, rs2269704, rs2269703, rs495089, rs2269702, and rs1233399

	rs7771335	rs2071653	rs2853969	rs2269704	rs2269703	rs495089	rs2269702	rs1233399
rs7771335		$< 1.0 \times 10^{-40}$	$< 1.0 \times 10^{-40}$	$< 1.0 \times 10^{-40}$	$< 1.0 \times 10^{-40}$	$< 1.0 \times 10^{-40}$	$< 1.0 \times 10^{-40}$	$< 1.0 \times 10^{-40}$
rs2071653	0.9715		$< 1.0 \times 10^{-40}$	$< 1.0 \times 10^{-40}$	$< 1.0 \times 10^{-40}$	$< 1.0 \times 10^{-40}$	$< 1.0 \times 10^{-40}$	$< 1.0 \times 10^{-40}$
rs2853969	0.8538	0.8827		$< 1.0 \times 10^{-40}$	$< 1.0 \times 10^{-40}$	$< 1.0 \times 10^{-40}$	$< 1.0 \times 10^{-40}$	$< 1.0 \times 10^{-40}$
rs2269704	0.6478	0.6528	0.9294		$< 1.0 \times 10^{-40}$	$< 1.0 \times 10^{-40}$	$< 1.0 \times 10^{-40}$	$< 1.0 \times 10^{-40}$
rs2269703	0.6473	0.6523	0.9293	1		$< 1.0 \times 10^{-40}$	$< 1.0 \times 10^{-40}$	$< 1.0 \times 10^{-40}$
rs495089	0.6874	0.5053	0.8068	0.542	0.5428		$< 1.0 \times 10^{-40}$	$< 1.0 \times 10^{-40}$
rs2269702	0.4153	0.3992	0.9319	1	0.9984	0.3755		$< 1.0 \times 10^{-40}$
rs1233399	−0.9791	−0.9387	−0.9009	−0.3926	−0.3941	−0.3095	−0.4554	

Lower left data are pairwise linkage disequilibrium coefficients (D'). Upper right data are *P* values.

Supplementary Table 12: Relations of haplotypes of eight SNPs to hyper–LDL-cholesterolemia

Haplotype	Overall frequency	Frequency		Chi-square *P* value	Permutation *P* value
		Controls	Hyper–LDL-cholesterolemia		
A-C-C-C-G-T-A-C	0.3005	0.3022	0.2978	0.5307	0.534
A-C-C-C-G-T-A-T	0.1480	0.1586	0.1307	**7.57×10^{-8}**	**$<1.0 \times 10^{-12}$**
A-C-C-C-G-C-A-C	0.1269	0.1292	0.1234	0.2332	0.300
G-T-C-C-G-C-A-C	0.1053	0.1011	0.1121	0.0142	0.021
G-T-T-T-A-C-G-C	0.0841	0.0754	0.0985	**1.23×10^{-8}**	**$<1.0 \times 10^{-12}$**
A-C-C-C-G-C-A-T	0.0581	0.0577	0.0587	0.7698	0.789
A-T-C-C-G-T-A-C	0.0350	0.0375	0.0313	0.0212	0.035
A-C-C-C-G-T-G-C	0.0261	0.0259	0.0264	0.8300	0.853
G-T-C-C-G-T-A-C	0.0225	0.0207	0.0250	0.0464	0.090
A-C-C-C-G-C-G-C	0.0153	0.0152	0.0155	0.8672	0.887
A-C-C-T-A-T-G-T	0.0120	0.0114	0.0128	0.3774	0.483
G-T-T-T-A-T-G-C	0.0101	0.0095	0.0110	0.3054	0.422
A-C-C-T-A-C-G-T	0.0080	0.0078	0.0085	0.5994	0.657
A-C-C-T-A-T-G-C	0.0067	0.0070	0.0062	0.5411	0.651
G-T-C-T-A-T-G-C	0.0060	0.0064	0.0054	0.4114	0.492
A-T-C-C-G-C-A-C	0.0042	0.0045	0.0038	0.4980	0.649
A-C-C-T-A-C-G-C	0.0040	0.0032	0.0050	0.0485	0.135
G-C-C-C-G-T-A-C	0.0031	0.0030	0.0035	0.5159	0.590
A-T-C-C-G-T-A-T	0.0027	0.0022	0.0032	0.2031	0.373
A-C-C-C-G-T-G-T	0.0027	0.0031	0.0020	0.1453	0.319
A-C-T-T-A-C-G-C	0.0026	0.0029	0.0021	0.2581	0.352
G-T-C-T-A-C-G-C	0.0024	0.0024	0.0024	0.9933	0.999
A-C-T-C-G-C-A-C	0.0021	0.0021	0.0020	0.9293	0.956
A-C-T-C-G-C-A-T	0.0015	0.0017	0.0015	0.7567	0.816
A-T-T-T-A-C-G-C	0.0015	0.0014	0.0015	0.8015	0.835
A-T-C-C-G-C-A-T	0.0012	0.0013	0.0010	0.5553	0.727
A-C-C-C-G-C-G-T	0.0010	0.0006	0.0018	0.0131	0.102

Haplotypes consist of rs7771335 (A/G), rs2071653, (C/T), rs2853969 (C/T), rs2269704 (C/T), rs2269703 (G/A), rs495089 (T/C), rs2269702 (A/G), and rs1233399, (C/T). Although eight SNPs give rise to 58 haplotypes, those with an overall haplotype frequency of < 0.001 are not shown. Based on Bonferroni's correction, *P* values of $< 4.31 \times 10^{-4}$ (0.05/116) were considered statistically significant and are shown in bold.

Supplementary Table 13: Relation of SNPs to the serum concentration of triglycerides. See Supplementary_Table_13

Supplementary Table 14: Relation of SNPs to the serum concentration of HDL-cholesterol. See Supplementary_Table_14

Supplementary Table 15: Relation of SNPs to the serum concentration of LDL-cholesterol. See Supplementary_Table_15

Supplementary Table 16: Relation of genes, loci, and SNPs identified in the present analysis of serum triglycerides to phenotypes previously examined in GWASs. See Supplementary_Table_16

Supplementary Table 17: Relation of genes, loci, and SNPs identified in the present analysis of serum HDL–cholesterol to phenotypes examined in previous GWASs. See Supplementary_Table_17

Supplementary Table 18: Relation of genes, loci, and SNPs identified in the present analysis of serum LDL-cholesterol to phenotypes previously examined in GWASs. See Supplementary_Table_18

Supplementary Table 19: Minor allele frequencies and effect sizes of the 46 SNPs associated with serum concentrations of triglycerides in the present study. See Supplementary_Table_19

Supplementary Table 20: Minor allele frequencies and effect sizes of the 104 SNPs associated with serum concentrations of HDL-cholesterol in the present study. See Supplementary_Table_20

Supplementary Table 21: Minor allele frequencies and effect sizes of the 40 SNPs associated with serum concentrations of LDL-cholesterol in the present study. See Supplementary_Table_21

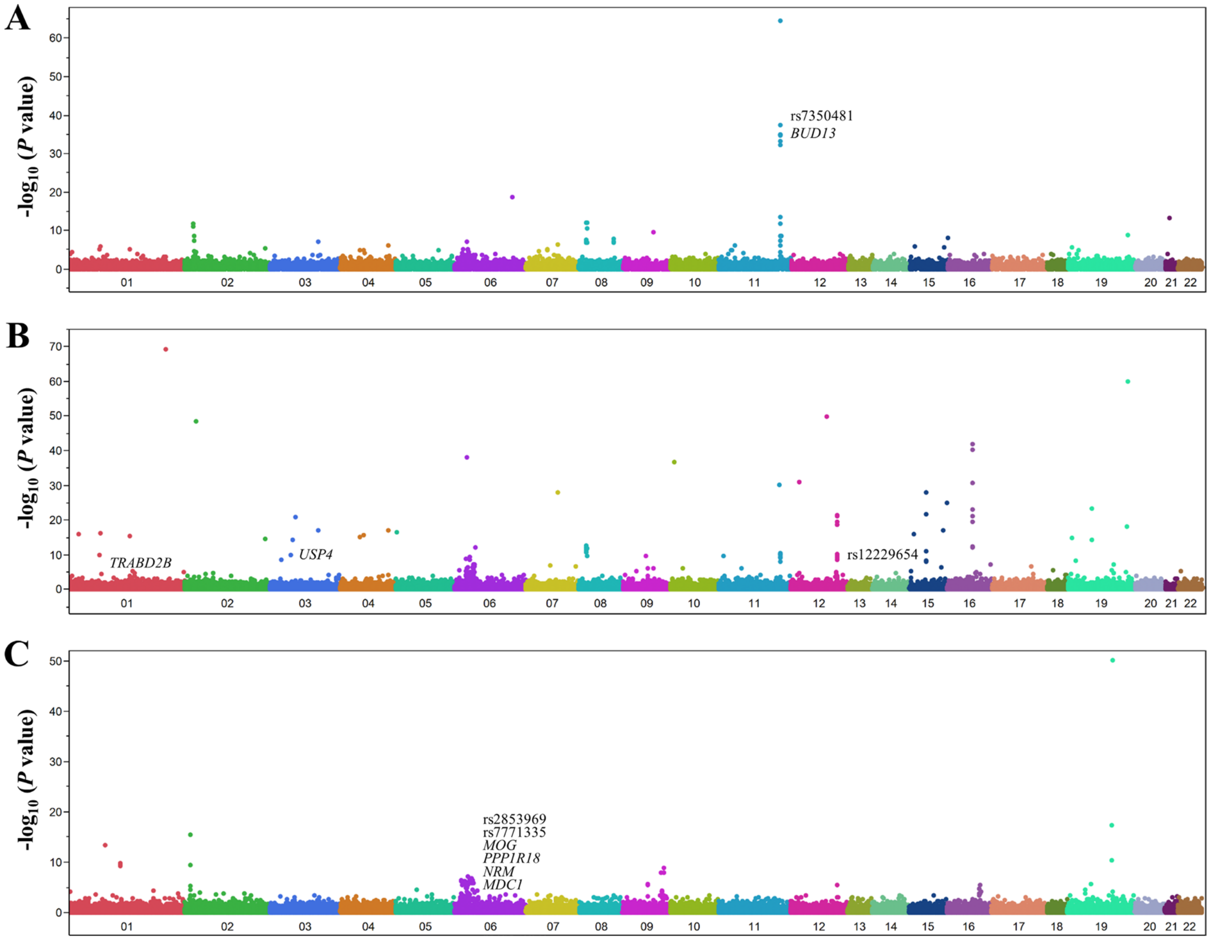

Supplementary Figure 1: Manhattan plots for *P* values in the EWASs for the serum concentrations of triglycerides (**A**), HDL-cholesterol (**B**), or LDL-cholesterol (**C**). The *P* values (*y*-axis) are plotted as $-\log_{10}(P)$ with respect to the physical chromosomal position of the corresponding SNPs (*x*-axis). SNPs or genes found to be associated with the serum concentrations of triglycerides, HDL-cholesterol, or LDL-cholesterol are indicated in (A), (B), or (C), respectively.

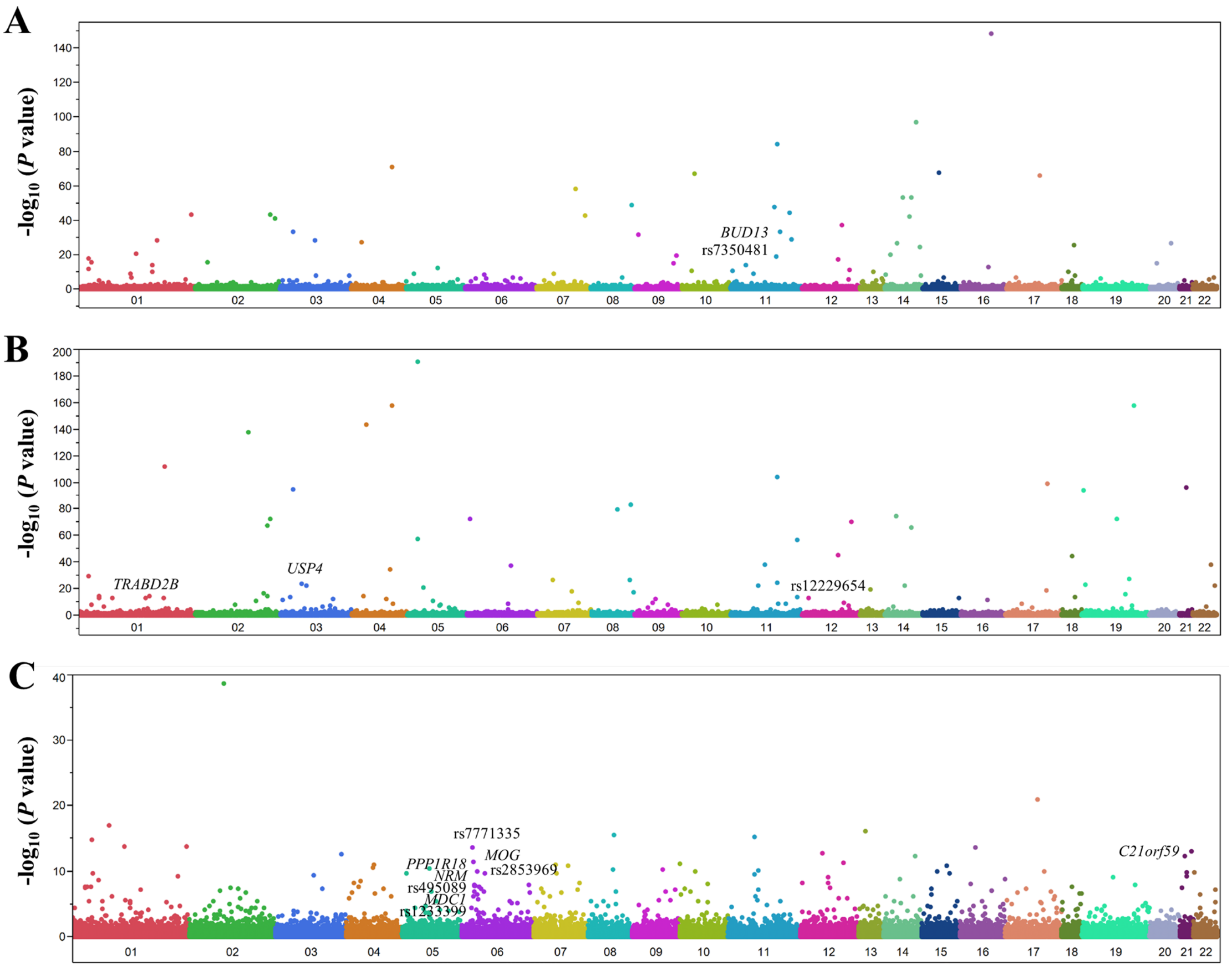

Supplementary Figure 2: Manhattan plots for *P* values in the EWASs for hypertriglyceridemia (**A**), hypo–HDL-cholesterolemia (**B**), or hyper–LDL-cholesterolemia (**C**). The *P* values (*y*-axis) are plotted as $-\log_{10}(P)$ with respect to the physical chromosomal position of the corresponding SNPs (*x*-axis). SNPs or genes found to be associated with hypertriglyceridemia, hypo–HDL-cholesterolemia, or hyper–LDL-cholesterolemia are indicated in (A), (B), or (C), respectively.

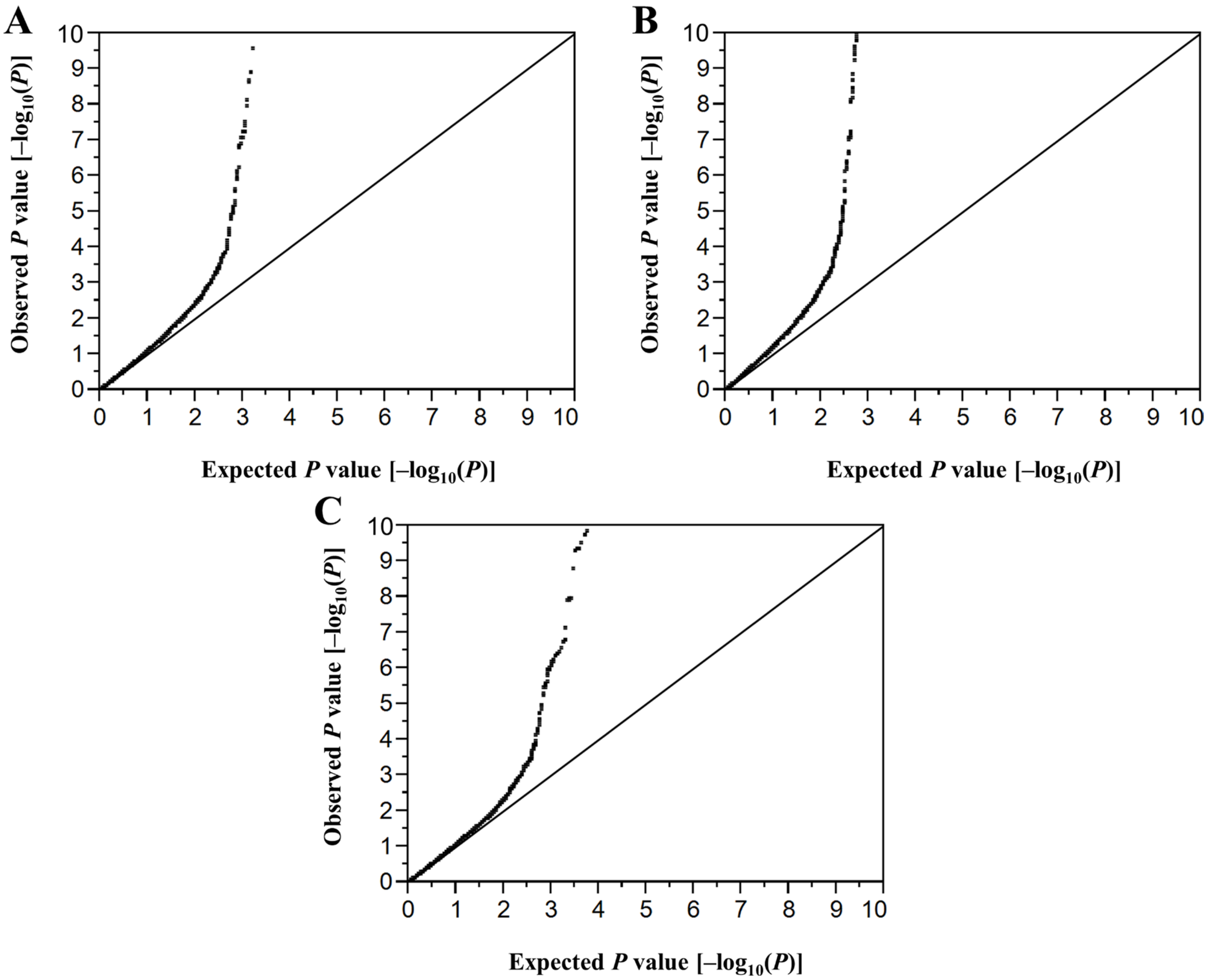

Supplementary Figure 3: Quantile-quantile plots for *P* values of genotype distributions in the EWASs for the serum concentrations of triglycerides (**A**), HDL-cholesterol (**B**), or LDL-cholesterol (**C**). The observed *P* values (*y*-axis) were compared with the expected *P* values (*x*-axis) under the null hypothesis, with the values being plotted as $-\log_{10}(P)$.

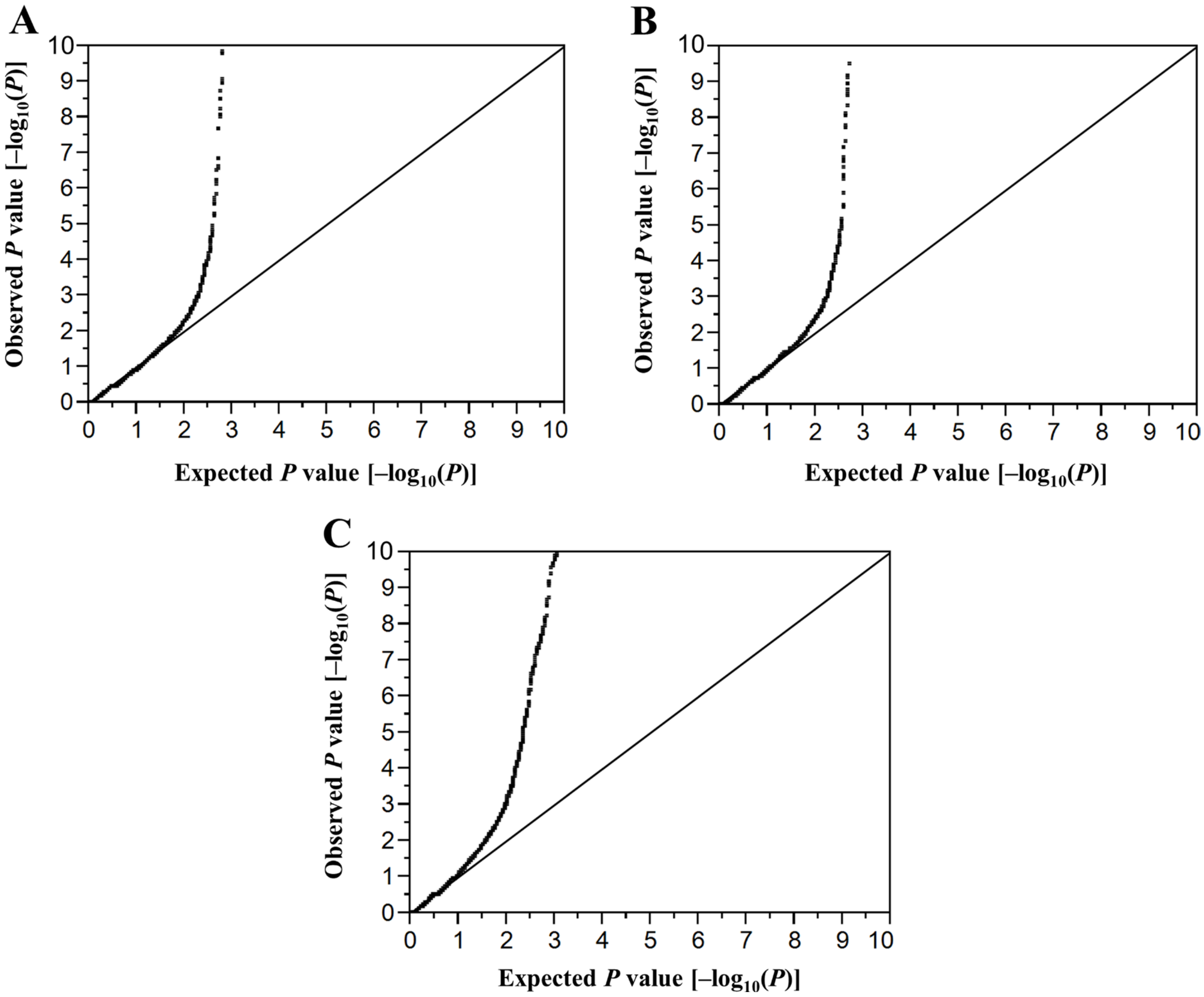

Supplementary Figure 4: Quantile-quantile plots for *P* values of allele frequencies in the EWASs for hypertriglyceridemia (**A**), hypo–HDL-cholesterolemia (**B**), or hyper–LDL-cholesterolemia (**C**). The observed *P* values (*y*-axis) were compared with the expected *P* values (*x*-axis) under the null hypothesis, with the values being plotted as $–\log_{10}(P)$.
