# Supplementary material for: Identification of eight genetic variants as novel determinants of dyslipidemia in Japanese by exome-wide association studies
Source: Oncotarget. 2017 Apr 17;8(24):38950–61. doi: 10.18632/oncotarget.17159 (PMC5503585; doi:10.18632/oncotarget.17159)
Supplement: Supplementary file 2 [file oncotarget-08-38950-s002.docx]

**Supplementary Table 1.** The 104 SNPs significantly (*P* < 1.21 × 10^–6^) associated with serum HDL-cholesterol concentration in the EWAS

___________________________________________________________________________

Gene dbSNP Nucleotide Chromosome: MAF *P*

(amino acid) position (%) (genotype)

substitution*^a^*

___________________________________________________________________________

*LPGAT1* rs150552771 T/C (K200E) 1: 211783358 5.0 4.56 × 10^–70^

*LAIR2* rs34429135 T/A (F115Y) 19: 54508164 2.5 1.11 × 10^–60^

*KRR1* rs17115182 G/A (P43S) 12: 75508405 7.0 1.19 × 10^–50^

*EHD3* rs116417209 G/A (V151I) 2: 31249417 3.5 2.63 × 10^–49^

rs3764261 G/T 16: 56959412 19.8 1.39 × 10^–42^

rs247616 C/T 16: 56955678 19.7 6.53 × 10^–41^

rs9261800 C/G 6: 30408822 2.8 5.89 × 10^–39^

*DCLRE1C* rs150854849 C/T (R179Q) 10: 14934704 2.4 1.78× 10^–37^

*STYK1* rs138533962 G/A (R379C) 12: 10620278 2.0 8.69 × 10^–32^

*CETP* rs2303790 A/G (D459G) 16: 56983380 3.6 2.14 × 10^–31^

*APOA5* rs2075291 C/A (G185C) 11: 116790676 7.3 5.09 × 10^–31^

*MUC17* rs78010183 A/T (T1305S) 7: 101035329 1.8 1.01 × 10^–28^

*LIPC* rs1800588 T/C 15: 58431476 49.9 1.04 × 10^–28^

*OR4F6* rs141569282 G/A (A117T) 15: 101806068 1.7 8.73 × 10^–26^

*CYP4F8* rs201166643 C/A (R488S) 19: 15629257 1.1 4.51 × 10^–24^

*CETP* rs1532624 G/T 16: 56971567 29.6 8.51 × 10^–24^

*LIPC* rs261334 G/C 15: 58434545 46.1 1.90 × 10^–22^

*ACAD10* rs11066015 G/A 12: 111730205 27.5 3.58 × 10^–22^

*ALDH2* rs671 G/A (E504K) 12: 111803962 27.6 7.77 × 10^–22^

rs173539 C/T 16: 56954132 30.0 9.59 × 10^–22^

*CACNA1D* rs35874056 G/A (G460S) 3: 53702798 2.0 1.12 × 10^–21^

*CETP* rs9939224 G/T 16: 56968820 9.5 3.09 × 10^–20^

*BRAP* rs3782886 A/G 12: 111672685 29.3 3.31× 10^–20^

*HECTD4* rs2074356 C/T 12: 112207597 25.4 2.05 × 10^–19^

*HECTD4* rs11066280 T/A 12: 112379979 29.0 2.30 × 10^–19^

*LILRB2* rs73055442 C/T (R103H) 19: 54279838 1.6 5.32 × 10^–19^

*COL6A5* rs200982668 G/A (E2501K) 3: 130470894 1.3 7.82 × 10^–18^

*VPS33B* rs199921354 C/T (R80Q) 15: 91013841 1.2 8.52 × 10^–18^

*MARCH1* rs61734696 G/T (Q137K) 4: 164197303 1.2 1.21 × 10^–17^

*SLC9A3* rs143027124 C/T (V213I) 5: 488354 1.1 3.56 × 10^–17^

*MOB3C* rs139537100 C/T (R24Q) 1: 46615006 1.2 6.40 × 10^–17^

*PRAMEF12* rs199576535 G/A (V341I) 1: 12777168 1.0 8.37 × 10^–17^

*PLCB2* rs200787930 C/T (E1095K) 15: 40289298 1.2 1.18 × 10^–16^

*CXCL8* rs188378669 G/T 4: 73741568 1.2 2.08 × 10^–16^

*TMOD4* rs115287176 G/A (R277W) 1: 151170961 1.2 4.08 × 10^–16^

*ADGRL3* rs192210727 G/T (R580I) 4: 61909615 1.3 5.54 × 10^–16^

*ZNF77* rs146879198 G/A (R340*) 19: 2934109 1.2 1.07 × 10^–15^

*COL6A3* rs146092501 C/T (E1386K) 2: 237371861 1.2 2.87 × 10^–15^

*IQCF1* rs200134435 G/A (R103W) 3: 51895201 0.8 4.19 × 10^–15^

*CYP4F12* rs609636 G/A (D76N) 19: 15678288 2.3 6.39 × 10^–15^

*LPL* rs15285 G/A 8: 19967156 19.2 2.29 × 10^–13^

*LPL* rs13702 A/G 8: 19966981 19.2 2.75 × 10^–13^

*CETP* rs7499892 C/T 16: 56972678 17.2 3.14 × 10^–13^

*CETP* rs1800775 A/C 16: 56961324 45.0 5.54 × 10^–13^

rs7773955 C/T 6: 32738942 26.3 6.61 × 10^–13^

*LPL* rs326 A/G 8: 19961928 19.4 7.48 × 10^–13^

rs2197089 C/T 8: 19968862 27.6 8.91 × 10^–13^

rs2083637 T/C 8: 20007664 19.0 1.24 × 10^–12^

*LPL* rs301 T/C 8: 19959423 19.3 1.47 × 10^–12^

rs1441756 T/G 8: 20010875 19.0 1.52 × 10^–12^

rs17482753 G/T 8: 19975135 12.6 5.30 × 10^–12^

rs10096633 C/T 8: 19973410 12.7 6.29 × 10^–12^

LOC101928635 rs1532085 A/G 15: 58391167 42.1 8.97 × 10^–12^

*LPL* rs328 C/G (S474*) 8: 19962213 12.9 1.49 × 10^–11^

rs10503669 C/A 8: 19990179 12.6 1.80 × 10^–11^

rs12678919 A/G 8: 19986711 12.6 1.94 × 10^–11^

*APOA5* rs2266788 T/C 11: 116789970 26.2 2.64 × 10^–11^

*NAA25* rs12231744 C/T (R876K) 12: 112039251 35.1 4.92 × 10^–11^

*BUD13* rs10790162 G/A 11: 116768388 26.3 5.95 × 10^–11^

*PTCH2* rs147284320 C/T (V503I) 1: 44828589 2.0 8.88 × 10^–11^

*ZPR1* rs964184 C/G 11: 116778201 26.3 1.16 × 10^–10^

*USP4* rs146515657 T/C (N650S) 3: 49292533 0.5 1.54 × 10^–10^

*OR52I1* rs200585398 A/G (M167V) 11: 4594537 0.5 1.77 × 10^–10^

*ABCA1* rs1883025 G/A 9: 104902020 28.8 2.30 × 10^–10^

rs7016880 G/C 8: 20019235 12.0 2.36 × 10^–10^

*ATXN2* rs7969300 T/C (N248S) 12: 111555908 38.8 2.65 × 10^–10^

rs9326246 G/C 11: 116741017 26.5 3.49 × 10^–10^

*TCF19* rs61733202 G/A (G26R) 6: 31159545 0.2 4.21 × 10^–10^

*ZPR1* rs2075290 T/C 11: 116782580 26.7 6.21 × 10^–10^

*OAS3* rs2072134 C/T 12: 112971371 17.6 6.32 × 10^–10^

*LOC554223* rs1610640 A/G 6: 29790748 41.6 1.48 × 10^–9^

rs12229654 T/G 12: 110976657 22.5 2.02 × 10^–9^

*HLA-B* rs1058026 T/G 6: 31353908 33.4 2.22 × 10^–9^

*PLCD1* rs147186786 C/T (R268Q) 3: 38010550 0.2 3.46 × 10^–9^

LOC101928635 rs10468017 C/T 15: 58386313 20.3 4.61 × 10^–9^

*DENND1C* rs200449136 G/A 19: 6478852 0.3 4.79 × 10^–9^

LOC101928635 rs2043085 A/G 15: 58388755 40.9 6.72 × 10^–9^

rs7350481 C/T 11: 116715567 27.7 7.38 × 10^–9^

LOC101928635 rs4775041 G/C 15: 58382496 20.2 8.88 × 10^–9^

*LOC101929163* rs3129945 G/A 6: 32374760 33.6 6.01 × 10^–8^

*ANKRD11* rs139088883 G/A (A1840V) 16: 89281023 0.3 7.62 × 10^–8^

*BTNL2* rs2076528 T/G 6: 32396417 23.0 7.99 × 10^–8^

*BTNL2* rs3763315 G/T 6: 32408877 23.0 8.13 × 10^–8^

*BTNL2* rs41441651 C/T (D336N) 6: 32396111 23.0 8.31 × 10^–8^

*BTNL2* rs28362675 C/A (E454*) 6: 32394744 23.0 8.31 × 10^–8^

*BTNL2* rs41417449 T/C (M295V) 6: 32396234 23.0 8.33 × 10^–8^

*BTNL2* rs78587369 G/A (T165I) 6: 32403150 23.0 8.35 × 10^–8^

*APOE* rs7412 C/T (R176C) 19: 44908822 4.3 8.36 × 10^–8^

*BTNL2* rs34423804 T/A (V283D) 6: 32396269 23.0 8.75 × 10^–8^

*CD36* rs75326924 C/T (P90S) 7: 80656687 4.5 9.56 × 10^–8^

*NOS3* rs7792133 G/A (R665H) 7: 151007158 0.3 2.03 × 10^–7^

*ACE* rs4314 C/T (R561W) 17: 63483943 0.3 2.42 × 10^–7^

*BTNL2* rs3806156 G/T 6: 32405921 49.1 2.59 × 10^–7^

*TICRR* rs150565858 G/A (R301Q) 15: 89582933 0.2 4.04 × 10^–7^

*HCG22* rs3873352 G/C 6: 31054336 27.1 4.30 × 10^–7^

*SKIV2L* rs592229 G/T 6: 31962664 42.4 4.58 × 10^–7^

*HCG22* rs2523849 A/G 6: 31057274 28.3 4.63 × 10^–7^

rs2517518 G/A 6: 31060568 28.3 4.63× 10^–7^

*ZNF33B* rs7914982 T/C (H244R) 10: 42593883 0.3 6.05 × 10^–7^

rs9295895 T/C 6: 30470499 27.6 6.15 × 10^–7^

*PPP1R10* rs3895681 G/C 6: 30617994 3.3 6.54 × 10^–7^

*CAT* rs139421991 G/A (R320Q) 11: 34456720 0.3 6.83 × 10^–7^

*TNC* rs138406927 C/T (A1096T) 9: 115064848 2.1 7.60 × 10^–7^

*ABCA1* rs2066714 C/T (M883I) 9: 104824472 36.0 7.63 × 10^–7^

___________________________________________________________________________

The relation of genotypes of SNPs to the serum concentration of HDL-cholesterol was examined by linear regression analysis. *^a^*Major allele/minor allele.
