# Supplementary material for: Identification of eight genetic variants as novel determinants of dyslipidemia in Japanese by exome-wide association studies
Source: Oncotarget. 2017 Apr 17;8(24):38950–61. doi: 10.18632/oncotarget.17159 (PMC5503585; doi:10.18632/oncotarget.17159)
Supplement: Supplementary file 3 [file oncotarget-08-38950-s003.docx]

**Supplementary Table 2.** The 73 SNPs significantly (*P* < 1.21 × 10^–6^) associated with hypertriglyceridemia in the EWAS.

______________________________________________________________________________

Gene dbSNP Nucleotide Chromosome: MAF *P* (allele) Allele

(amino acid) position (%) odds ratio

substitution*^a^*

______________________________________________________________________________

*UTP4* rs193164904 A/G (I534V) 16: 69163131 0.2 6.71 × 10^–149^ 1.34

*ITPK1* rs143953605 C/T (V55I) 14: 93016759 0.9 1.36 × 10^–97^ 0.99

rs499974 G/T 11: 75743976 31.5 6.09 × 10^–85^ 0.97

*SETD7* rs6814310 C/A 4: 139534374 48.4 1.63 × 10^–71^ 0.97

*CGNL1* rs1280396 G/A (A511T) 15: 57439530 12.1 1.43 × 10^–68^ 1.01

*HNRNPF* rs146819332 C/T (V204M) 10: 43387275 1.2 1.42 × 10^–67^ 1.07

*HOXB3* rs200264312 G/A (P284S) 17: 48550780 1.0 8.70 × 10^–67^ 1.00

rs7808146 A/G 7: 124692932 49.8 1.09 × 10^–58^ 1.02

*SNAPC1* rs74810099 T/G (M36R) 14: 61762567 2.8 8.69 × 10^–54^ 0.96

*NRXN3* rs11629205 G/A 14: 78690442 33.3 1.21 × 10^–53^ 0.98

*PLEC* rs201278290 G/A (R1921W) 8: 143924168 0.2 1.92 × 10^–49^ 1.08

*NADSYN1* rs3829251 G/A 11: 71483513 37.5 2.58 × 10^–48^ 1.03

rs10789907 A/C 11: 112689091 35.4 4.64 × 10^–45^ 1.03

*DAW1* rs10191097 T/G 2: 227911955 29.1 4.69 × 10^–44^ 1.03

rs4660080 A/G 1: 241379493 45.8 9.65 × 10^–44^ 0.97

rs10952789 C/A 7: 149041846 36.0 1.13 × 10^–43^ 0.99

*MLH3* rs175080 G/A (P844L) 14: 75047125 16.9 9.05 × 10^–43^ 1.02

*COL6A3* rs36117715 G/A (P2218L) 2: 237353378 0.3 7.29 × 10^–42^ 0.87

rs1462978 A/G 12: 88811065 12.1 1.14 × 10^–37^ 0.96

*SCN10A* rs6795970 G/A (A1073V) 3: 38725184 14.7 4.84 × 10^–34^ 1.00

*SYTL2* rs550404 T/C 11: 85724687 42.0 8.77 × 10^–34^ 0.97

*PTPRD* rs2475335 T/C 9: 10260263 23.0 3.36 × 10^–32^ 1.00

*BUD13* rs10790162 G/A 11: 116768388 26.3 1.69 × 10^–29^ 1.38

rs7350481 C/T 11: 116715567 27.7 1.97 × 10^–29^ 1.37

*FILIP1L* rs182417021 C/T (E236K) 3: 99850250 0.1 5.31 × 10^–29^ 0.50

*KIFAP3* rs1541160 A/G 1: 170026661 0.2 6.54 × 10^–29^ 0.89

*ADGRA3* rs117922332 T/G (K852N) 4: 22392616 7.7 3.97 × 10^–28^ 0.97

*ZMYND8* rs3827047 C/A (E390D) 20: 47276549 11.1 2.16 × 10^–27^ 0.95

*MIS18BP1* rs145716748 A/G (S729P) 14: 45224402 1.9 2.38 × 10^–27^ 1.00

*TCEB3B* rs2010834 A/C (F254C) 18: 47034504 24.5 1.94 × 10^–26^ 0.99

rs12898111 A/G 14: 101202269 42.4 4.37 × 10^–25^ 0.99

*TRIM45* rs1289658 A/G (M496T) 1: 117113466 17.5 5.13 × 10^–21^ 1.09

*SLC7A8* rs2236133 A/G 14: 23159618 29.9 1.46 × 10^–20^ 0.99

*CARD9* rs10781500 C/T 9: 136374886 33.0 5.83 × 10^–20^ 0.99

*LIPT2* rs586088 A/T (T190S) 11: 74492263 31.5 7.27 × 10^–20^ 1.01

*VPS13D* rs143833298 G/A (R830Q) 1: 12276077 0.8 2.78 × 10^–18^ 1.04

rs11180311 A/G 12: 74966448 38.1 8.24 × 10^–18^ 0.98

*ARHGEF19* rs200330080 C/T (R654Q) 1: 16202521 1.7 3.42 × 10^–16^ 1.04

*HEATR5B* rs147241730 T/C (N1383S) 2: 37013977 0.1 4.48 × 10^–16^ 1.83

*ISM1* rs75146235 C/G (Q126E) 20: 13270741 1.4 6.80 × 10^–16^ 0.96

*PLPP7* rs2966332 T/C (M174T) 9: 131307992 28.6 1.49 × 10^–15^ 0.92

*USP47* rs138329346 C/T (H313Y) 11: 11920417 2.4 9.53 × 10^–15^ 0.91

*SLAMF7* rs117009784 A/C (R96S) 1: 160750053 5.8 2.18 × 10^–14^ 1.04

rs11645831 G/A 16: 60207120 42.7 1.38 × 10^–13^ 1.00

*TSLP* rs3806932 A/G 5: 111069977 30.2 4.26 × 10^–13^ 1.02

*LRRC38* rs3013105 C/T (E292K) 1: 13475857 42.0 3.71 × 10^–12^ 0.98

*OAS3* rs35508906 G/A (A584T) 12: 112961163 0.5 1.19 × 10^–11^ 0.74

*MUC5B* rs2672785 G/A (G34E) 11: 1225711 46.4 1.46 × 10^–11^ 1.00

*MTPAP* rs1047991 G/A (R162C) 10: 30340297 24.7 3.50 × 10^–11^ 0.98

*KLHDC9* rs138686208 G/A (E35K) 1: 161098638 0.4 8.75 × 10^–11^ 0.90

rs4331426 A/G 18: 22610832 2.7 1.43 × 10^–10^ 0.97

rs1585440 C/A 13: 65907683 28.6 1.61 × 10^–10^ 1.01

*LDLRAD3* rs200117745 G/C (E16D) 11: 36036104 0.4 9.13 × 10^–10^ 1.26

*PDZD2* rs4867100 C/T 5: 32007607 15.5 1.13 × 10^–9^ 1.06

rs12126589 A/G 1: 104905357 32.1 1.19 × 10^–9^ 1.00

*PKD1L1* rs10951936 A/T 7: 47882084 21.0 1.84 × 10^–9^ 1.03

rs1959607 T/C 14: 19984301 1.5 3.12× 10^–9^ 1.00

C6orf10 rs11751697 C/T 6: 32298648 21.8 6.33 × 10^–9^ 1.06

*MAPK4* rs3752087 G/A (V38M) 18: 50664070 44.7 8.30 × 10^–9^ 1.01

rs991258 G/C 3: 102055199 31.3 1.03 × 10^–8^ 0.99

*TRMT61A* rs200587171 C/T 14: 103534551 0.9 1.96 × 10^–8^ 0.71

*MUC4* rs2246901 A/C (S4821A) 3: 195762138 18.1 2.03 × 10^–8^ 0.98

*TNK1* rs6503018 A/G (M598V) 17: 7388788 29.8 1.43 × 10^–7^ 1.04

*SIM1* rs143803280 G/A 6: 100390370 0.2 2.33 × 10^–7^ 1.03

*CRELD2* rs11545763 A/G (E297G) 22: 49925522 5.4 2.37 × 10^–7^ 1.01

*AKNAD1* rs1277207 T/C (N61S) 1: 108852483 8.9 2.60 × 10^–7^ 1.02

*MAP2K5* rs4489954 T/G 15: 67779737 26.9 2.73× 10^–7^ 0.97

rs7011881 C/A 8: 114201427 17.2 3.21 × 10^–7^ 0.95

*RSPO3* rs1892172 G/A 6: 127155371 48.1 5.54 × 10^–7^ 1.03

*OR5V1* rs1592404 C/G 6: 29390899 19.9 5.70 × 10^–7^ 1.06

rs3130171 T/C 6: 33030952 25.3 5.97 × 10^–7^ 0.98

*MYO16* rs144509002 A/G (D336G) 13: 108823254 3.6 6.17 × 10^–7^ 0.92

*SPC24* rs74491133 C/T 19: 11147894 1.3 7.45 × 10^–7^ 0.85

______________________________________________________________________________

Allele frequencies were analyzed with Fisher’s exact test. *^a^*Major allele/minor allele.
