# Supplementary material for: Identification of eight genetic variants as novel determinants of dyslipidemia in Japanese by exome-wide association studies
Source: Oncotarget. 2017 Apr 17;8(24):38950–61. doi: 10.18632/oncotarget.17159 (PMC5503585; doi:10.18632/oncotarget.17159)
Supplement: Supplementary file 4 [file oncotarget-08-38950-s004.docx]

**Supplementary Table 3.** The 87 SNPs significantly (*P* < 1.21 × 10^–6^) associated with hypo–HDL-cholesterolemia in the EWAS.

______________________________________________________________________________

Gene dbSNP Nucleotide Chromosome: MAF *P* (allele) Allele

(amino acid) position (%) odds ratio

substitution*^a^*

______________________________________________________________________________

*EGFLAM* rs2561111 G/A (R111H) 5: 38350541 14.3 9.77 × 10^–192^ 0.98

*TBC1D17* rs3745486 T/C (L99P) 19: 49880379 40.5 1.12 × 10^–158^ 1.01

*SCLT1* rs77885682 C/T (R503K) 4: 128943120 4.8 3.17 × 10^–158^ 0.95

*N4BP2* rs61748749 T/G (S1353R) 4: 40122170 2.3 7.58 × 10^–144^ 1.00

*LRP2* rs143822500 T/C (T867A) 2: 169257164 0.7 3.46 × 10^–138^ 0.70

rs857591 G/T 1: 186288386 39.7 1.93 × 10^–112^ 1.02

rs499974 G/T 11: 75743976 31.5 7.61 × 10^–105^ 1.04

*HELZ* rs184499441 C/T (G1288R) 17: 67114380 1.6 6.57 × 10^–100^ 1.01

*TTC3* rs1053966 C/G (H1751D) 21: 37195708 17.8 9.60 × 10^–97^ 1.01

*SCN10A* rs12632942 A/G (L1092P) 3: 38723507 46.8 2.43 × 10^–95^ 1.06

*HMHA1* rs150294461 G/A (G654E) 19: 1080682 1.4 1.57 × 10^–94^ 0.94

*ZC3H3* rs3750208 G/A (R168W) 8: 143538865 11.3 5.19 × 10^–84^ 1.03

*MATN2* rs2255317 C/T (T855M) 8: 98032300 15.0 1.62 × 10^–80^ 1.02

*MIS18BP1* rs145716748 A/G (S729P) 14: 45224402 1.9 3.73 × 10^–75^ 1.05

*DAW1* rs10191097 T/G 2: 227911955 29.1 2.24 × 10^–73^ 1.02

*LRFN3* rs148320716 C/T (R96C) 19: 35939711 2.7 2.70 × 10^–73^ 1.12

*SYCP2L* rs2153157 T/C 6: 10897255 30.5 2.96 × 10^–73^ 0.96

rs7299095 G/A 12: 118458499 40.3 3.42 × 10^–71^ 0.95

*CFAP65* rs17852959 C/T (V161M) 2: 219035346 16.7 8.05 × 10^–68^ 1.02

*NRXN3* rs11629205 G/A 14: 78690442 33.3 9.56 × 10^–67^ 1.01

*LIFR* rs3729740 C/T (D578N) 5: 38496535 24.0 5.13 × 10^–58^ 1.05

*OR6T1* rs150534954 C/G (C155W) 11: 123943374 1.4 2.78 × 10^–57^ 1.01

rs11180311 A/G 12: 74966448 38.1 8.79 × 10^–46^ 0.98

*SLC14A1* rs11877062 T/C (W4R) 18: 45727281 46.3 4.78 × 10^–45^ 0.99

*EFCAB6* rs3747203 T/C (R199G) 22: 43735906 19.9 4.95 × 10^–39^ 0.98

*PPP1R32* rs78002652 G/A (G139R) 11: 61484721 0.3 5.81 × 10^–39^ 0.93

*UBE2J1* rs151000241 T/C (N190S) 6: 89333195 0.2 9.78 × 10^–38^ 0.85

*FAT4* rs6847454 A/T (Q453L) 4: 125317769 25.5 3.22 × 10^–35^ 0.97

*VPS13D* rs143833298 G/A (R830Q) 1: 12276077 0.8 5.33 × 10^–30^ 1.17

*GNG8* rs200295807 G/A (R26C) 19: 46634607 0.3 3.75 × 10^–28^ 0.87

*GLI3* rs199606102 C/T (V695I) 7: 41972357 0.7 1.54 × 10^–27^ 0.94

*TSNARE1* rs10100935 C/T (A118T) 8: 142344359 35.5 3.41 × 10^–27^ 0.98

*LIPT2* rs586088 A/T (T190S) 11: 74492263 31.5 2.59 × 10^–25^ 1.06

*USP4* rs146515657 T/C (N650S) 3: 49292533 0.5 1.02 × 10^–24^ 32.89

*ABHD17A* rs4807160 C/T (E144K) 19: 1880951 32.4 5.89 × 10^–24^ 0.98

*TUBGCP6* rs4838865 G/A (S567L) 22: 50226183 17.2 5.56 × 10^–23^ 1.04

*CACNA1D* rs17053501 C/T 3: 53770462 0.4 8.30 × 10^–23^ 1.08

*OR4C15* rs146600946 G/A (R286H) 11: 55555163 2.0 8.33 × 10^–23^ 0.89

*SPTB* rs143827332 G/A (R1035W) 14: 64786862 0.6 1.14 × 10^–22^ 1.10

*PDE4D* rs1823068 A/G 5: 59380223 21.8 2.77 × 10^–21^ 0.99

*SETDB2* rs7997737 G/A 13: 49459052 44.8 3.17 × 10^–20^ 0.99

rs9901755 A/G 17: 65604247 45.8 2.54 × 10^–19^ 0.97

*ATXN7L1* rs150412190 G/A (S116L) 7: 105788612 0.3 7.85 × 10^–19^ 1.13

*OPLAH* rs7004867 C/T (R31Q) 8: 144059941 0.3 1.12 × 10^–17^ 1.33

*MAP2* rs2271251 C/G (A82G) 2: 209653415 0.7 1.94 × 10^–17^ 1.03

*ZNF225* rs62623665 G/A (R352H) 19: 44131669 0.2 1.07 × 10^–16^ 1.37

*ANKZF1* rs57075420 C/T (R297C) 2: 219233784 1.8 1.92 × 10^–15^ 1.07

rs7442317 G/A 4: 29901430 38.6 2.93 × 10^–15^ 1.06

*OR10R2* rs3820678 G/A (A191T) 1: 158480448 20.9 4.91 × 10^–15^ 0.98

*ZNF683* rs10794531 C/T (R53H) 1: 26367754 37.7 7.30 × 10^–15^ 1.01

*POLI* rs78943519 G/A (G15D) 18: 54269590 26.2 1.79 × 10^–14^ 0.99

*OR4D5* rs149721746 A/G (Y60C) 11: 123939795 0.9 2.18 × 10^–14^ 0.71

rs7619670 G/A 3: 31344740 19.1 2.72 × 10^–14^ 0.98

*CD163L1* rs199763816 G/A (P1116S) 12: 7374505 0.1 6.34 × 10^–14^ 0.58

*HMCN1* rs1555494 A/G 1: 185917019 0.1 1.15 × 10^–13^ 0.69

*GBAP1* rs2049805 C/T 1: 155225189 17.5 1.28× 10^–13^ 1.01

rs11247229 C/T 15: 100654973 33.9 1.32 × 10^–13^ 0.96

rs6598858 C/T 1: 26583274 19.8 2.23 × 10^–13^ 1.09

*TRABD2B* rs147317864 C/T (A262T) 1: 47801502 0.2 2.37 × 10^–13^ ND

*DBR1* rs118174683 G/A (T482M) 3: 138162079 0.1 3.89 × 10^–13^ 1.04

*ZNF169* rs1536690 C/T (P72L) 9: 94293028 8.5 6.45 × 10^–13^ 1.00

rs17316633 G/A 4: 109904478 9.8 6.66 × 10^–13^ 0.96

*CPNE9* rs139476663 T/C (V87A) 3: 9704994 0.4 2.38 × 10^–12^ 1.04

rs8059612 G/A 16: 59471994 39.8 2.54 × 10^–12^ 1.03

LOC101929380 rs2032794 A/G 5: 87136800 22.8 9.44 × 10^–12^ 0.98

*FSIP2* rs992822 G/A (S910N) 2: 185789865 47.2 1.11 × 10^–11^ 1.06

*TMPO* rs17028450 C/T (R690C) 12: 98534325 1.1 3.29 × 10^–10^ 1.05

rs1980889 A/G 9: 89284330 37.5 7.14 × 10^–10^ 0.99

*ZC3HC1* rs1464890 C/T (A271T) 7: 130024472 27.4 8.13 × 10^–10^ 0.98

rs2324027 C/T 17: 15776287 48.5 1.18 × 10^–9^ 1.01

rs7667636 G/A 4: 134820637 49.6 1.57 × 10^–9^ 0.97

*PHIP* rs10943613 T/C 6: 79035390 35.0 2.25 × 10^–9^ 1.02

*MYO7A* rs948962 C/A (L1954I) 11: 77208433 29.9 2.50 × 10^–9^ 1.05

*CEP126* rs76022391 G/A (G668S) 11: 101963037 2.8 4.65 × 10^–9^ 0.85

rs7913069 C/T 10: 103954641 7.6 7.22 × 10^–9^ 1.09

*ARHGEF19* rs200330080 C/T (R654Q) 1: 16202521 1.7 7.38 × 10^–9^ 0.85

*MYO7B* rs111765932 T/C (I926T) 2: 127608841 0.2 8.81 × 10^–9^ 0.78

*GSN* rs141510612 G/A (R690Q) 9: 121332476 0.1 1.64 × 10^–8^ 1.12

*AP3S1* rs74844425 T/C (I15V) 5: 115841510 0.7 1.96 × 10^–8^ 0.88

rs12229654 T/G 12: 110976657 22.5 4.46 × 10^–8^ 1.22

*PLEKHA4* rs145141779 G/A (P337L) 19: 48857459 3.2 6.67 × 10^–8^ 0.92

*KCNN2* rs13188074 A/G 5: 114465726 31.2 6.98 × 10^–8^ 1.04

*PIK3R4* rs56369596 G/C (V699L) 3: 130718421 2.6 1.26 × 10^–7^ 0.92

*FSTL1* rs874478 G/A 3: 120439364 25.0 1.77 × 10^–7^ 1.05

*ELFN2* rs202021460 C/T (A252T) 22: 37374781 0.1 2.45 × 10^–7^ 1.50

rs179075 T/C 14: 30322957 33.7 3.88 × 10^–7^ 1.00

*DLGAP3* rs6699355 C/T 1: 34919004 32.8 5.05 × 10^–7^ 1.00

______________________________________________________________________________

Allele frequencies were analyzed with Fisher’s exact test. *^a^*Major allele/minor allele. ND, not determined.
