# Supplementary material for: Identification of eight genetic variants as novel determinants of dyslipidemia in Japanese by exome-wide association studies
Source: Oncotarget. 2017 Apr 17;8(24):38950–61. doi: 10.18632/oncotarget.17159 (PMC5503585; doi:10.18632/oncotarget.17159)
Supplement: Supplementary file 5 [file oncotarget-08-38950-s005.docx]

**Supplementary Table 4.** The 114 SNPs significantly (*P* < 1.21 × 10^–6^) associated with hyper–LDL-cholesterolemia in the EWAS.

______________________________________________________________________________

Gene dbSNP Nucleotide Chromosome: MAF *P* (allele) Allele

(amino acid) position (%) odds ratio

substitution*^a^*

______________________________________________________________________________

rs2125904 C/A 2: 106582619 26.8 2.02 × 10^–39^ 1.02

*ARHGAP27* rs2959953 C/G (P213R) 17: 45429642 48.6 1.28 × 10^–21^ 1.06

*TTC22* rs12144325 A/G (L366P) 1: 54782401 2.7 1.12 × 10^–17^ 1.00

*FREM2* rs2496425 T/C (F1070S) 13: 38690553 38.3 6.82 × 10^–17^ 1.03

rs10097386 T/C 8: 92633008 44.7 3.00 × 10^–16^ 1.02

*OR4X2* rs7120775 C/G (Y27*) 11: 48245184 16.9 6.94 × 10^–16^ 0.99

*UBXN11* rs138559558 G/A (R289C) 1: 26284470 1.2 1.47 × 10^–15^ 0.92

rs7532317 C/A 1: 104193534 37.7 1.88 × 10^–14^ 1.01

*AKT3* rs4132509 C/A 1: 243779782 27.8 1.88 × 10^–14^ 0.99

*ACSM3* rs5716 G/C (K367N) 16: 20785065 17.3 2.51 × 10^–14^ 1.02

rs7771335 A/G 6: 29480261 22.3 2.57 × 10^–14^ 1.26

*COL18A1* rs79980197 C/G (P706R) 21: 45491274 2.7 9.86 × 10^–14^ 1.16

*KMT2D* rs201078160 C/T (R4162Q) 12: 49032220 0.1 1.61 × 10^–13^ 1.79

*HRG* rs10770 T/C (I180T) 3: 186671770 9.5 2.68 × 10^–13^ 0.97

*C21orf59* rs76974938 C/T (D67N) 21: 32609946 2.4 4.70 × 10^–13^ 0.49

*UBR7* rs76828246 G/A (S277N) 14: 93219231 0.2 5.58 × 10^–13^ 0.71

*MOG* rs2071653 C/T 6: 29668151 26.7 4.05 × 10^–12^ 1.22

*C12orf42* rs10778257 T/G (E11D) 12: 103478394 49.9 6.48 × 10^–12^ 1.02

*PITRM1* rs6901 C/T (R1038Q) 10: 3138035 23.8 8.70 × 10^–12^ 1.05

*PTPN13* rs200400344 G/A (R366Q) 4: 86701703 0.4 9.70 × 10^–12^ 1.24

*WBSCR17* rs7793970 G/A 7: 71271182 49.3 1.09 × 10^–11^ 0.98

*PEAK1* rs56133554 T/G (T1077P) 15: 77158605 3.6 1.34 × 10^–11^ 1.04

*ATXN7L1* rs150412190 G/A (S116L) 7: 105788612 0.3 1.38 × 10^–11^ 1.07

*SEC31A* rs3797036 A/C (N456K) 4: 82864428 2.1 2.79 × 10^–11^ 0.88

rs60312980 G/T 5: 92504930 3.6 4.09 × 10^–11^ 1.14

*DCAF4L2* rs146243553 A/G (S67P) 8: 87873773 0.5 5.59 × 10^–11^ 1.03

*TNC* rs1757106 T/G 9: 115083054 41.8 6.19 × 10^–11^ 1.08

*ZDHHC5* rs117135042 C/T (S634L) 11: 57699337 3.1 7.34 × 10^–11^ 1.04

*TUBD1* rs1292053 G/A (T76M) 17: 59886176 40.8 1.16 × 10^–10^ 0.98

*PIGB* rs151252589 T/G (F327L) 15: 55340746 1.3 1.25 × 10^–10^ 1.03

*OGDHL* rs11101224 G/A (T428M) 10: 49742930 7.8 1.26 × 10^–10^ 1.02

rs2853969 C/T 6: 31388797 9.7 1.26 × 10^–10^ 1.31

*TTC3* rs2835655 G/A 21: 37191424 48.3 1.50 × 10^–10^ 0.98

*MICAL3* rs202169174 T/C (D1528G) 22: 17818078 0.1 1.67 × 10^–10^ 0.94

*AIM1L* rs151324745 A/C (Y1136D) 1: 26338416 1.1 2.09 × 10^–10^ 0.99

*LEMD2* rs2395402 T/C 6: 33785896 18.2 2.17 × 10^–10^ 1.11

*CPEB1* rs783540 G/A 15: 82585958 49.8 2.17 × 10^–10^ 1.02

*CCT5* rs147989324 G/A (V304I) 5: 10261641 0.2 2.37 × 10^–10^ 1.01

*WBSCR27* rs11543598 G/A (P213L) 7: 73834843 1.7 2.66 × 10^–10^ 1.09

*PTPRJ* rs7124275 T/C 11: 48140753 45.4 2.77 × 10^–10^ 1.01

*PVRL3* rs6801425 A/G 3: 111222015 39.3 4.04 × 10^–10^ 1.04

*PSEN2* rs200636353 G/A (G34S) 1: 226882007 0.3 6.58 × 10^–10^ 1.34

*KCNJ15* rs3746875 A/C (M30L) 21: 38299349 0.9 7.06 × 10^–10^ 1.01

*SLC7A9* rs1007160 G/T (L223M) 19: 32862155 30.9 7.95 × 10^–10^ 1.01

OR9K2 rs7305779 A/C (E103A) 12: 55130076 9.8 9.11 × 10^–10^ 1.02

*FANCA* rs17232910 G/C (A643P) 16: 89773358 10.0 1.81 × 10^–9^ 1.06

*C14orf105* rs1152522 C/T 14: 57481662 4.4 2.02 × 10^–9^ 0.91

*OSCP1* rs61308377 A/G (Y209H) 1: 36428367 21.4 2.63 × 10^–9^ 0.97

*WDR19* rs144335584 A/G (I747V) 4: 39232258 1.0 3.27 × 10^–9^ 0.81

*SLC2A9* rs3775948 G/C 4: 9993558 42.4 6.22 × 10^–9^ 0.96

*PRSS37* rs12669721 G/T (P119T) 7: 141837935 21.7 6.90 × 10^–9^ 1.00

*TULP3* rs3944066 C/T (P494L) 12: 2940701 1.0 7.00 × 10^–9^ 1.03

*SP1* rs144134358 C/T (S569L) 12: 53409367 0.1 7.94 × 10^–9^ 0.52

*SEC14L5* rs199905767 C/A (P620T) 16: 5011152 0.1 8.87 × 10^–9^ 0.67

*IFIT2* rs41284134 G/C (G245R) 10: 89306689 1.1 1.10 × 10^–8^ 1.13

*SHANK1* rs201453898 C/T (R547Q) 19: 50702574 0.2 1.33 × 10^–8^ 0.81

*PPP1R18* rs2269704 C/T 6: 30689176 13.1 1.33 × 10^–8^ 1.24

*SERAC1* rs115387731 C/T (V8I) 6: 158158342 0.2 1.44 × 10^–8^ 0.74

*NRM* rs2269703 G/A 6: 30690608 13.1 1.61 × 10^–8^ 1.23

*DDR1* rs1264318 G/C 6: 30895753 5.9 1.99 × 10^–8^ 0.96

*TMCO4* rs10917536 G/T (Q72K) 1: 19771448 37.4 2.21 × 10^–8^ 1.01

*IL22RA1* rs148768286 C/T (V266I) 1: 24121734 0.6 2.23 × 10^–8^ 0.89

rs7442317 G/A 4: 29901430 38.6 2.66 × 10^–8^ 1.04

*FBXO42* rs12069239 G/C (A471P) 1: 16251413 15.7 2.85 × 10^–8^ 1.03

*LY6G6D* rs9469042 T/C 6: 31715241 6.8 2.86 × 10^–8^ 0.94

rs618662 C/A 18: 43392927 29.8 2.98 × 10^–8^ 1.02

rs8133766 T/C 21: 24571645 40.3 3.08× 10^–8^ 1.02

*SUOX* rs117778870 G/A (R40H) 12: 56002611 1.7 3.43 × 10^–8^ 0.94

*PADI1* rs140750531 G/A (R551H) 1: 17240654 1.8 3.49 × 10^–8^ 0.85

*NCKAP5* rs4953863 C/T 2: 132915280 49.0 3.78 × 10^–8^ 1.02

*EFCAB5* rs74546291 G/A (W240*) 17: 29993284 0.6 4.46 × 10^–8^ 1.12

*TNIP3* rs10000692 T/C (K99E) 4: 121157162 0.3 4.47 × 10^–8^ 1.14

*ADAL* rs2278857 T/C 15: 43340351 45.7 4.68 × 10^–8^ 1.03

*DNAH11* rs72655988 G/A (A568T) 7: 21582013 0.7 4.69 × 10^–8^ 0.88

*ALDH1L1* rs2276724 T/C (S491G) 3: 126135566 27.5 4.78 × 10^–8^ 1.06

*LY75-CD302* rs1549579 T/G 2: 159812150 37.1 5.14 × 10^–8^ 1.05

rs495089 T/C 6: 32229686 41.2 5.56 × 10^–8^ 1.15

*CUBN* rs1801232 G/T (N3552K) 10: 16828913 8.9 5.87 × 10^–8^ 1.07

*TSACC* rs151057154 G/C (E67Q) 1: 156346803 0.2 6.21 × 10^–8^ 1.04

*FAM180A* rs59178195 T/G (Q150P) 7: 135734048 2.9 6.42 × 10^–8^ 1.12

*OR9G1* rs79060400 T/C 11: 56701218 13.7 6.42 × 10^–8^ 1.02

*CPT1B* rs5770917 T/C 22: 50578924 19.7 6.67 × 10^–8^ 1.03

*GLT6D1* rs138281407 C/G (P130R) 9: 135624539 0.3 7.55 × 10^–8^ 1.18

*AARS* rs2070203 G/A 16: 70269677 48.4 1.05 × 10^–7^ 0.99

*WWP2* rs4275849 G/A 16: 69798202 49.1 1.05 × 10^–7^ 1.05

*SNRNP200* rs3171927 A/G 2: 96287095 27.0 1.06 × 10^–7^ 0.98

*OR1J2* rs112619503 C/T (R165W) 9: 122511294 0.8 1.26 × 10^–7^ 1.36

rs3108919 T/C 8: 100829318 49.1 1.37 × 10^–7^ 0.95

rs1412115 G/A 10: 33799125 48.1 1.38 × 10^–7^ 0.98

*MDC1* rs2269702 A/G 6: 30707358 17.7 1.50 × 10^–7^ 1.19

*TTYH2* rs35999669 T/G (S265A) 17: 74244038 10.7 1.56 × 10^–7^ 1.04

rs4711319 G/A 6: 33139684 30.8 1.63 × 10^–7^ 0.96

*ERAP2* rs2549782 T/G (N392K) 5: 96895296 49.2 1.66 × 10^–7^ 0.92

*WFS1* rs1805070 A/G (I720V) 4: 6301953 8.5 1.76 × 10^–7^ 1.01

*PIK3R5* rs714407 G/A 17: 8957176 41.4 1.80 × 10^–7^ 0.96

*FNDC1* rs192084699 G/T 6: 159238631 0.8 2.17 × 10^–7^ 0.79

*FAM221A* rs35928055 A/G (S240G) 7: 23698272 12.4 2.19 × 10^–7^ 0.93

*ANKIB1* rs2374563 A/G 7: 92345443 46.2 2.23 × 10^–7^ 1.00

*TTN* rs55675869 C/T (V33366I) 2: 178537013 6.0 2.33 × 10^–7^ 0.92

*IBSP* rs1054629 A/T (E270D) 4: 87811766 11.5 2.44 × 10^–7^ 1.00

rs6566532 T/C 18: 71384090 47.1 2.60 × 10^–7^ 0.97

*ADNP2* rs141645766 T/G (S677A) 18: 80137442 0.1 3.19 × 10^–7^ 1.89

rs2523638 G/A 6: 31376496 43.1 3.53 × 10^–7^ 0.97

*FAM208B* rs2254067 G/T (G499C) 10: 5739665 22.4 4.06 × 10^–7^ 0.98

*HPS4* rs146303784 C/T (M649I) 22: 26457867 1.2 4.08 × 10^–7^ 0.96

*TTBK2* rs34348991 G/A (T1084M) 15: 42751995 7.8 4.42 × 10^–7^ 1.03

rs12511469 A/T 4: 154530607 38.2 6.67 × 10^–7^ 1.00

*INADL* rs146406799 C/T (R1717W) 1: 62128077 0.8 6.69 × 10^–7^ 0.85

*TTC7B* rs61742122 G/A 14: 90786306 0.3 6.80 × 10^–7^ 0.61

*TTC5* rs3742945 C/T (R47Q) 14: 20301877 20.5 7.15 × 10^–7^ 0.99

*CCM2* rs2289367 G/A 7: 45073571 16.4 7.59 × 10^–7^ 1.07

*OR4C46* rs77689730 C/T (L211F) 11: 54603368 28.8 7.71 × 10^–7^ 0.98

rs1233399 C/T 6: 29571705 24.3 8.14 × 10^–7^ 0.86

*QRICH2* rs73996306 G/A (A69V) 17: 76304416 8.4 8.41 × 10^–7^ 1.01

______________________________________________________________________________

Allele frequencies were analyzed with Fisher’s exact test. *^a^*Major allele/minor allele.
