# Supplementary material for: Identification of eight genetic variants as novel determinants of dyslipidemia in Japanese by exome-wide association studies
Source: Oncotarget. 2017 Apr 17;8(24):38950–61. doi: 10.18632/oncotarget.17159 (PMC5503585; doi:10.18632/oncotarget.17159)
Supplement: Supplementary file 6 [file oncotarget-08-38950-s006.docx]

**Supplementary Table 5.** Genotype distributions for SNPs associated (*P* < 1.21 × 10^–6^) with hypertriglyceridemia in the EWAS.

____________________________________________________________________________________________________________

SNP Hypertriglyceridemia H-W *P* Controls H-W *P*

____________________________________________________________________________________________________________

rs193164904 A/G (I534V) *AA* *AG* *GG* *AA* *AG* *GG*

4723 (99.60) 19 (0.40) 0 (0) 1.0000 8647 (99.71) 24 (0.28) 1 (0.01) 0.0186

rs143953605 C/T (V55I) *CC* *CT* *TT*  *CC* *CT* *TT*

4659 (98.25) 83 (1.75) 0 (0) 1.0000 8519 (98.24) 152 (1.75) 1 (0.01) 0.4961

rs499974 G/T *GG* *GT* *TT*  *GG* *GT* *TT*

2278 (48.04) 1978 (41.71) 486 (10.25) 0.0673 4041 (46.60) 3743 (43.17) 887 (10.23) 0.6382

rs6814310 C/A *CC* *CA* *AA*  *CC* *CA* *AA*

1286 (27.12) 2348 (49.53) 1107 (23.35) 0.5805 2231 (25.73) 4397 (50.70) 2044 (23.57) 0.1828

rs1280396 G/A (A511T) *GG* *GA* *AA*  *GG* *GA* *AA*

3674 (77.50) 992 (20.92) 75 (1.58) 0.4103 6726 (77.56) 1822 (21.01) 124 (1.43) 0.9593

rs146819332 C/T (V204M) *CC* *CT* *TT*  *CC* *CT* *TT*

4626 (97.55) 116 (2.45) 0 (0) 1.0000 8475 (97.73) 196 (2.26) 1 (0.01) 1.0000

rs200264312 G/A (P284S) *GG* *GA* *AA*  *GG* *GA* *AA*

4655 (98.17) 85 (1.79) 2 (0.04) 0.0635 8510 (98.13) 161 (1.86) 1 (0.01) 0.5363

rs7808146 A/G *AA* *AG* *GG* *AA* *AG* *GG*

1166 (24.59) 2397 (50.55) 1179 (24.86) 0.4678 2216 (25.55) 4297 (49.55) 2159 (24.90) 0.4023

rs74810099 T/G (M36R) *TT* *TG* *GG*  *TT* *TG* *GG*

4493 (94.75) 239 (5.04) 10 (0.21) 0.0025 8187 (94.41) 477 (5.50) 8 (0.09) 0.6944

rs11629205 G/A *GG* *GA* *AA*  *GG* *GA* *AA*

2161 (45.59) 2038 (43.00) 541 (11.41) 0.0705 3822 (44.08) 3911 (45.10) 938 (10.82) 0.1922

rs201278290 G/A (R1921W) *GG* *GA* *AA*  *GG* *GA* *AA*

4728 (99.73) 13 (0.27) 0 (0) 1.0000 8645 (99.75) 22 (0.25) 0 (0) 1.0000

rs3829251 G/A *GG* *GA* *AA*  *GG* *GA* *AA*

1851 (39.04) 2185 (46.09) 705 (14.87) 0.1472 3395 (39.15) 4090 (47.17) 1186 (13.68) 0.4215

rs10789907 A/C *AA* *AC* *CC*  *AA* *AC* *CC*

1961 (41.35) 2165 (45.66) 616 (12.99) 0.6357 3670 (42.32) 3891 (44.87) 1111 (12.81) 0.1150

rs10191097 T/G *TT* *TG* *GG*  *TT* *TG* *GG*

2330 (49.15) 2006 (42.31) 405 (8.54) 0.3845 4377 (50.47) 3551 (40.95) 744 (8.58) 0.5319

rs4660080 A/G *AA* *AG* *GG* *AA* *AG* *GG*

1429 (30.14) 2330 (49.13) 983 (20.73) 0.5579 2539 (29.28) 4281 (49.37) 1852 (21.35) 0.5452

rs10952789 C/A *CC* *CA* *AA*  *CC* *CA* *AA*

1804 (40.90) 2056 (46.61) 551 (12.49) 0.3592 3603 (41.55) 3895 (44.92) 1173 (13.53) 0.0198

rs175080 G/A (P844L) *GG* *GA* *AA*  *GG* *GA* *AA*

3252 (68.58) 1344 (28.34) 146 (3.08) 0.6111 5986 (69.03) 2437 (28.10) 249 (2.87) 0.9695

rs36117715 G/A (P2218L) *GG* *GA* *AA*  *GG* *GA* *AA*

4715 (99.43) 27 (0.57) 0 (0) 1.0000 8614 (99.34) 57 (0.66) 0 (0) 1.0000

rs1462978 A/G *AA* *AG* *GG* *AA* *AG* *GG*

3693 (77.91) 978 (20.63) 69 (1.46) 0.6247 6704 (77.31) 1829 (21.09) 139 (1.60) 0.2683

rs6795970 G/A (A1073V) *GG* *GA* *AA*  *GG* *GA* *AA*

3466 (73.09) 1153 (24.32) 123 (2.59) 0.0242 6308 (72.74) 2163 (24.94) 201 (2.32) 0.3273

rs550404 T/C *TT* *TC* *CC*  *TT* *TC* *CC*

1629 (34.35) 2314 (48.80) 799 (16.85) 0.6532 2940 (33.90) 4189 (48.31) 1543 (17.79) 0.4535

rs2475335 T/C *TT* *TC* *CC*  *TT* *TC* *CC*

2805 (59.18) 1677 (35.38) 258 (5.44) 0.7133 5119 (59.05) 3091 (35.66) 459 (5.29) 0.8090

rs10790162 G/A *GG* *GA* *AA*  *GG* *GA* *AA*

2318 (48.89) 1969 (41.53) 454 (9.58) 0.2292 5057 (58.32) 3072 (35.43) 542 (6.25) 0.0103

rs7350481 C/T *CC* *CT* *TT*  *CC* *CT* *TT*

2174 (45.85) 2095 (44.19) 472 (9.96) 0.3335 4829 (55.69) 3253 (37.51) 590 (6.80) 0.1848

rs182417021 C/T (E236K) *CC* *CT* *TT*  *CC* *CT* *TT*

4736 (99.87) 6 (0.13) 0 (0) 1.0000 8650 (99.75) 22 (0.25) 0 (0) 1.0000

rs1541160 A/G *AA* *AG* *GG* *AA* *AG* *GG*

4723 (99.60) 19 (0.40) 0 (0) 1.0000 8633 (99.56) 37 (0.43) 1 (0.01) 0.0419

rs117922332 T/G (K852N) *TT* *TG* *GG*  *TT* *TG* *GG*

4073 (85.89) 643 (13.56) 26 (0.55) 0.9147 7396 (85.28) 1239 (14.29) 37 (0.43) 0.0546

rs3827047 C/A (E390D) *CC* *CA* *AA*  *CC* *CA* *AA*

3784 (79.82) 901 (19.00) 56 (1.18) 0.7608 6829 (78.76) 1736 (20.02) 106 (1.22) 0.7468

rs145716748 A/G (S729P) *AA* *AG* *GG* *AA* *AG* *GG*

4570 (96.37) 172 (3.63) 0 (0) 0.4080 8362 (96.42) 305 (3.52) 5 (0.06) 0.2118

rs2010834 A/C (F254C) *AA* *AC* *CC*  *AA* *AC* *CC*

2705 (57.04) 1751 (36.93) 286 (6.03) 0.9063 4931 (56.87) 3215 (37.08) 525 (6.05) 0.9769

rs12898111 A/G *AA* *AG* *GG* *AA* *AG* *GG*

1587 (33.47) 2296 (48.43) 858 (18.10) 0.5923 2848 (32.84) 4259 (49.11) 1565 (18.05) 0.7089

rs1289658 A/G (M496T) *AA* *AG* *GG* *AA* *AG* *GG*

3156 (66.60) 1436 (30.30) 147 (3.10) 0.3067 5967 (68.84) 2446 (28.22) 255 (2.94) 0.8198

rs2236133 A/G *AA* *AG* *GG* *AA* *AG* *GG*

2344 (49.44) 1983 (41.43) 414 (8.73) 0.8892 4293 (49.50) 3565 (41.11) 814 (9.39) 0.0617

rs10781500 C/T *CC* *CT* *TT*  *CC* *CT* *TT*

2169 (45.74) 2059 (43.42) 514 (10.84) 0.4469 3950 (45.55) 3768 (43.46) 953 (10.99) 0.2320

rs586088 A/T (T190S) *AA* *AT* *TT*  *AA* *AT* *TT*

2218 (46.79) 2029 (42.81) 493 (10.40) 0.3660 4078 (47.03) 3723 (42.94) 870 (10.03) 0.6364

rs143833298 G/A (R830Q) *GG* *GA* *AA*  *GG* *GA* *AA*

4661 (98.29) 81 (1.71) 0 (0) 1.0000 8530 (98.36) 141 (1.63) 1 (0.01) 0.4458

rs11180311 A/G *AA* *AG* *GG* *AA* *AG* *GG*

1854 (39.11) 2215 (46.72) 672 (14.17) 0.8043 3323 (38.32) 4096 (47.23) 1253 (14.45) 0.8913

rs200330080 C/T (R654Q) *CC* *CT* *TT*  *CC* *CT* *TT*

4582 (96.63) 157 (3.31) 3 (0.06) 0.1620 8386 (96.71) 283 (3.27) 2 (0.02) 1.0000

rs147241730 T/C (N1383S) *TT* *TC* *CC*  *TT* *TC* *CC*

4719 (99.60) 19 (0.40) 0 (0) 1.0000 8649 (99.78) 19 (0.22) 0 (0) 1.0000

rs75146235 C/G (Q126E) *CC* *CG* *GG*  *CC* *CG* *GG*

4603 (97.09) 138 (2.91) 0 (0) 0.6267 8411 (96.99) 259 (2.99) 2 (0.02) 1.0000

rs2966332 T/C (M174T) *TT* *TC* *CC*  *TT* *TC* *CC*

2525 (53.25) 1857 (39.16) 360 (7.59) 0.4632 4385 (50.57) 3585 (41.34) 702 (8.09) 0.4318

rs138329346 C/T (H313Y) *CC* *CT* *TT*  *CC* *CT* *TT*

4539 (95.72) 198 (4.18) 5 (0.10) 0.0759 8259 (95.24) 410 (4.73) 3 (0.03) 0.4923

rs117009784 A/C (R96S) *AA* *AC* *CC*  *AA* *AC* *CC*

4198 (88.53) 528 (11.13) 16 (0.34) 1.0000 7716 (88.97) 925 (10.67) 31 (0.36) 0.5477

rs11645831 G/A *GG* *GA* *AA*  *GG* *GA* *AA*

1565 (33.00) 2321 (48.95) 856 (18.05) 0.9526 2852 (32.89) 4255 (49.06) 1565 (18.05) 0.7585

rs3806932 A/G *AA* *AG* *GG* *AA* *AG* *GG*

2300 (48.50) 1972 (41.59) 470 (9.91) 0.1166 4237 (48.86) 3623 (41.78) 812 (9.36) 0.3596

rs3013105 C/T (E292K) *CC* *CT* *TT*  *CC* *CT* *TT*

1623 (34.23) 2284 (48.18) 834 (17.59) 0.5306 2910 (33.56) 4222 (48.69) 1539 (17.75) 0.9123

rs35508906 G/A (A584T) *GG* *GA* *AA*  *GG* *GA* *AA*

4702 (99.30) 33 (0.70) 0 (0) 1.0000 8585 (99.05) 82 (0.95) 0 (0) 1.0000

rs2672785 G/A (G34E) *GG* *GA* *AA*  *GG* *GA* *AA*

1336 (28.19) 2411 (50.86) 993 (20.95) 0.1218 2492 (28.74) 4300 (49.58) 1880 (21.68) 0.7625

rs1047991 G/A (R162C) *GG* *GA* *AA*  *GG* *GA* *AA*

2722 (57.41) 1740 (36.70) 279 (5.89) 0.9685 4949 (57.07) 3168 (36.54) 554 (6.39) 0.1255

rs138686208 G/A (E35K) *GG* *GA* *AA*  *GG* *GA* *AA*

4708 (99.28) 34 (0.72) 0 (0) 1.0000 8603 (99.20) 69 (0.80) 0 (0) 1.0000

rs4331426 A/G *AA* *AG* *GG* *AA* *AG* *GG*

4498 (94.86) 242 (5.10) 2 (0.04) 0.7716 8219 (94.79) 441 (5.08) 11 (0.13) 0.0585

rs1585440 C/A *CC* *CA* *AA*  *CC* *CA* *AA*

2418 (51.07) 1926 (40.67) 391 (8.26) 0.7760 4477 (51.63) 3452 (39.80) 743 (8.57) 0.0349

rs200117745 G/C (E16D) *GG* *GC* *CC*  *GG* *GC* *CC*

4693 (99.01) 46 (0.97) 1 (0.02) 0.1127 8603 (99.21) 68 (0.78) 1 (0.01) 0.1305

rs4867100 C/T *CC* *CT* *TT*  *CC* *CT* *TT*

3346 (70.57) 1270 (26.79) 125 (2.64) 0.7461 6227 (71.82) 2237 (25.80) 206 (2.38) 0.7715

rs12126589 A/G *AA* *AG* *GG* *AA* *AG* *GG*

2197 (46.33) 2051 (43.25) 494 (10.42) 0.6407 4026 (46.43) 3723 (42.93) 923 (10.64) 0.1532

rs10951936 A/T *AA* *AT* *TT*  *AA* *AT* *TT*

2926 (61.72) 1602 (33.79) 213 (4.49) 0.7625 5430 (62.62) 2852 (32.89) 389 (4.49) 0.5592

rs1959607 T/C *TT* *TC* *CC*  *TT* *TC* *CC*

4595 (96.92) 145 (3.06) 1 (0.02) 1.0000 8404 (96.92) 264 (3.05) 3 (0.03) 0.4683

rs11751697 C/T *CC* *CT* *TT*  *CC* *CT* *TT*

2822 (59.55) 1670 (35.24) 247 (5.21) 1.0000 5322 (61.38) 2915 (33.62) 434 (5.00) 0.1759

rs3752087 G/A (V38M) *GG* *GA* *AA*  *GG* *GA* *AA*

1451 (30.63) 2339 (49.38) 947 (19.99) 0.9531 2709 (31.25) 4238 (48.88) 1723 (19.87) 0.3724

rs991258 G/C *GG* *GC* *CC*  *GG* *GC* *CC*

2249 (47.43) 2039 (43.00) 454 (9.57) 0.8124 4112 (47.42) 3689 (42.54) 871 (10.04) 0.3061

rs200587171 C/T *CC* *CT* *TT*  *CC* *CT* *TT*

4661 (98.56) 68 (1.44) 0 (0) 1.0000 8458 (97.97) 175 (2.03) 0 (0) 1.0000

rs2246901 A/C (S4821A) *AA* *AC* *CC*  *AA* *AC* *CC*

3205 (67.59) 1391 (29.33) 146 (3.08) 0.7655 5833 (67.26) 2547 (29.37) 292 (3.37) 0.4905

rs6503018 A/G (M598V) *AA* *AG* *GG* *AA* *AG* *GG*

2334 (49.22) 1933 (40.76) 475 (10.02) 0.0122 4281 (49.37) 3650 (42.09) 741 (8.54) 0.3536

rs143803280 G/A *GG* *GA* *AA*  *GG* *GA* *AA*

4719 (99.52) 23 (0.48) 0 (0) 1.0000 8631 (99.53) 41 (0.47) 0 (0) 1.0000

rs11545763 A/G (E297G) *AA* *AG* *GG* *AA* *AG* *GG*

4243 (89.48) 483 (10.18) 16 (0.34) 0.5699 7764 (89.54) 881 (10.16) 26 (0.30) 0.8326

rs1277207 T/C (N61S) *TT* *TC* *CC*  *TT* *TC* *CC*

3940 (83.10) 758 (15.99) 43 (0.91) 0.3243 7226 (83.33) 1375 (15.85) 71 (0.82) 0.5445

rs4489954 T/G *TT* *TG* *GG*  *TT* *TG* *GG*

2573 (54.27) 1841 (38.83) 327 (6.90) 0.9403 4649 (53.61) 3383 (39.01) 640 (7.38) 0.4776

rs7011881 C/A *CC* *CA* *AA*  *CC* *CA* *AA*

3284 (69.25) 1309 (27.61) 149 (3.14) 0.1801 5871 (67.70) 2549 (29.39) 252 (2.91) 0.2220

rs1892172 G/A *GG* *GA* *AA*  *GG* *GA* *AA*

1259 (26.55) 2361 (49.79) 1122 (23.66) 0.8162 2375 (27.39) 4318 (49.79) 1979 (22.82) 0.8464

rs1592404 C/G *CC* *CG* *GG*  *CC* *CG* *GG*

3023 (63.75) 1502 (31.67) 217 (4.58) 0.0893 5649 (65.14) 2673 (30.82) 350 (4.04) 0.1316

rs3130171 T/C *TT* *TC* *CC*  *TT* *TC* *CC*

2666 (56.22) 1774 (37.41) 302 (6.37) 0.7573 4825 (55.64) 3285 (37.88) 562 (6.48) 0.9323

rs144509002 A/G (D336G) *AA* *AG* *GG* *AA* *AG* *GG*

4426 (93.37) 308 (6.50) 6 (0.13) 0.6588 8055 (92.91) 594 (6.85) 21 (0.24) 0.0087

rs74491133 C/T *CC* *CT* *TT*  *CC* *CT* *TT*

4630 (97.64) 112 (2.36) 0 (0) 1.0000 8432 (97.25) 237 (2.73) 2 (0.02) 0.6839

____________________________________________________________________________________________________________

Data are numbers of subjects (percentages). H-W *P*, *P* value for Hardy-Weinberg equilibrium.
