# Supplementary material for: Identification of eight genetic variants as novel determinants of dyslipidemia in Japanese by exome-wide association studies
Source: Oncotarget. 2017 Apr 17;8(24):38950–61. doi: 10.18632/oncotarget.17159 (PMC5503585; doi:10.18632/oncotarget.17159)
Supplement: Supplementary file 7 [file oncotarget-08-38950-s007.docx]

**Supplementary Table 6.** Genotype distributions for SNPs associated (*P* < 1.21 × 10^–6^) with hypo-HDL-cholesterolemia in the EWAS.

____________________________________________________________________________________________________________

SNP Hypo–HDL-cholesterolemia H-W *P* Controls H-W *P*

____________________________________________________________________________________________________________

rs2561111 G/A (R111H) *GG* *GA* *AA* *GG* *GA* *AA*

1946 (73.55) 646 (24.41) 54 (2.04) 0.9367 8402 (73.24) 2818 (24.56) 252 (2.20) 0.3859

rs3745486 T/C (L99P) *TT* *TC* *CC*  *TT* *TC* *CC*

930 (35.16) 1280 (48.39) 435 (16.45) 0.9038 4081 (35.58) 5526 (48.17) 1864 (16.25) 0.9382

rs77885682 C/T (R503K) *CC* *CT* *TT*  *CC* *CT* *TT*

2407 (90.97) 231 (8.73) 8 (0.30) 0.2786 10378 (90.46) 1063 (9.26) 32 (0.28) 0.3682

rs61748749 T/G (S1353R) *TT* *TG* *GG*  *TT* *TG* *GG*

2524 (95.39) 122 (4.61) 0 (0) 0.4017 10954 (95.49) 506 (4.41) 12 (0.10) 0.0219

rs143822500 T/C (T867A) *TT* *TC* *CC*  *TT* *TC* *CC*

2620 (99.02) 26 (0.98) 0 (0) 1.0000 11313 (98.60) 159 (1.39) 1 (0.01) 0.4318

rs857591 G/T *GG* *GT* *TT*  *GG* *GT* *TT*

933 (35.26) 1306 (49.36) 407 (15.38) 0.1568 4211 (36.71) 5431 (47.34) 1830 (15.95) 0.2578

rs499974 G/T *GG* *GT* *TT*  *GG* *GT* *TT*

1232 (46.56) 1115 (42.14) 299 (11.30) 0.0562 5396 (47.04) 4918 (42.87) 1158 (10.09) 0.4492

rs184499441 C/T (G1288R) *CC* *CT* *TT*  *CC* *CT* *TT*

2563 (96.86) 82 (3.10) 1 (0.04) 0.4879 11114 (96.87) 356 (3.10) 3 (0.03) 0.7632

rs1053966 C/G (H1751D) *CC* *CG* *GG*  *CC* *CG* *GG*

1773 (67.03) 789 (29.83) 83 (3.14) 0.7424 7758 (67.64) 3321 (28.95) 391 (3.41) 0.1270

rs12632942 A/G (L1092P) *AA* *AG* *GG*  *AA* *AG* *GG*

714 (26.99) 1326 (50.11) 606 (22.90) 0.8762 3313 (28.88) 5656 (49.31) 2501 (21.81) 0.3480

rs150294461 G/A (G654E) *GG* *GA* *AA* *GG* *GA* *AA*

2579 (97.47) 67 (2.53) 0 (0) 1.0000 11167 (97.33) 304 (2.65) 2 (0.02) 1.0000

rs3750208 G/A (R168W) *GG* *GA* *AA* *GG* *GA* *AA*

2068 (78.22) 540 (20.42) 36 (1.36) 0.9242 9030 (78.75) 2277 (19.86) 159 (1.39) 0.2639

rs2255317 C/T (T855M) *CC* *CT* *TT*  *CC* *CT* *TT*

1900 (71.81) 683 (25.81) 63 (2.38) 0.8806 8314 (72.47) 2868 (25.00) 290 (2.53) 0.0257

rs145716748 A/G (S729P) *AA* *AG* *GG*  *AA* *AG* *GG*

2544 (96.15) 102 (3.85) 0 (0) 0.6249 11058 (96.38) 410 (3.58) 5 (0.04) 0.4385

rs10191097 T/G *TT* *TG* *GG*  *TT* *TG* *GG*

1316 (49.74) 1096 (41.42) 234 (8.84) 0.7795 5769 (50.29) 4730 (41.23) 973 (8.48) 0.9459

rs148320716 C/T (R96C) *CC* *CT* *TT*  *CC* *CT* *TT*

2496 (94.33) 148 (5.59) 2 (0.08) 1.0000 10890 (94.93) 572 (4.99) 9 (0.08) 0.5729

rs2153157 T/C *TT* *TC* *CC*  *TT* *TC* *CC*

1309 (49.47) 1107 (41.84) 230 (8.69) 0.8887 5526 (48.17) 4881 (42.55) 1064 (9.28) 0.7917

rs7299095 G/A *GG* *GA* *AA* *GG* *GA* *AA*

982 (37.14) 1243 (47.01) 419 (15.85) 0.4390 4073 (35.51) 5462 (47.61) 1936 (16.88) 0.1523

rs17852959 C/T (V161M) *CC* *CT* *TT*  *CC* *CT* *TT*

1836 (69.39) 731 (27.63) 79 (2.98) 0.5318 7986 (69.61) 3186 (27.77) 301 (2.62) 0.4562

rs11629205 G/A *GG* *GA* *AA* *GG* *GA* *AA*

1185 (44.80) 1149 (43.44) 311 (11.76) 0.2056 5093 (44.40) 5122 (44.65) 1256 (10.95) 0.5702

rs3729740 C/T (D578N) *CC* *CT* *TT*  *CC* *CT* *TT*

1501 (56.77) 976 (36.91) 167 (6.32) 0.6386 6633 (57.82) 4191 (36.54) 647 (5.64) 0.6814

rs150534954 C/G (C155W) *CC* *CG* *GG*  *CC* *CG* *GG*

1940 (97.34) 51 (2.56) 2 (0.10) 0.0521 11163 (97.30) 306 (2.67) 4 (0.03) 0.1670

rs11180311 A/G *AA* *AG* *GG*  *AA* *AG* *GG*

1046 (39.53) 1207 (45.62) 393 (14.85) 0.1473 4391 (38.27) 5434 (47.36) 1648 (14.37) 0.6206

rs11877062 T/C (W4R) *TT* *TC* *CC*  *TT* *TC* *CC*

766 (28.98) 1312 (49.64) 565 (21.38) 0.9377 3293 (28.71) 5706 (49.74) 2472 (21.55) 1.0000

rs3747203 T/C (R199G) *TT* *TC* *CC*  *TT* *TC* *CC*

1721 (65.07) 809 (30.58) 115 (4.35) 0.1095 7332 (63.91) 3692 (32.18) 449 (3.91) 0.5794

rs78002652 G/A (G139R) *GG* *GA* *AA* *GG* *GA* *AA*

2631 (99.43) 15 (0.57) 0 (0) 1.0000 11404 (99.40) 68 (0.59) 1 (0.01) 0.1002

rs151000241 T/C (N190S) *TT* *TC* *CC*  *TT* *TC* *CC*

2638 (99.70) 8 (0.30) 0 (0) 1.0000 11432 (99.64) 41 (0.36) 0 (0) 1.0000

rs6847454 A/T (Q453L) *AA* *AT* *TT*  *AA* *AT* *TT*

1490 (56.35) 980 (37.07) 174 (6.58) 0.4691 6321 (55.16) 4387 (38.28) 752 (6.56) 0.8258

rs143833298 G/A (R830Q) *GG* *GA* *AA* *GG* *GA* *AA*

2597 (98.15) 49 (1.85) 0 (0) 1.0000 11292 (98.42) 180 (1.57) 1 (0.01) 0.5150

rs200295807 G/A (R26C) *GG* *GA* *AA* *GG* *GA* *AA*

2630 (99.40) 16 (0.60) 0 (0) 1.0000 11393 (99.30) 80 (0.70) 0 (0) 1.0000

rs199606102 C/T (V695I) *CC* *CT* *TT*  *CC* *CT* *TT*

2609 (98.60) 37 (1.40) 0 (0) 1.0000 11307 (98.55) 162 (1.41) 4 (0.04) 0.0035

rs10100935 C/T (A118T) *CC* *CT* *TT*  *CC* *CT* *TT*

1108 (41.87) 1220 (46.11) 318 (12.02) 0.5501 4768 (41.61) 5235 (45.68) 1457 (12.71) 0.7290

rs586088 A/T (T190S) *AA* *AT* *TT*  *AA* *AT* *TT*

1203 (45.47) 1158 (43.76) 285 (10.77) 0.7910 5437 (47.40) 4877 (42.52) 1156 (10.08) 0.2005

rs146515657 T/C (N650S) *TT* *TC* *CC*  *TT* *TC* *CC*

1878 (97.97) 39 (2.03) 0 (0) 1.0000 9599(99.94) 6 (0.06) 0 (0) 1.0000

rs4807160 C/T (E144K) *CC* *CT* *TT*  *CC* *CT* *TT*

1223 (46.34) 1144 (43.35) 272 (10.31) 0.8580 5209(45.61) 4994(43.73) 1218(10.66) 0.6855

rs4838865 G/A (S567L) *GG* *GA* *AA* *GG* *GA* *AA*

1803 (68.14) 759 (28.69) 84 (3.17) 0.6868 7940 (69.23) 3182 (27.74) 347 (3.03) 0.1947

rs17053501 C/T *CC* *CT* *TT*  *CC* *CT* *TT*

2624 (99.17) 22 (0.83) 0 (0) 1.0000 11386 (99.24) 86 (0.75) 1 (0.01) 0.1542

rs146600946 G/A (R286H) *GG* *GA* *AA* *GG* *GA* *AA*

2554 (96.52) 91 (3.44) 1 (0.04) 0.5608 11025 (96.10) 444 (3.87) 4 (0.03) 1.0000

rs143827332 G/A (R1035W) *GG* *GA* *AA* *GG* *GA* *AA*

2615 (98.83) 30 (1.13) 1 (0.04) 0.0900 11348 (98.91) 124 (1.08) 1 (0.01) 0.2918

rs1823068 A/G *AA* *AG* *GG*  *AA* *AG* *GG*

1621 (61.31) 900 (34.04) 123 (4.65) 0.9543 7014 (61.17) 3884 (33.87) 569 (4.96) 0.2995

rs7997737 G/A *GG* *GA* *AA* *GG* *GA* *AA*

820 (31.00) 1296 (49.00) 529 (20.00) 0.6938 3507 (30.58) 5669 (49.43) 2293 (19.99) 0.9849

rs9901755 A/G *AA* *AG* *GG*  *AA* *AG* *GG*

809 (30.59) 1284 (48.54) 552 (20.87) 0.3075 3399 (29.63) 5599 (48.80) 2475 (21.57) 0.0602

rs150412190 G/A (S116L) *GG* *GA* *AA* *GG* *GA* *AA*

2628 (99.32) 18 (0.68) 0 (0) 1.0000 11404 (99.40) 69 (0.60) 0 (0) 1.0000

rs7004867 C/T (R31Q) *CC* *CT* *TT*  *CC* *CT* *TT*

2627 (99.28) 19 (0.72) 0 (0) 1.0000 11411 (99.46) 62 (0.54) 0 (0) 1.0000

rs2271251 C/G (A82G) *CC* *CG* *GG*  *CC* *CG* *GG*

2606 (98.49) 40 (1.51) 0 (0) 1.0000 11306 (98.55) 164 (1.43) 2 (0.02) 0.1247

rs62623665 G/A (R352H) *GG* *GA* *AA* *GG* *GA* *AA*

2634 (99.55) 12 (0.45) 0 (0) 1.0000 11434 (99.67) 38 (0.33) 0 (0) 1.0000

rs57075420 C/T (R297C) *CC* *CT* *TT*  *CC* *CT* *TT*

2548 (96.30) 96 (3.63) 2 (0.07) 0.2400 11073 (96.51) 396 (3.45) 4 (0.04) 0.7828

rs7442317 G/A *GG* *GA* *AA* *GG* *GA* *AA*

972 (36.75) 1258 (47.56) 415 (15.69) 0.8072 4433 (38.65) 5342 (46.57) 1696 (14.78) 0.1847

rs3820678 G/A (A191T) *GG* *GA* *AA* *GG* *GA* *AA*

1670 (63.11) 854 (32.28) 122 (4.61) 0.3442 7157 (62.39) 3802 (33.15) 512 (4.46) 0.8003

rs10794531 C/T (R53H) *CC* *CT* *TT*  *CC* *CT* *TT*

1033 (39.04) 1227 (46.37) 386 (14.59) 0.4823 4517 (39.37) 5315 (46.33) 1641 (14.30) 0.2243

rs78943519 G/A (G15D) *GG* *GA* *AA* *GG* *GA* *AA*

1437 (54.97) 995 (38.07) 182 (6.96) 0.5763 6211 (54.58) 4379 (38.48) 790 (6.94) 0.6277

rs149721746 A/G (Y60C) *AA* *AG* *GG*  *AA* *AG* *GG*

2595 (98.45) 41 (1.55) 0 (0) 1.0000 11181 (97.82) 248 (2.17) 1 (0.01) 1.0000

rs7619670 G/A *GG* *GA* *AA* *GG* *GA* *AA*

1752 (66.21) 782 (29.56) 112 (4.23) 0.0431 7499 (65.36) 3523 (30.71) 451 (3.93) 0.1499

rs199763816 G/A (P1116S) *GG* *GA* *AA* *GG* *GA* *AA*

2642 (99.85) 4 (0.15) 0 (0) 1.0000 11443 (99.74) 30 (0.26) 0 (0) 1.0000

rs1555494 A/G *AA* *AG* *GG*  *AA* *AG* *GG*

2642 (99.85) 4 (0.15) 0 (0) 1.0000 11448 (99.78) 25 (0.22) 0 (0) 1.0000

rs2049805 C/T *CC* *CT* *TT*  *CC* *CT* *TT*

1797 (67.91) 754 (28.50) 95 (3.59) 0.1633 7796 (67.95) 3306 (28.82) 371 (3.23) 0.3684

rs11247229 C/T *CC* *CT* *TT*  *CC* *CT* *TT*

1172 (44.29) 1196 (45.20) 278 (10.51) 0.3127 5008 (43.65) 5120 (44.63) 1345 (11.72) 0.5059

rs6598858 C/T *CC* *CT* *TT*  *CC* *CT* *TT*

1676 (63.34) 840 (31.75) 130 (4.91) 0.0671 7464 (65.07) 3552 (30.96) 455 (3.97) 0.2107

rs147317864 C/T (A262T) *CC* *CT* *TT*  *CC* *CT* *TT*

1904 (99.17) 16 (0.83) 0 (0) 1.0000 9873 (100.00) 0 (0) 0 (0) ND

rs118174683 G/A (T482M) *GG* *GA* *AA* *GG* *GA* *AA*

2640 (99.77) 6 (0.23) 0 (0) 1.0000 11448 (99.78) 25 (0.22) 0 (0) 1.0000

rs1536690 C/T (P72L) *CC* *CT* *TT*  *CC* *CT* *TT*

2219 (83.86) 406 (15.35) 21 (0.79) 0.6149 9605 (83.72) 1790 (15.60) 78 (0.68) 0.6305

rs17316633 G/A *GG* *GA* *AA* *GG* *GA* *AA*

2163 (81.78) 468 (17.69) 14 (0.53) 0.0384 9340 (81.42) 2022 (17.62) 110 (0.96) 0.9578

rs139476663 T/C (V87A) *TT* *TC* *CC*  *TT* *TC* *CC*

2623 (99.13) 22 (0.83) 1 (0.04) 0.0510 11373 (99.13) 100 (0.87) 0 (0) 1.0000

rs8059612 G/A *GG* *GA* *AA* *GG* *GA* *AA*

936 (35.37) 1275 (48.19) 435 (16.44) 1.0000 4174 (36.39) 5445 (47.46) 1853 (16.15) 0.2756

rs2032794 A/G *AA* *AG* *GG*  *AA* *AG* *GG*

1582 (59.81) 928 (35.09) 135 (5.10) 1.0000 6807 (59.33) 4047 (35.27) 619 (5.40) 0.5803

rs992822 G/A (S910N) *GG* *GA* *AA* *GG* *GA* *AA*

717 (27.23) 1277 (48.50) 639 (24.27) 0.1382 3204 (27.99) 5694 (49.74) 2550 (22.27) 0.8366

rs17028450 C/T (R690C) *CC* *CT* *TT*  *CC* *CT* *TT*

2585 (97.70) 61 (2.30) 0 (0) 1.0000 11222 (97.81) 250 (2.18) 1 (0.01) 1.0000

rs1980889 A/G *AA* *AG* *GG*  *AA* *AG* *GG*

1053 (39.80) 1207 (45.61) 386 (14.59) 0.1842 4487 (39.17) 5328 (46.52) 1639 (14.31) 0.3701

rs1464890 C/T (A271T) *CC* *CT* *TT*  *CC* *CT* *TT*

1409 (53.25) 1037 (39.19) 200 (7.56) 0.6234 6015 (52.43) 4580 (39.92) 878 (7.65) 0.8886

rs2324027 C/T *CC* *CT* *TT*  *CC* *CT* *TT*

711 (26.87) 1305 (49.32) 630 (23.81) 0.5085 3096 (26.99) 5675 (49.47) 2701 (23.54) 0.3127

rs7667636 G/A *GG* *GA* *AA* *GG* *GA* *AA*

691 (26.12) 1314 (49.68) 640 (24.20) 0.7557 2927 (25.51) 5671 (49.43) 2875 (25.06) 0.2249

rs10943613 T/C *TT* *TC* *CC*  *TT* *TC* *CC*

1111 (41.99) 1203 (45.46) 332 (12.55) 0.8314 4874 (42.48) 5201 (45.34) 1397 (12.18) 0.8694

rs948962 C/A (L1954I) *CC* *CA* *AA*  *CC* *CA* *AA*

1285 (48.58) 1100 (41.59) 260 (9.83) 0.2723 5708 (49.76) 4730 (41.23) 1033 (9.01) 0.2354

rs76022391 G/A (G668S) *GG* *GA* *AA* *GG* *GA* *AA*

2515 (95.05) 130 (4.91) 1 (0.04) 1.0000 10812 (94.24) 649 (5.66) 12 (0.10) 0.5085

rs7913069 C/T *CC* *CT* *TT*  *CC* *CT* *TT*

2244 (84.81) 376 (14.21) 26 (0.98) 0.0349 9829 (85.70) 1565 (13.65) 75 (0.65) 0.1378

rs200330080 C/T (R654Q) *CC* *CT* *TT*  *CC* *CT* *TT*

2569 (97.09) 76 (2.87) 1 (0.04) 0.4378 11081 (96.59) 387 (3.37) 4 (0.04) 0.5844

rs111765932 T/C (I926T) *TT* *TC* *CC*  *TT* *TC* *CC*

2639 (99.74) 7 (0.26) 0 (0) 1.0000 11433 (99.66) 39 (0.34) 0 (0) 1.0000

rs141510612 G/A (R690Q) *GG* *GA* *AA* *GG* *GA* *AA*

2639 (99.74) 7 (0.26) 0 (0) 1.0000 11446 (99.77) 27 (0.23) 0 (0) 1.0000

rs74844425 T/C (I15V) *TT* *TC* *CC*  *TT* *TC* *CC*

2615 (98.83) 31 (1.17) 0 (0) 1.0000 11322 (98.68) 149 (1.30) 2 (0.02) 0.0913

rs12229654 T/G *TT* *TG* *GG*  *TT* *TG* *GG*

1451 (54.84) 1030 (38.93) 165 (6.23) 0.3338 6973 (60.80) 3908 (34.08) 587 (5.12) 0.1939

rs145141779 G/A (P337L) *GG* *GA* *AA* *GG* *GA* *AA*

2485 (93.91) 159 (6.01) 2 (0.08) 1.0000 10720 (93.45) 739 (6.44) 12 (0.11) 1.0000

rs13188074 A/G *AA* *AG* *GG*  *AA* *AG* *GG*

1238 (46.81) 1130 (42.72) 277 (10.47) 0.4202 5501 (47.95) 4853 (42.30) 1118 (9.75) 0.3146

rs56369596 G/C (V699L) *GG* *GC* *CC*  *GG* *GC* *CC*

2516 (95.09) 130 (4.91) 0 (0) 0.4070 10872 (94.76) 588 (5.13) 13 (0.11) 0.1021

rs874478 G/A *GG* *GA* *AA* *GG* *GA* *AA*

1466 (55.40) 1001 (37.83) 179 (6.77) 0.6468 6547 (57.07) 4154 (36.21) 771 (6.72) 0.0015

rs202021460 C/T (A252T) *CC* *CT* *TT*  *CC* *CT* *TT*

2637 (99.66) 9 (0.34) 0 (0) 1.0000 11447 (99.77) 26 (0.23) 0 (0) 1.0000

rs179075 T/C *TT* *TC* *CC*  *TT* *TC* *CC*

1164 (44.01) 1170 (44.23) 311 (11.76) 0.5152 5017 (43.74) 5150 (44.91) 1302 (11.35) 0.7385

rs6699355 C/T *CC* *CT* *TT*  *CC* *CT* *TT*

1174 (44.47) 1197 (45.34) 269 (10.19) 0.1714 5196 (45.32) 4998 (43.59) 1271 (11.09) 0.1896

rs874889 A/C (I267M) *AA* *AC* *CC*  *AA* *AC* *CC*

717 (27.10) 1346 (50.87) 583 (22.03) 0.3110 3288 (28.66) 5653 (49.28) 2531 (22.06) 0.2767

____________________________________________________________________________________________________________

Data are numbers of subjects (percentages). H-W *P*, *P* value for Hardy-Weinberg equilibrium; ND, not determined.
