# Supplementary material for: Identification of eight genetic variants as novel determinants of dyslipidemia in Japanese by exome-wide association studies
Source: Oncotarget. 2017 Apr 17;8(24):38950–61. doi: 10.18632/oncotarget.17159 (PMC5503585; doi:10.18632/oncotarget.17159)
Supplement: Supplementary file 8 [file oncotarget-08-38950-s008.docx]

**Supplementary Table 7.** Genotype distributions for SNPs associated (*P* < 1.21 × 10^–6^) with hyper–LDL-cholesterolemia in the EWAS.

____________________________________________________________________________________________________________

SNP Hyper–LDL-cholesterolemia H-W *P* Controls H-W *P*

____________________________________________________________________________________________________________

rs2125904 C/A *CC* *CA* *AA* *CC* *CA* *AA*

2406 (53.60) 1730 (38.54) 353 (7.86) 0.0895 4857 (53.45) 3599 (39.60) 632 (6.95) 0.3353

rs2959953 C/G (P213R) *CC* *CG* *GG*  *CC* *CG* *GG*

1129 (25.29) 2249 (50.37) 1087 (24.34) 0.6320 2476 (27.44) 4412 (48.89) 2136 (23.67) 0.0499

rs12144325 A/G (L366P) *AA* *AG* *GG*  *AA* *AG* *GG*

4251 (94.82) 228 (5.09) 4 (0.09) 0.5520 8591 (94.80) 464 (5.12) 7 (0.08) 0.6821

rs2496425 T/C (F1070S) *TT* *TC* *CC*  *TT* *TC* *CC*

1659 (36.96) 2179 (48.54) 651 (14.50) 0.1396 3473 (38.22) 4315 (47.48) 1300 (14.30) 0.5044

rs10097386 T/C *TT* *TC* *CC*  *TT* *TC* *CC*

1385 (30.86) 2168 (48.31) 935 (20.83) 0.1100 2781 (30.60) 4541 (49.97) 1766 (19.43) 0.2602

rs7120775 C/G (Y27*) *CC* *CG* *GG*  *CC* *CG* *GG*

3115 (69.41) 1241 (27.65) 132 (2.94) 0.5213 6300 (69.32) 2513 (27.65) 275 (3.03) 0.2032

rs138559558 G/A (R289C) *GG* *GA* *AA*  *GG* *GA* *AA*

4385 (97.68) 104 (2.32) 0 (0) 1.0000 8862 (97.51) 224 (2.47) 2 (0.02) 0.6547

rs7532317 C/A *CC* *CA* *AA* *CC* *CA* *AA*

1727 (38.49) 2111 (47.05) 649 (14.46) 0.9243 3595 (39.56) 4139 (45.55) 1353 (14.89) 0.0042

rs4132509 C/A *CC* *CA* *AA* *CC* *CA* *AA*

2349 (52.34) 1806 (40.24) 333 (7.42) 0.6006 4754 (52.32) 3617 (39.80) 716 (7.88) 0.4484

rs5716 G/C (K367N) *GG* *GC* *CC*  *GG* *GC* *CC*

3053 (68.01) 1288 (28.69) 148 (3.30) 0.4111 6199 (68.22) 2631 (28.95) 257 (2.83) 0.2877

rs7771335 A/G *AA* *AG* *GG*  *AA* *AG* *GG*

2520 (56.15) 1642 (36.59) 326 (7.26) 0.0107 5661 (62.30) 2961 (32.59) 464 (5.11) 0.0030

rs79980197 C/G (P706R) *CC* *CG* *GG*  *CC* *CG* *GG*

4218 (93.96) 265 (5.90) 6 (0.14) 0.3208 8612 (94.76) 468 (5.15) 8 (0.09) 0.5388

rs201078160 C/T (R4162Q) *CC* *CT* *TT*  *CC* *CT* *TT*

4474 (99.67) 15 (0.33) 0 (0) 1.0000 9070 (99.81) 17 (0.19) 0 (0) 1.0000

rs10770 T/C (I180T) *TT* *TC* *CC*  *TT* *TC* *CC*

3690 (82.20) 765 (17.04) 34 (0.76) 0.4777 7463 (82.12) 1518 (16.70) 107 (1.18) 0.0041

rs76974938 C/T (D67N) *CC* *CT* *TT*  *CC* *CT* *TT*

3984 (97.12) 118 (2.88) 0 (0) 1.0000 7605 (94.23) 466 (5.77) 0 (0) 0.0021

rs76828246 G/A (S277N) *GG* *GA* *AA*  *GG* *GA* *AA*

4477 (99.73) 12 (0.27) 0 (0) 1.0000 9054 (99.63) 34 (0.37) 0 (0) 1.0000

rs2071653 C/T *CC* *CT* *TT*  *CC* *CT* *TT*

2271 (50.59) 1775 (39.54) 443 (9.87) 0.0005 5076 (55.85) 3363 (37.01) 649 (7.14) 0.0050

rs10778257 T/G (E11D) *TT* *TG* *GG*  *TT* *TG* *GG*

1130 (25.22) 2203 (49.16) 1148 (25.62) 0.2689 2323 (25.71) 4433 (49.07) 2278 (25.22) 0.0772

rs6901 C/T (R1038Q) *CC* *CT* *TT*  *CC* *CT* *TT*

2570 (57.25) 1656 (36.89) 263 (5.86) 0.9031 5330 (58.67) 3247 (35.74) 508 (5.59) 0.6403

rs200400344 G/A (R366Q) *GG* *GA* *AA*  *GG* *GA* *AA*

4442 (98.95) 47 (1.05) 0 (0) 1.0000 9012 (99.16) 75 (0.83) 1 (0.01) 0.1493

rs7793970 G/A *GG* *GA* *AA*  *GG* *GA* *AA*

1187 (26.44) 2218 (49.41) 1084 (24.15) 0.4553 2339 (25.74) 4508 (49.62) 2239 (24.64) 0.4756

rs56133554 T/G (T1077P) *TT* *TG* *GG*  *TT* *TG* *GG*

4157 (92.71) 321 (7.16) 6 (0.13) 1.0000 8434 (93.00) 623 (6.87) 12 (0.13) 0.8780

rs150412190 G/A (S116L) *GG* *GA* *AA*  *GG* *GA* *AA*

4461 (99.38) 28 (0.62) 0 (0) 1.0000 9035 (99.42) 53 (0.58) 0 (0) 1.0000

rs3797036 A/C (N456K) *AA* *AC* *CC*  *AA* *AC* *CC*

4317 (96.19) 168 (3.74) 3 (0.07) 0.2350 8694 (95.69) 384 (4.22) 8 (0.09) 0.0847

rs60312980 G/T *GG* *GT* *TT*  *GG* *GT* *TT*

4145 (92.34) 339 (7.55) 5 (0.11) 0.6870 8477 (93.28) 598 (6.58) 13 (0.14) 0.4282

rs146243553 A/G (S67P) *AA* *AG* *GG*  *AA* *AG* *GG*

4443 (98.98) 46 (1.02) 0 (0) 1.0000 8999 (99.02) 88 (0.97) 1 (0.01) 0.1986

rs1757106 T/G *TT* *TG* *GG*  *TT* *TG* *GG*

1463 (32.59) 2192 (48.83) 834 (18.58) 0.8076 3158 (34.75) 4379 (48.18) 1551 (17.07) 0.6185

rs117135042 C/T (S634L) *CC* *CT* *TT*  *CC* *CT* *TT*

4212 (93.83) 270 (6.01) 7 (0.16) 0.2164 8540 (93.97) 541 (5.95) 7 (0.08) 0.7245

rs1292053 G/A (T76M) *GG* *GA* *AA*  *GG* *GA* *AA*

1611 (35.90) 2123 (47.32) 753 (16.78) 0.2389 3178 (34.97) 4385 (48.26) 1524 (16.77) 0.8622

rs151252589 T/G (F327L) *TT* *TG* *GG*  *TT* *TG* *GG*

4369 (97.33) 120 (2.67) 0 (0) 1.0000 8854 (97.43) 231 (2.54) 3 (0.03) 0.1994

rs11101224 G/A (T428M) *GG* *GA* *AA*  *GG* *GA* *AA*

3819 (85.07) 628 (13.99) 42 (0.94) 0.0075 7717 (84.91) 1323 (14.56) 48 (0.53) 0.3077

rs2853969 C/T *CC* *CT* *TT*  *CC* *CT* *TT*

3495 (77.98) 916 (20.44) 71 (1.58) 0.2223 7485 (82.55) 1483 (16.36) 99 (1.09) 0.0103

rs2835655 G/A *GG* *GA* *AA*  *GG* *GA* *AA*

1220 (27.18) 2237 (49.85) 1031 (22.97) 0.9285 2445 (26.90) 4482 (49.32) 2161 (23.78) 0.2313

rs202169174 T/C (D1528G) *TT* *TC* *CC*  *TT* *TC* *CC*

4473 (99.71) 13 (0.29) 0 (0) 1.0000 9060 (99.69) 28 (0.31) 0 (0) 1.0000

rs151324745 A/C (Y1136D) *AA* *AC* *CC*  *AA* *AC* *CC*

4390 (97.80) 99 (2.20) 0 (0) 1.0000 8886 (97.78) 201 (2.21) 1 (0.01) 1.0000

rs2395402 T/C *TT* *TC* *CC*  *TT* *TC* *CC*

2935 (65.38) 1379 (30.72) 175 (3.40) 0.4146 6181 (68.01) 2610 (28.72) 297 (3.27) 0.2948

rs783540 G/A *GG* *GA* *AA*  *GG* *GA* *AA*

1125 (25.06) 2208 (49.19) 1156 (25.75) 0.2825 2329 (25.64) 4467 (49.17) 2289 (25.19) 0.1155

rs147989324 G/A (V304I) *GG* *GA* *AA*  *GG* *GA* *AA*

4358 (99.66) 15 (0.34) 0 (0) 1.0000 9052 (99.66) 31 (0.34) 0 (0) 1.0000

rs11543598 G/A (P213L) *GG* *GA* *AA*  *GG* *GA* *AA*

4333 (96.53) 155 (3.45) 1 (0.02) 1.0000 8795 (96.80) 289 (3.18) 2 (0.02) 1.0000

rs7124275 T/C *TT* *TC* *CC*  *TT* *TC* *CC*

1328 (29.60) 2247 (50.08) 912 (20.32) 0.5076 2758 (30.35) 4443 (48.89) 1886 (20.76) 0.2194

rs6801425 A/G *AA* *AG* *GG*  *AA* *AG* *GG*

1615 (35.98) 2141 (47.71) 732 (16.31) 0.6193 3379 (37.18) 4279 (47.08) 1430 (15.74) 0.2182

rs200636353 G/A (G34S) *GG* *GA* *AA*  *GG* *GA* *AA*

4429 (99.24) 33 (0.74) 1 (0.02) 0.0647 9025 (99.42) 53 (0.58) 0 (0) 1.0000

rs3746875 A/C (M30L) *AA* *AC* *CC*  *AA* *AC* *CC*

4412 (98.29) 75 (1.67) 2 (0.04) 0.0456 8930 (98.26) 157 (1.73) 1 (0.01) 0.5020

rs1007160 G/T (L223M) *GG* *GT* *TT*  *GG* *GT* *TT*

2162 (48.16) 1871 (41.68) 456 (10.16) 0.0873 4373 (48.12) 3837 (42.22) 878 (9.66) 0.3889

rs7305779 A/C (E103A) *AA* *AC* *CC*  *AA* *AC* *CC*

3639 (81.06) 799 (17.80) 51 (1.14) 0.3219 7392 (81.34) 1603 (17.64) 93 (1.02) 0.5544

rs17232910 G/C (A643P) *GG* *GC* *CC*  *GG* *GC* *CC*

3601 (80.22) 831 (18.51) 57 (1.27) 0.2666 7372 (81.14) 1619 (17.82) 95 (1.04) 0.5583

rs1152522 C/T *CC* *CT* *TT*  *CC* *CT* *TT*

4129 (91.98) 348 (7.75) 12 (0.27) 0.1275 8289 (91.21) 774 (8.52) 25 (0.27) 0.1436

rs61308377 A/G (Y209H) *AA* *AG* *GG*  *AA* *AG* *GG*

2776 (61.87) 1506 (33.56) 205 (4.57) 0.9646 5529 (60.92) 3133 (34.52) 414 (4.56) 0.2815

rs144335584 A/G (I747V) *AA* *AG* *GG*  *AA* *AG* *GG*

4415 (98.37) 72 (1.61) 1 (0.02) 0.2617 8906 (98.00) 180 (1.98) 2 (0.02) 0.2369

rs3775948 G/C *GG* *GC* *CC*  *GG* *GC* *CC*

1530 (34.08) 2171 (48.36) 788 (17.56) 0.7127 3008 (33.10) 4398 (48.40) 1681 (18.50) 0.3035

rs12669721 G/T (P119T) *GG* *GT* *TT*  *GG* *GT* *TT*

2758 (61.44) 1511 (33.66) 220 (4.90) 0.4827 5561 (61.20) 3093 (34.04) 433 (4.76) 0.9020

rs3944066 C/T (P494L) *CC* *CT* *TT*  *CC* *CT* *TT*

4398 (97.97) 89 (1.98) 2 (0.05) 0.0815 8907 (98.01) 179 (1.97) 2 (0.02) 0.2331

rs144134358 C/T (S569L) *CC* *CT* *TT*  *CC* *CT* *TT*

4481 (99.82) 8 (0.18) 0 (0) 1.0000 9057 (99.66) 31 (0.34) 0 (0) 1.0000

rs199905767 C/A (P620T) *CC* *CA* *AA* *CC* *CA* *AA*

4480 (99.80) 9 (0.20) 0 (0) 1.0000 9060 (99.70) 27 (0.30) 0 (0) 1.0000

rs41284134 G/C (G245R) *GG* *GC* *CC*  *GG* *GC* *CC*

4381 (97.60) 107 (2.38) 1 (0.02) 0.4850 8894 (97.87) 192 (2.11) 2 (0.02) 0.2833

rs201453898 C/T (R547Q) *CC* *CT* *TT*  *CC* *CT* *TT*

4471 (99.60) 18 (0.40) 0 (0) 1.0000 9043 (99.51) 45 (0.49) 0 (0) 1.0000

rs2269704 C/T *CC* *CT* *TT*  *CC* *CT* *TT*

3232 (72.05) 1153 (25.70) 101 (2.25) 0.9074 6970 (76.72) 1944 (21.40) 171 (1.88) 0.0114

rs115387731 C/T (V8I) *CC* *CT* *TT*  *CC* *CT* *TT*

4473 (99.64) 16 (0.36) 0 (0) 1.0000 9044 (99.52) 44 (0.48) 0 (0) 1.0000

rs2269703 G/A *GG* *GA* *AA*  *GG* *GA* *AA*

3232 (72.10) 1150 (25.65) 101 (2.25) 0.9535 6969 (76.75) 1939 (21.36) 172 (1.89) 0.0075

rs1264318 G/C *GG* *GC* *CC*  *GG* *GC* *CC*

3992 (88.93) 479 (10.67) 18 (0.40) 0.3355 8032 (88.38) 1030 (11.33) 26 (0.29) 0.3016

rs10917536 G/T (Q72K) *GG* *GT* *TT*  *GG* *GT* *TT*

1732 (38.61) 2124 (47.35) 630 (14.04) 0.6113 3586 (39.46) 4198 (46.20) 1303 (14.34) 0.1940

rs148768286 C/T (V266I) *CC* *CT* *TT*  *CC* *CT* *TT*

4440 (98.91) 49 (1.09) 0 (0) 1.0000 8976 (98.77) 112 (1.23) 0 (0) 1.0000

rs7442317 G/A *GG* *GA* *AA*  *GG* *GA* *AA*

1684 (37.52) 2113 (47.08) 691 (15.40) 0.5098 3517 (38.71) 4226 (46.51) 1343 (14.78) 0.2046

rs12069239 G/C (A471P) *GG* *GC* *CC*  *GG* *GC* *CC*

3160 (70.41) 1221 (27.21) 107 (2.38) 0.4364 6493 (71.44) 2359 (25.96) 236 (2.60) 0.2163

rs9469042 T/C *TT* *TC* *CC*  *TT* *TC* *CC*

3938 (87.73) 523 (11.65) 28 (0.62) 0.0255 7894 (86.76) 1159 (12.76) 44 (0.48) 0.8056

rs618662 C/A *CC* *CA* *AA* *CC* *CA* *AA*

2173 (48.41) 1917 (42.70) 399 (8.89) 0.4364 4494 (49.45) 3784 (41.64) 810 (8.91) 0.7441

rs8133766 T/C *TT* *TC* *CC*  *TT* *TC* *CC*

1563 (34.86) 2188 (48.79) 733 (16.35) 0.4963 3271 (36.00) 4333 (47.69) 1482 (16.31) 0.4581

rs117778870 G/A (R40H) *GG* *GA* *AA*  *GG* *GA* *AA*

4345 (96.79) 143 (3.19) 1 (0.02) 1.0000 8778 (96.59) 307 (3.38) 3 (0.03) 0.7518

rs140750531 G/A (R551H) *GG* *GA* *AA*  *GG* *GA* *AA*

4341 (96.70) 146 (3.25) 2 (0.05) 0.3549 8736 (96.13) 346 (3.81) 6 (0.06) 0.1682

rs4953863 C/T *CC* *CT* *TT*  *CC* *CT* *TT*

1182 (26.33) 2198 (48.96) 1109 (24.71) 0.1697 2372 (26.10) 4568 (50.26) 2148 (23.64) 0.5852

rs74546291 G/A (W240*) *GG* *GA* *AA*  *GG* *GA* *AA*

4428 (98.64) 61 (1.36) 0 (0) 1.0000 8978 (98.79) 110 (1.21) 0 (0) 1.0000

rs10000692 T/C (K99E) *TT* *TC* *CC*  *TT* *TC* *CC*

4463 (99.42) 26 (0.58) 0 (0) 1.0000 9042 (99.49) 46 (0.51) 0 (0) 1.0000

rs2278857 T/C *TT* *TC* *CC*  *TT* *TC* *CC*

1310 (29.19) 2206 (49.15) 972 (21.66) 0.4529 2730 (30.05) 4437 (48.83) 1919 (21.12) 0.1388

rs72655988 G/A (A568T) *GG* *GA* *AA*  *GG* *GA* *AA*

4433 (98.75) 55 (1.23) 1 (0.02) 0.1638 8958 (98.57) 129 (1.42) 1 (0.01) 0.3762

rs2276724 T/C (S491G) *TT* *TC* *CC*  *TT* *TC* *CC*

2317 (51.62) 1797 (40.03) 375 (8.35) 0.3214 4819 (53.03) 3606 (39.68) 663 (7.29) 0.7704

rs1549579 T/G *TT* *TG* *GG*  *TT* *TG* *GG*

1729 (38.52) 2112 (47.05) 648 (14.43) 0.9495 3650 (40.17) 4169 (45.88) 1267 (13.95) 0.1693

rs495089 T/C *TT* *TC* *CC*  *TT* *TC* *CC*

1450 (32.30) 2142 (47.72) 897 (19.98) 0.0393 3304 (36.36) 4228 (46.52) 1556 (17.12) 0.0013

rs1801232 G/T (N3552K) *GG* *GT* *TT*  *GG* *GT* *TT*

3691 (82.24) 754 (16.80) 43 (0.96) 0.4824 7567 (83.28) 1440 (15.85) 79 (0.87) 0.2657

rs151057154 G/C (E67Q) *GG* *GC* *CC*  *GG* *GC* *CC*

4357 (99.61) 17 (0.39) 0 (0) 1.0000 9054 (99.63) 34 (0.37) 0 (0) 1.0000

rs59178195 T/G (Q150P) *TT* *TG* *GG*  *TT* *TG* *GG*

4210 (93.79) 270 (6.01) 9 (0.20) 0.0484 8566 (94.35) 506 (5.57) 7 (0.08) 1.0000

rs79060400 T/C *TT* *TC* *CC*  *TT* *TC* *CC*

2806 (74.31) 894 (23.68) 76 (2.01) 0.6330 5603 (74.69) 1748 (23.30) 151 (2.01) 0.2817

rs5770917 T/C *TT* *TC* *CC*  *TT* *TC* *CC*

2886 (64.30) 1411 (31.44) 191 (4.26) 0.2626 5899 (64.91) 2822 (31.05) 367 (4.04) 0.2053

rs138281407 C/G (P130R) *CC* *CG* *GG*  *CC* *CG* *GG*

4453 (99.20) 36 (0.80) 0 (0) 1.0000 9027 (99.33) 60 (0.66) 1 (0.01) 0.0991

rs2070203 G/A *GG* *GA* *AA*  *GG* *GA* *AA*

1198 (26.70) 2228 (49.65) 1061 (23.65) 0.6976 2431 (26.75) 4467 (49.16) 2189 (24.09) 0.1254

rs4275849 G/A *GG* *GA* *AA*  *GG* *GA* *AA*

1129 (25.15) 2238 (49.86) 1122 (24.99) 0.8579 2416 (26.59) 4497 (49.49) 2174 (23.92) 0.3666

rs3171927 A/G *AA* *AG* *GG*  *AA* *AG* *GG*

2409 (53.67) 1767 (39.36) 313 (6.97) 0.6746 4876 (53.66) 3516 (38.70) 694 (7.64) 0.0884

rs112619503 C/T (R165W) *CC* *CT* *TT*  *CC* *CT* *TT*

4408 (98.20) 80 (1.78) 1 (0.02) 0.3115 8967 (98.67) 120 (1.32) 1 (0.01) 0.3356

rs3108919 T/C *TT* *TC* *CC*  *TT* *TC* *CC*

1209 (26.94) 2226 (49.60) 1053 (23.46) 0.6539 2319 (25.52) 4549 (50.07) 2218 (24.41) 0.8998

rs1412115 G/A *GG* *GA* *AA*  *GG* *GA* *AA*

1219 (27.87) 2134 (48.79) 1021 (23.34) 0.1458 2432 (26.76) 4527 (49.81) 2129 (23.43) 0.8010

rs2269702 A/G *AA* *AG* *GG*  *AA* *AG* *GG*

2890 (64.39) 1419 (31.62) 179 (3.99) 0.7779 6250 (68.79) 2551 (28.07) 285 (3.14) 0.2100

rs35999669 T/G (S265A) *TT* *TG* *GG*  *TT* *TG* *GG*

3561 (79.63) 861 (19.25) 50 (1.12) 0.8761 7296 (80.45) 1662 (18.33) 111 (1.22) 0.1422

rs4711319 G/A *GG* *GA* *AA*  *GG* *GA* *AA*

2130 (49.36) 1743 (40.40) 442 (10.24) 0.0025 4229 (48.03) 3654 (41.50) 922 (10.47) 0.0016

rs2549782 T/G (N392K) *TT* *TG* *GG*  *TT* *TG* *GG*

1236 (27.53) 2226 (49.59) 1027 (22.88) 0.6973 2321 (25.54) 4502 (49.55) 2263 (24.91) 0.3897

rs1805070 A/G (I720V) *AA* *AG* *GG*  *AA* *AG* *GG*

3751 (83.56) 707 (15.75) 31 (0.69) 0.7754 7619 (83.84) 1394 (15.34) 75 (0.82) 0.1999

rs714407 G/A *GG* *GA* *AA*  *GG* *GA* *AA*

1583 (35.26) 2144 (47.76) 762 (16.98) 0.4401 3099 (34.10) 4380 (48.20) 1609 (17.70) 0.3653

rs192084699 G/T *GG* *GT* *TT*  *GG* *GT* *TT*

4425 (98.57) 64 (1.43) 0 (0) 1.0000 8928 (98.24) 157 (1.73) 3 (0.03) 0.0360

rs35928055 A/G (S240G) *AA* *AG* *GG*  *AA* *AG* *GG*

3501 (77.99) 919 (20.47) 69 (1.54) 0.3151 6978 (76.78) 1942 (21.37) 168 (1.85) 0.0167

rs2374563 A/G *AA* *AG* *GG*  *AA* *AG* *GG*

1300 (28.96) 2229 (49.67) 959 (21.37) 0.9521 2668 (29.38) 4427 (48.74) 1987 (21.88) 0.0633

rs55675869 C/T (V33366I) *CC* *CT* *TT*  *CC* *CT* *TT*

3995 (88.99) 474 (10.56) 20 (0.45) 0.1640 7997 (88.00) 1056 (11.62) 35 (0.38) 0.9282

rs1054629 A/T (E270D) *AA* *AT* *TT*  *AA* *AT* *TT*

3540 (78.90) 877 (19.54) 70 (1.56) 0.0741 7140 (78.56) 1830 (20.14) 118 (1.30) 0.9585

rs6566532 T/C *TT* *TC* *CC*  *TT* *TC* *CC*

1327 (29.56) 2156 (48.03) 1006 (22.41) 0.0209 2571 (28.29) 4450 (48.97) 2067 (22.74) 0.0923

rs141645766 T/G (S677A) *TT* *TG* *GG*  *TT* *TG* *GG*

4475 (99.69) 14 (0.31) 0 (0) 1.0000 9073 (99.94) 15 (0.16) 0 (0) 1.0000

rs2523638 G/A *GG* *GA* *AA*  *GG* *GA* *AA*

1522 (34.05) 2125 (47.54) 823 (18.41) 0.0917 3007 (33.21) 4303 (47.52) 1745 (19.27) 0.0036

rs2254067 G/T (G499C) *GG* *GT* *TT*  *GG* *GT* *TT*

2730 (60.82) 1538 (34.26) 221 (4.92) 0.8281 5470 (60.19) 3166 (34.84) 452 (4.97) 0.8563

rs146303784 C/T (M649I) *CC* *CT* *TT*  *CC* *CT* *TT*

4378 (97.53) 111 (2.47) 0 (0) 1.0000 8856 (97.45) 230 (2.53) 2 (0.02) 0.6631

rs34348991 G/A (T1084M) *GG* *GA* *AA*  *GG* *GA* *AA*

3809 (84.85) 644 (14.35) 36 (0.80) 0.1274 7733 (85.09) 1303 (14.34) 52 (0.57) 0.7691

rs12511469 A/T *AA* *AT* *TT*  *AA* *AT* *TT*

1727 (38.49) 2087 (46.51) 673 (15.00) 0.3119 3488 (38.38) 4241 (46.67) 1359 (14.95) 0.2394

rs146406799 C/T (R1717W) *CC* *CT* *TT*  *CC* *CT* *TT*

4429 (98.66) 60 (1.14) 0 (0) 1.0000 8946 (98.44) 141 (1.55) 1 (0.01) 1.0000

rs61742122 G/A *GG* *GA* *AA*  *GG* *GA* *AA*

4473 (99.64) 16 (0.56) 0 (0) 1.0000 9036 (99.43) 51 (0.56) 1 (0.01) 0.0732

rs3742945 C/T (R47Q) *CC* *CT* *TT*  *CC* *CT* *TT*

2843 (63.35) 1446 (32.22) 199 (4.43) 0.3850 5707 (62.81) 2999 (33.01) 380 (4.18) 0.5869

rs2289367 G/A *GG* *GA* *AA*  *GG* *GA* *AA*

3092 (68.88) 1256 (27.98) 141 (3.14) 0.3438 6371 (70.10) 2491 (27.41) 226 (2.49) 0.3538

rs77689730 C/T (L211F) *CC* *CT* *TT*  *CC* *CT* *TT*

2280 (50.79) 1867 (41.59) 342 (7.62) 0.1422 4590 (50.51) 3748 (41.24) 750 (8.25) 0.7207

rs1233399 C/T *CC* *CT* *TT*  *CC* *CT* *TT*

2718 (60.55) 1541 (34.33) 230 (5.12) 0.5464 5115 (56.28) 3401 (37.42) 572 (6.30) 0.8448

rs73996306 G/A (A69V) *GG* *GA* *AA*  *GG* *GA* *AA*

3769 (83.96) 687 (15.30) 33 (0.74) 0.7705 7643 (84.10) 1374 (15.12) 71 (0.78) 0.2728

____________________________________________________________________________________________________________

Data are numbers of subjects (percentages). H-W *P*, *P* value for Hardy-Weinberg equilibrium.
