# Supplementary material for: Identification of eight genetic variants as novel determinants of dyslipidemia in Japanese by exome-wide association studies
Source: Oncotarget. 2017 Apr 17;8(24):38950–61. doi: 10.18632/oncotarget.17159 (PMC5503585; doi:10.18632/oncotarget.17159)
Supplement: Supplementary file 9 [file oncotarget-08-38950-s009.docx]

**Supplementary Table 8.** Relation of SNPs to hypertriglyceridemia as determined by multivariable logistic regression analysis.

_____________________________________________________________________________________________________________________________

SNP Dominant Recessive Additive 1 Additive 2

____________________ ____________________ ____________________ ____________________

*P* OR (95% CI) *P* OR (95% CI) *P* OR (95% CI) *P* OR (95% CI)

_____________________________________________________________________________________________________________________________

rs193164904 A/G (I534V) 0.3350 0.2932 0.2673 0.2934

rs143953605 C/T (V55I) 0.8605 0.3034 0.9048 0.3034

rs499974 G/T 0.1396 0.8877 0.1306 0.5660

rs6814310 C/A 0.0807 0.7452 0.0824 0.2165

rs1280396 G/A (A511T) 0.9755 0.3672 0.8573 0.3743

rs146819332 C/T (V204M) 0.5711 0.4859 0.5535 0.4862

rs200264312 G/A (P284S) 0.8567 0.3331 0.7704 0.3334

rs7808146 A/G 0.1566 0.9007 0.1448 0.3469

rs74810099 T/G (M36R) 0.3002 0.1476 0.1997 0.1510

rs11629205 G/A 0.0795 0.4219 0.0341 0.92 (0.85-0.99) 0.9236

rs201278290 G/A (R1921W) 0.9499 ND 0.9499 ND

rs3829251 G/A 0.9125 0.0661 0.6047 0.1331

rs10789907 A/C 0.2893 0.7340 0.3155 0.5032

rs10191097 T/G 0.1099 0.9640 0.0960 0.6364

rs4660080 A/G 0.4011 0.6325 0.4709 0.4363

rs10952789 C/A 0.5660 0.1064 0.2457 0.2731

rs175080 G/A (P844L) 0.6474 0.4356 0.7916 0.4214

rs36117715 G/A (P2218L) 0.5519 ND 0.5519 ND

rs1462978 A/G 0.4257 0.4686 0.5185 0.4444

rs6795970 G/A (A1073V) 0.6814 0.4167 0.5196 0.4530

rs550404 T/C 0.6548 0.2570 0.9377 0.2936

rs2475335 T/C 0.8583 0.6377 0.7437 0.6849

rs10790162 G/A **<1.0 × 10^-23^** 1.48 (1.38-1.59) **1.55 × 10^-12^** 1.62 (1.42-1.85) **5.86 × 10^-19^** 1.41 (1.31-1.53) **5.22 × 10^-19^** 1.87 (1.63-2.15)

rs7350481 C/T **<1.0 × 10^-23^** 1.50 (1.40-1.61) **9.45 × 10^-11^** 1.54 (1.35-1.75) **1.62 × 10^-21^** 1.45 (1.34-1.56) **6.31 × 10^-18^** 1.81 (1.58-2.07)

rs182417021 C/T (E236K) 0.1442 ND 0.1442 ND

rs1541160 A/G 0.5848 0.3533 0.6512 0.3532

rs117922332 T/G (K852N) 0.3895 0.3534 0.3017 0.3687

rs3827047 C/A (E390D) 0.1859 0.7545 0.1986 0.7022

rs145716748 A/G (S729P) 0.9602 0.0298 <0.01 (ND) 0.8195 0.0299 <0.01 (0-0.78)

rs2010834 A/C (F254C) 0.8154 0.9158 0.8322 0.8848

rs12898111 A/G 0.5515 0.8834 0.4950 0.8553

rs1289658 A/G (M496T) 0.0050 1.12 (1.03-1.21) 0.7295 0.0049 1.12 (1.04-1.21) 0.5081

rs2236133 A/G 0.8857 0.3542 0.6485 0.4400

rs10781500 C/T 0.8234 0.6616 0.9305 0.6575

rs586088 A/T (T190S) 0.7309 0.4198 0.9256 0.4243

rs143833298 G/A (R830Q) 0.8272 0.4511 0.7999 0.4512

rs11180311 A/G 0.5488 0.8290 0.5756 0.6785

rs200330080 C/T (R654Q) 0.8666 0.2003 0.9737 0.2002

rs147241730 T/C (N1383S) 0.0868 ND 0.0868 ND

rs75146235 C/G (Q126E) 0.7391 0.1714 0.7978 0.1713

rs2966332 T/C (M174T) 0.0011 0.89 (0.83-0.95) 0.2042 0.0025 0.89 (0.83-0.96) 0.0499 0.87 (0.76-1.00)

rs138329346 C/T (H313Y) 0.2401 0.1411 0.1752 0.1431

rs117009784 A/C (R96S) 0.5266 0.9302 0.5113 0.9409

rs11645831 G/A 0.8065 0.9093 0.8261 0.8397

rs3806932 A/G 0.7609 0.3621 0.9876 0.3787

rs3013105 C/T (E292K) 0.4084 0.8942 0.4068 0.6230

rs35508906 G/A (A584T) 0.1829 ND 0.1829 ND

rs2672785 G/A (G34E) 0.3929 0.4704 0.2478 0.9901

rs1047991 G/A (R162C) 0.7652 0.2642 0.9734 0.2763

rs138686208 G/A (E35K) 0.9894 ND 0.9894 ND

rs4331426 A/G 0.9720 0.0551 0.7769 0.0553

rs1585440 C/A 0.7481 0.5027 0.5770 0.6098

rs200117745 G/C (E16D) 0.2864 0.7694 0.3001 0.7686

rs4867100 C/T 0.1271 0.4712 0.1649 0.3954

rs12126589 A/G 0.9271 0.6866 0.9687 0.7089

rs10951936 A/T 0.5196 0.9108 0.5235 0.8349

rs1959607 T/C 0.9723 0.6062 0.9330 0.6064

rs11751697 C/T 0.0503 0.4266 0.0710 0.2771

rs3752087 G/A (V38M) 0.3818 0.8366 0.3926 0.5495

rs991258 G/C 0.7829 0.3298 0.9748 0.3550

rs200587171 C/T 0.0177 0.71 (0.53-0.94) ND 0.0177 0.71 (0.53-0.94) ND

rs2246901 A/C (S4821A) 0.8222 0.3616 0.9874 0.3660

rs6503018 A/G (M598V) 0.6194 0.0022 1.21 (1.07-1.37) 0.6262 0.0049 1.20 (1.06-1.37)

rs143803280 G/A 0.9511 ND 0.9511 ND

rs11545763 A/G (E297G) 0.8376 0.9698 0.8304 0.9732

rs1277207 T/C (N61S) 0.8930 0.7946 0.9345 0.7922

rs4489954 T/G 0.2502 0.3148 0.3738 0.2389

rs7011881 C/A 0.0667 0.3499 0.0316 0.92 (0.84-0.99) 0.4901

rs1892172 G/A 0.2122 0.1857 0.3929 0.1152

rs1592404 C/G 0.1378 0.1742 0.2527 0.1315

rs3130171 T/C 0.4750 0.8524 0.4901 0.7467

rs144509002 A/G (D336G) 0.2633 0.0830 0.3953 0.0812

rs74491133 C/T 0.1493 0.2079 0.1701 0.2072

_____________________________________________________________________________________________________________________________

Multivariable logistic regression analysis was performed with adjustment for age and sex. Based on Bonferroni’s correction, *P* values of <1.71 × 10^–4^ (0.05/292) were considered statistically significant and are shown in bold. OR, odds ratio; CI, confidence interval; ND, not determined.
