# Supplementary material for: Identification of eight genetic variants as novel determinants of dyslipidemia in Japanese by exome-wide association studies
Source: Oncotarget. 2017 Apr 17;8(24):38950–61. doi: 10.18632/oncotarget.17159 (PMC5503585; doi:10.18632/oncotarget.17159)
Supplement: Supplementary file 10 [file oncotarget-08-38950-s010.docx]

**Supplementary Table 9.** Relation of SNPs to hypo–HDL-cholesterolemia as determined by multivariable logistic regression analysis.

_____________________________________________________________________________________________________________________________

Dominant Recessive Additive 1 Additive 2

SNP ____________________ ____________________ ______________________ ____________________

*P* OR (95% CI) *P* OR (95% CI) *P* OR (95% CI) *P* OR (95% CI)

_____________________________________________________________________________________________________________________________

rs2561111 G/A (R111H) 0.9531 0.8505 0.9888 0.8501

rs3745486 T/C (L99P) 0.4588 0.7891 0.3797 0.8952

rs77885682 C/T (R503K) 0.6382 0.6382 0.5789 0.6449

rs61748749 T/G (S1353R) 0.6984 0.0277 <0.01 (0-0.77) 0.5417 0.0279 <0.01 (ND)

rs143822500 T/C (T867A) 0.1216 0.5736 0.1281 0.5729

rs857591 G/T 0.1211 0.5024 0.0616 0.8681

rs499974 G/T 0.4538 0.1010 0.8191 0.1022

rs184499441 C/T (G1288R) 0.8206 0.6765 0.8513 0.6761

rs1053966 C/G (H1751D) 0.7904 0.4684 0.6345 0.5073

rs12632942 A/G (L1092P) 0.0836 0.1220 0.1973 0.0460 1.14 (1.00-1.29)

rs150294461 G/A (G654E) 0.5212 0.5987 0.5328 0.5983

rs3750208 G/A (R168W) 0.4905 0.9414 0.4684 0.9757

rs2255317 C/T (T855M) 0.3206 0.8722 0.2849 0.9501

rs145716748 A/G (S729P) 0.7076 0.1401 0.6272 0.1405

rs10191097 T/G 0.4762 0.4197 0.6254 0.3654

rs148320716 C/T (R96C) 0.2061 0.7401 0.2167 0.7341

rs2153157 T/C 0.2411 0.4482 0.3233 0.3193

rs7299095 G/A 0.0914 0.3756 0.1395 0.1539

rs17852959 C/T (V161M) 0.7487 0.4893 0.8825 0.4820

rs11629205 G/A 0.7052 0.3588 0.4777 0.5195

rs3729740 C/T (D578N) 0.3672 0.1522 0.6116 0.1330

rs150534954 C/G (C155W) 0.9140 0.1244 0.7534 0.1247

rs11180311 A/G 0.3359 0.4561 0.2012 0.8439

rs11877062 T/C (W4R) 0.9828 0.9109 0.9503 0.9495

rs3747203 T/C (R199G) 0.1089 0.1612 0.0388 0.90 (0.82-0.99) 0.2713

rs78002652 G/A (G139R) 0.8867 0.3848 0.9592 0.3848

rs151000241 T/C (N190S) 0.6948 ND 0.6948 ND

rs6847454 A/T (Q453L) 0.2993 0.8882 0.2583 0.9235

rs143833298 G/A (R830Q) 03784 0.7012 0.3715 0.7015

rs200295807 G/A (R26C) 0.6585 ND 0.6585 ND

rs199606102 C/T (V695I) 0.9726 0.1358 0.8307 0.1359

rs10100935 C/T (A118T) 0.9878 0.3161 0.7476 0.4068

rs586088 A/T (T190S) 0.0383 1.10 (1.01-1.20) 0.2624 0.0703 0.1088

rs146515657 T/C (N650S) **<1.0 × 10^-23^** 35.37 (15.61-95.21) ND **<1.0 × 10^-23^** 35.37 (15.61-95.21) ND

rs4807160 C/T (E144K) 0.6977 0.6926 0.7812 0.6452

rs4838865 G/A (S567L) 0.3798 0.8569 0.3891 0.7824

rs17053501 C/T 0.9805 0.3461 0.9493 0.3461

rs146600946 G/A (R286H) 0.4990 0.9893 0.4960 0.9914

rs143827332 G/A (R1035W) 0.6707 0.3093 0.5778 0.3097

rs1823068 A/G 0.9344 0.5947 0.9438 0.6075

rs7997737 G/A 0.5003 0.9796 0.4805 0.7108

rs9901755 A/G 0.4186 0.6808 0.4769 0.4749

rs150412190 G/A (S116L) 0.8039 ND 0.8039 ND

rs7004867 C/T (R31Q) 0.3473 ND 0.3473 ND

rs2271251 C/G (A82G) 0.9512 0.2666 0.8753 0.2667

rs62623665 G/A (R352H) 0.2699 ND 0.2699 ND

rs57075420 C/T (R297C) 0.2620 0.3593 0.3066 0.3570

rs7442317 G/A 0.0584 0.3014 0.1004 0.1092

rs3820678 G/A (A191T) 0.2228 0.8654 0.1866 0.9717

rs10794531 C/T (R53H) 0.9134 0.8930 0.9457 0.8805

rs78943519 G/A (G15D) 0.6126 0.8494 0.5550 0.9534

rs149721746 A/G (Y60C) 0.0975 0.4238 0.1061 0.4228

rs7619670 G/A 0.6805 0.3799 0.4899 0.4369

rs199763816 G/A (P1116S) 0.3580 ND 0.3580 ND

rs1555494 A/G 0.5171 ND 0.5171 ND

rs2049805 C/T 0.9255 0.5391 0.9379 0.5482

rs11247229 C/T 0.4974 0.1339 0.8255 0.1361

rs6598858 C/T 0.0273 1.11 (1.01-1.21) 0.0286 1.27 (1.03-1.55) 0.0995 0.0163 1.30 (1.05-1.60)

rs147317864 C/T (A262T) **2.26 × 10^-14^** >100 (ND) ND **2.26 × 10^-14^** >100 (ND) ND

rs118174683 G/A (T482M) 0.9600 ND 0.9600 ND

rs1536690 C/T (P72L) 0.8032 0.6936 0.7391 0.7029

rs17316633 G/A 0.7475 0.0261 0.55 (0.30-0.93) 0.9248 0.0265 0.55 (0.30-0.94)

rs139476663 T/C (V87A) 0.8303 0.1246 0.9623 0.1246

rs8059612 G/A 0.2872 0.8683 0.2847 0.5471

rs2032794 A/G 0.5960 0.2008 0.8680 0.1976

rs992822 G/A (S910N) 0.5875 0.0118 1.14 (1.03-1.27) 0.7457 0.0519

rs17028450 C/T (R690C) 0.9932 0.4173 0.9741 0.4173

rs1980889 A/G 0.6325 0.5877 0.4860 0.8123

rs1464890 C/T (A271T) 0.6027 0.8053 0.5326 0.9236

rs2324027 C/T 0.9816 0.7486 0.8889 0.8486

rs7667636 G/A 0.4069 0.3292 0.5949 0.2691

rs10943613 T/C 0.6635 0.9616 0.6576 0.8437

rs948962 C/A (L1954I) 0.3461 0.3377 0.5010 0.2691

rs76022391 G/A (G668S) 0.2142 0.1950 0.2729 0.1925

rs7913069 C/T 0.3430 0.0548 0.5791 0.0525

rs200330080 C/T (R654Q) 0.1082 0.8166 0.1016 0.8208

rs111765932 T/C (I926T) 0.4131 ND 0.4131 ND

rs141510612 G/A (R690Q) 0.6836 ND 0.6836 ND

rs74844425 T/C (I15V) 0.4945 0.4618 0.5245 0.4615

rs12229654 T/G **5.45 × 10^-9^** 1.30 (1.19-1.42) 0.0113 1.28 (1.06-1.53) **9.89 × 10^-8^** 1.29 (1.17-1.41) 0.0005 1.41 (1.16-1.70)

rs145141779 G/A (P337L) 0.3142 0.7293 0.3303 0.7236

rs13188074 A/G 0.1881 0.1524 0.3627 0.1029

rs56369596 G/C (V699L) 0.8453 0.0274 <0.01 (ND) 0.9874 0.0274 <0.01 (ND)

rs874478 G/A 0.0672 0.6738 0.0739 0.4375

rs202021460 C/T (A252T) 0.1400 ND 0.1400 ND

rs179075 T/C 0.9440 0.4541 0.7447 0.5465

rs6699355 C/T 0.3181 0.2302 0.1478 0.4863

rs874889 A/C (I267M) 0.1341 0.8018 0.1337 0.3130

_____________________________________________________________________________________________________________________________

Multivariable logistic regression analysis was performed with adjustment for age and sex. Based on Bonferroni’s correction, *P* values of <1.44 × 10^–4^ (0.05/348) were considered statistically significant and are shown in bold. OR, odds ratio; CI, confidence interval; ND, not determined.
