# Supplementary material for: Identification of eight genetic variants as novel determinants of dyslipidemia in Japanese by exome-wide association studies
Source: Oncotarget. 2017 Apr 17;8(24):38950–61. doi: 10.18632/oncotarget.17159 (PMC5503585; doi:10.18632/oncotarget.17159)
Supplement: Supplementary file 11 [file oncotarget-08-38950-s011.docx]

**Supplementary Table 10.** Relation of SNPs to hyper–LDL-cholesterolemia as determined by multivariable logistic regression analysis.

_____________________________________________________________________________________________________________________________

SNP Dominant Recessive Additive 1 Additive 2

____________________ ____________________ ____________________ ____________________

*P* OR (95% CI) *P* OR (95% CI) *P* OR (95% CI) *P* OR (95% CI)

_____________________________________________________________________________________________________________________________

rs2125904 C/A 0.8256 0.0645 0.4138 0.1061

rs2959953 C/G (P213R) 0.0104 1.11 (1.03-1.21) 0.4440 0.0144 1.11 (1.02-1.22) 0.0456 1.11 (1.00-1.23)

rs12144325 A/G (L366P) 0.9767 0.7467 0.9449 0.7471

rs2496425 T/C (F1070S) 0.1591 0.7068 0.1741 0.3786

rs10097386 T/C 0.7535 0.0551 0.3118 0.2421

rs7120775 C/G (Y27*) 0.8472 0.8060 0.8946 0.7960

rs138559558 G/A (R289C) 0.4930 0.2072 0.5413 0.2068

rs7532317 C/A 0.2279 0.5529 0.1368 0.9803

rs4132509 C/A 0.9698 0.3509 0.7374 0.4078

rs5716 G/C (K367N) 0.8121 0.1177 0.8630 0.1247

rs7771335 A/G **8.86 × 10^-12^** 1.29 (1.20-1.39) **7.50 × 10^-7^** 1.45 (1.26-1.68) **2.48 × 10^-8^** 1.24 (1.15-1.34) **3.81 × 10^-9^** 1.58 (1.36-1.83)

rs79980197 C/G (P706R) 0.0591 0.4285 0.0723 0.4203

rs201078160 C/T (R4162Q) 0.1014 ND 0.1014 ND

rs10770 T/C (I180T) 0.8784 0.0184 0.64 (0.43-0.93) 0.7193 0.0194 0.64 (0.43-0.93)

rs76974938 C/T (D67N) **8.58 × 10^-13^** 0.48 (0.39-0.59) ND **8.58 × 10^-13^** 0.48 (0.39-0.59) ND

rs76828246 G/A (S277N) 0.3056 ND 0.3056 ND

rs2071653 C/T **8.58 × 10^-9^** 1.23 (1.15-1.33) **7.00 × 10^-8^** 1.42 (1.25-1.61) **2.20 × 10^-5^** 1.18 (1.09-1.27) **4.26 × 10^-10^** 1.52 (1.34-1.74)

rs10778257 T/G (E11D) 0.6231 0.5400 0.7639 0.5002

rs6901 C/T (R1038Q) 0.1103 0.5468 0.1372 0.3855

rs200400344 G/A (R366Q) 0.2325 0.3968 0.2095 0.3972

rs7793970 G/A 0.3963 0.5499 0.4923 0.3784

rs56133554 T/G (T1077P) 0.5263 0.9643 0.5266 0.9593

rs150412190 G/A (S116L) 0.7424 ND 0.7424 ND

rs3797036 A/C (N456K) 0.1778 0.6940 0.1914 0.6885

rs60312980 G/T 0.0478 1.15 (1.00-1.32) 0.6139 0.0392 1.16 (1.01-1.33) 0.6282

rs146243553 A/G (S67P) 0.8215 0.3456 0.7704 0.3457

rs1757106 T/G 0.0130 1.10 (1.02-1.19) 0.0338 1.11 (1.01-1.21) 0.0592 0.0061 1.16 (1.04-1.29)

rs117135042 C/T (S634L) 0.7247 0.1705 0.8605 0.1700

rs1292053 G/A (T76M) 0.2934 0.9646 0.2587 0.6559

rs151252589 T/G (F327L) 0.7843 0.1209 0.7000 0.1211

rs11101224 G/A (T428M) 0.8025 0.0060 1.81 (1.19-2.74) 0.4156 0.0066 1.79 (1.18-2.72)

rs2853969 C/T **2.11 × 10^-10^** 1.34 (1.22-1.46) 0.0199 1.45 (1.06-1.96) **2.31 × 10^-9^** 1.33 (1.21-1.45) 0.0080 1.53 (1.12-2.07)

rs2835655 G/A 0.7510 0.2704 0.9544 0.3732

rs202169174 T/C (D1528G) 0.8559 ND 0.8559 ND

rs151324745 A/C (Y1136D) 0.9695 0.3911 0.9993 0.3911

rs2395402 T/C 0.0023 1.13 (1.04-1.21) 0.0614 0.0085 1.11 (1.03-1.20) 0.0290 1.24 (1.02-1.50)

rs783540 G/A 0.5089 0.5354 0.6327 0.4343

rs147989324 G/A (V304I) 0.9321 ND 0.9321 ND

rs11543598 G/A (P213L) 0.3723 0.9955 0.3710 0.9935

rs7124275 T/C 0.4212 0.5496 0.2872 0.9852

rs6801425 A/G 0.1667 0.3871 0.2453 0.2047

rs200636353 G/A (G34S) 0.2382 0.1412 0.2989 0.1410

rs3746875 A/C (M30L) 0.9082 0.2152 0.7936 0.2154

rs1007160 G/T (L223M) 0.9721 0.3903 0.7442 0.4640

rs7305779 A/C (E103A) 0.7296 0.4710 0.8459 0.4658

rs17232910 G/C (A643P) 0.2357 0.2606 0.3361 0.2411

rs1152522 C/T 0.1342 0.9530 0.1311 0.9342

rs61308377 A/G (Y209H) 0.2949 0.9923 0.2747 0.8711

rs144335584 A/G (I747V) 0.1441 0.9630 0.1404 0.9653

rs3775948 G/C 0.2397 0.1661 0.4490 0.1146

rs12669721 G/T (P119T) 0.8234 0.7174 0.7341 0.7623

rs3944066 C/T (P494L) 0.9131 0.4660 0.9845 0.4660

rs144134358 C/T (S569L) 0.0896 ND 0.0896 ND

rs199905767 C/A (P620T) 0.2658 ND 0.2658 ND

rs41284134 G/C (G245R) 0.3096 0.9651 0.3093 0.9633

rs201453898 C/T (R547Q) 0.3922 ND 0.3922 ND

rs2269704 C/T **3.83 × 10^-9^** 1.28 (1.18-1.39) 0.1776 **9.70 × 10^-9^** 1.28 (1.18-1.39) 0.0707

rs115387731 C/T (V8I) 0.2844 ND 0.2844 ND

rs2269703 G/A **4.42 × 10^-9^** 1.28 (1.18-1.39) 0.1911 **1.05 × 10^-8^** 1.28 (1.18-1.39) 0.0773

rs1264318 G/C 0.3369 0.2871 0.2510 0.2980

rs10917536 G/T (Q72K) 0.3304 0.7598 0.2565 0.8814

rs148768286 C/T (V266I) 0.4847 ND 0.4847 ND

rs7442317 G/A 0.1807 0.3235 0.2835 0.1834

rs12069239 G/C (A471P) 0.2482 0.4800 0.1690 0.5680

rs9469042 T/C 0.1119 0.2902 0.0675 0.3141

rs618662 C/A 0.2837 0.8723 0.2389 0.8732

rs8133766 T/C 0.1606 0.9631 0.1418 0.5045

rs117778870 G/A (R40H) 0.5531 0.7237 0.5731 0.7224

rs140750531 G/A (R551H) 0.0883 0.6130 0.0980 0.6078

rs4953863 C/T 0.7929 0.1207 0.4057 0.4118

rs74546291 G/A (W240*) 0.4232 ND 0.4232 ND

rs10000692 T/C (K99E) 0.6176 ND 0.6176 ND

rs2278857 T/C 0.2883 0.4985 0.3762 0.3012

rs72655988 G/A (A568T) 0.3378 0.6385 0.3100 0.6394

rs2276724 T/C (S491G) 0.1306 0.0318 1.16 (1.01-1.32) 0.3674 0.0215 1.17 (1.02-1.34)

rs1549579 T/G 0.0762 0.4411 0.1075 0.1863

rs495089 T/C **2.90 × 10^-6^** 1.20 (1.11-1.29) **4.45 × 10^-5^** 1.21 (1.11-1.33) 0.0005 1.15 (1.06-1.25) **1.87 × 10^-7^** 1.32 (1.19-1.46)

rs1801232 G/T (N3552K) 0.1436 0.6529 0.1618 0.6111

rs151057154 G/C (E67Q) 0.9292 ND 0.9292 ND

rs59178195 T/G (Q150P) 0.1815 0.0695 0.2765 0.0680

rs79060400 T/C 0.7532 0.8900 0.7193 0.9130

rs5770917 T/C 0.5155 0.5471 0.6161 0.5052

rs138281407 C/G (P130R) 0.4273 0.3611 0.3842 0.3614

rs2070203 G/A 0.9891 0.5391 0.8366 0.6912

rs4275849 G/A 0.0676 0.1690 0.1471 0.0508

rs3171927 A/G 0.9394 0.1755 0.6098 0.2281

rs112619503 C/T (R165W) 0.0395 1.35 (1.01-1.79) 0.6179 0.0434 1.35 (1.01-1.79) 0.6155

rs3108919 T/C 0.0784 0.2013 0.1573 0.0642

rs1412115 G/A 0.1913 0.9555 0.1712 0.4277

rs2269702 A/G **2.74 × 10^-7^** 1.22 (1.13-1.32) 0.0147 1.27 (1.05-1.54) **3.25 × 10^-6^** 1.21 (1.11-1.30) 0.0027 1.35 (1.11-1.63)

rs35999669 T/G (S265A) 0.2543 0.6522 0.2044 0.7012

rs4711319 G/A 0.1303 0.6788 0.1449 0.4175

rs2549782 T/G (N392K) 0.0178 0.91 (0.84-0.98) 0.0089 0.89 (0.82-0.97) 0.1141 0.0023 0.85 (0.77-0.95)

rs1805070 A/G (I720V) 0.6224 0.4053 0.5018 0.4200

rs714407 G/A 0.2130 0.3003 0.3399 0.1769

rs192084699 G/T 0.1571 0.1364 0.1973 0.1359

rs35928055 A/G (S240G) 0.1198 0.1849 0.2037 0.1588

rs2374563 A/G 0.6103 0.5188 0.4397 0.8784

rs55675869 C/T (V33366I) 0.0989 0.5555 0.0746 0.5832

rs1054629 A/T (E270D) 0.6121 0.2423 0.4251 0.2629

rs6566532 T/C 0.1304 0.6801 0.1443 0.2638

rs141645766 T/G (S677A) 0.0814 ND 0.0814 ND

rs2523638 G/A 0.3793 0.2764 0.5901 0.2246

rs2254067 G/T (G499C) 0.5594 0.9497 0.5553 0.8728

rs146303784 C/T (M649I) 0.7170 0.2154 0.7713 0.2152

rs34348991 G/A (T1084M) 0.7327 0.1178 0.9708 0.1177

rs12511469 A/T 0.9191 0.9170 0.8853 0.9686

rs146406799 C/T (R1717W) 0.3225 0.3622 0.3471 0.3618

rs61742122 G/A 0.0876 0.3867 0.1021 0.3863

rs3742945 C/T (R47Q) 0.5959 0.4695 0.4473 0.5464

rs2289367 G/A 0.1559 0.0298 1.27 (1.02-1.57) 0.3712 0.0242 1.28 (1.03-1.59)

rs77689730 C/T (L211F) 0.7159 0.1805 0.9705 0.1976

rs1233399 C/T **3.11 × 10^-6^** 0.84 (0.78-0.90) 0.0063 0.81 (0.69-0.94) **5.29 × 10^-5^** 0.85 (0.79-0.92) 0.0006 0.76 (0.65-0.89)

rs73996306 G/A (A69V) 0.8008 0.7254 0.7416 0.7347

_____________________________________________________________________________________________________________________________

Multivariable logistic regression analysis was performed with adjustment for age and sex. Based on Bonferroni’s correction, *P* values of <1.10 × 10^–4^ (0.05/456) were considered statistically significant and are shown in bold. OR, odds ratio; CI, confidence interval; ND, not determined.
