# Supplementary material for: Identification of eight genetic variants as novel determinants of dyslipidemia in Japanese by exome-wide association studies
Source: Oncotarget. 2017 Apr 17;8(24):38950–61. doi: 10.18632/oncotarget.17159 (PMC5503585; doi:10.18632/oncotarget.17159)
Supplement: Supplementary file 12 [file oncotarget-08-38950-s012.docx]

**Supplementary Table 13.** Relation of SNPs to the serum concentration of triglycerides.

_________________________________________________________________________

SNP Serum triglycerides (mmol/L) *P*

_________________________________________________________________________

Associated with serum triglycerides and hypertriglyceridemia

rs10790162 G/A *GG* *GA* *AA*

1.35 ± 0.91 1.50 ± 1.06 1.68 ± 1.16 **<1.0 × 10^-23^**

rs7350481 C/T *CC* *CT* *TT*

1.33 ± 0.86 1.52 ± 1.10 1.65 ± 1.14 **<1.0 × 10^-23^**

Associated with serum triglycerides

rs2075291 C/A (G185C) *CC* *CA* *AA*

1.38 ± 0.91 1.71 ± 1.31 2.52 ± 2.11 **<1.0 × 10^-23^**

rs2266788 T/C *TT* *TC* *CC*

1.34 ± 0.91 1.51 ± 1.06 1.68 ± 1.16 **<1.0 × 10^-23^**

rs964184 C/G *CC* *CG* *GG*

1.34 ± 0.91 1.51 ± 1.06 1.68 ± 1.17 **<1.0 × 10^-23^**

rs9326246 G/C *GG* *GC* *CC*

1.35 ± 0.91 1.50 ± 1.06 1.67 ± 1.14 **<1.0 × 10^-23^**

rs2075290 T/C *TT* *TC* *CC*

1.35 ± 0.91 1.50 ± 1.06 1.66 ± 1.15 **<1.0 × 10^-23^**

rs143974258 G/A (R360*) *GG* *GA*

1.42 ± 1.02 1.77 ± 1.06 **2.10 × 10^-19^**

rs5104 T/C (N147S) *TT* *TC* *CC*

1.36 ± 0.90 1.46 ± 1.02 1.54 ± 1.15 **2.63 × 10^-13^**

rs76974938 C/T (D67N) *CC* *CT*

1.42 ± 1.02 1.77 ± 1.04 **4.74 × 10^-14^**

rs328 C/G (S474*) *CC* *CG* *GG*

1.47 ± 1.01 1.32 ± 0.92 1.36 ± 1.19 **2.79 × 10^-13^**

rs10096633 C/T *CC* *CT* *TT*

1.47 ± 1.01 1.32 ± 0.92 1.36 ± 1.20 **5.11 × 10^-13^**

rs17482753 G/T *GG* *GT* *TT*

1.47 ± 1.01 1.32 ± 0.92 1.36 ± 1.21 **4.70 × 10^-13^**

rs12678919 A/G *AA* *AG* *GG*

1.47 ± 1.01 1.31 ± 0.92 1.37 ± 1.22 **3.87 × 10^-13^**

rs10503669 C/A *CC* *CA* *AA*

1.47 ± 1.01 1.32 ± 0.92 1.37 ± 1.22 **4.17 × 10^-13^**

rs2075292 G/T *GG* *GT* *TT*

1.49 ± 1.08 1.45 ± 0.99 1.32 ± 0.86 **8.67 × 10^-13^**

rs1260326 T/C (L446P) *TT* *TC* *CC*

1.50 ± 1.04 1.42 ± 1.01 1.33 ± 0.83 **1.47 × 10^-11^**

rs10047462 G/T *GG* *GT* *TT*

1.49 ± 1.08 1.44 ± 0.99 1.33 ± 0.86 **1.97 × 10^-12^**

rs780093 A/G *AA* *AG* *GG*

1.50 ± 1.03 1.42 ± 1.02 1.33 ± 0.82 **7.40 × 10^-11^**

rs1260333 T/C *TT* *TC* *CC*

1.50 ± 1.03 1.42 ± 1.03 1.33 ± 0.82 **7.88 × 10^-11^**

rs7016880 G/C *GG* *GC* *CC*

1.46 ± 1.00 1.32 ± 0.93 1.37 ± 1.22 **8.83 × 10^-12^**

rs138406927 C/T (A1096T) *CC* *CT*

1.42 ± 1.02 1.74 ± 0.99 **2.66 × 10^-10^**

rs34429135 T/A (F115Y) *TT* *TA*

1.46 ± 1.02 1.20 ± 0.69 **1.24 × 10^-9^**

rs7112513 G/A *GG* *GA* *AA*

1.36 ± 0.94 1.44 ± 1.01 1.50 ± 1.03 **1.65 × 10^-8^**

rs4936367 A/G (V151M) *AA* *AG* *GG*

1.36 ± 0.94 1.44 ± 1.01 1.50 ± 1.03 **1.65 × 10^-8^**

rs12269901 G/C *GG* *GC* *CC*

1.49 ± 1.05 1.41 ± 0.97 1.35 ± 0.90 **1.39 × 10^-8^**

rs1919128 G/A (V774I) *GG* *GA* *AA*

1.50 ± 1.02 1.41 ± 1.01 1.36 ± 0.87 **1.38 × 10^-8^**

rs141569282 G/A (A117T) *GG* *GA*

1.47 ± 1.02 1.19 ± 0.85 **8.08 × 10^-9^**

rs2954038 A/C *AA* *AC* *CC*

1.39 ± 0.93 1.46 ± 1.05 1.54 ± 1.05 **8.43 × 10^-8^**

rs2197089 C/T *CC* *CT* *TT*

1.48 ± 1.03 1.38 ± 0.94 1.37 ± 0.95 **3.27 × 10^-8^**

rs1919127 C/T (A685V) *CC* *CT* *TT*

1.52 ± 1.04 1.44 ± 1.05 1.38 ± 0.90 **2.69 × 10^-7^**

rs1240773 G/T *GG* *GT* *TT*

1.39 ± 0.95 1.45 ± 0.97 1.54 ± 1.29 **3.08 × 10^-7^**

rs15285 G/A *GG* *GA* *AA*

1.47 ± 1.01 1.36 ± 0.94 1.37 ± 1.09 **5.21 × 10^-8^**

rs13702 A/G *AA* *AG* *GG*

1.47 ± 1.01 1.36 ± 0.94 1.37 ± 1.09 **5.14 × 10^-8^**

rs2954026 G/T *GG* *GT* *TT*

1.39 ± 0.93 1.45 ± 1.03 1.52 ± 1.06 **5.81 × 10^-7^**

rs326 A/G *AA* *AG* *GG*

1.47 ± 1.00 1.36 ± 0.96 1.37 ± 1.08 **8.15 × 10^-8^**

rs200982668 G/A (E2501K) *GG* *GA*

1.44 ± 1.00 1.17 ± 0.74 **8.94 × 10^-8^**

rs1264429 A/G *AA* *AG* *GG*

1.41 ± 0.94 1.50 ± 1.09 1.57 ± 1.42 **5.85 × 10^-7^**

rs1441756 T/G *TT* *TG* *GG*

1.46 ± 1.01 1.36 ± 0.94 1.37 ± 1.10 **1.32 × 10^-7^**

rs2083637 T/C *TT* *TC* *CC*

1.46 ± 1.01 1.37 ± 0.94 1.37 ± 1.10 **1.67 × 10^-7^**

rs2954033 G/A *GG* *GA* *AA*

1.39 ± 0.93 1.45 ± 1.03 1.52 ± 1.06 **1.05 × 10^-6^**

rs301 T/C *TT* *TC* *CC*

1.46 ± 1.00 1.37 ± 0.96 1.36 ± 1.09 **2.48 × 10^-7^**

rs78010183 A/T (T1305S) *AA* *AT*

1.44 ± 1.00 1.23 ± 0.78 **5.96 × 10^-7^**

rs61734696 G/T (Q137K) *GG* *GT*

1.44 ± 1.00 1.18 ± 0.75 **7.44 × 10^-7^**

rs4938303 T/C *TT* *TC* *CC*

1.37 ± 0.95 1.45 ± 1.01 1.48 ± 1.02 **2.27 × 10^-6^**

rs4909945 C/T (V11I) *CC* *CT* *TT*

1.43 ± 0.98 1.96 ± 2.13 1.41 ± 0.81 **1.34 × 10^-6^**

_________________________________________________________________________

Data were compared among genotypes by one-way ANOVA. Based on Bonferroni’s correction, *P* values of <0.0011 (0.05/46) were considered statistically significant and are shown in bold.
