# Supplementary material for: Identification of eight genetic variants as novel determinants of dyslipidemia in Japanese by exome-wide association studies
Source: Oncotarget. 2017 Apr 17;8(24):38950–61. doi: 10.18632/oncotarget.17159 (PMC5503585; doi:10.18632/oncotarget.17159)
Supplement: Supplementary file 13 [file oncotarget-08-38950-s013.docx]

**Supplementary Table 14.** Relation of SNPs to the serum concentration of HDL-cholesterol.

_________________________________________________________________________

SNP Serum HDL-cholesterol (mmol/L) *P*

_________________________________________________________________________

Associated with serum HDL-cholesterol and hypo–HDL-cholesterolemia

rs146515657 T/C (N650S) *TT* *TC*

1.50 ± 0.46 1.18 ± 0.49 **1.54 × 10^-10^**

rs12229654 T/G *TT* *TG* *GG*

1.50 ± 0.47 1.45 ± 0.46 1.44 ± 0.44 **5.07 × 10^-9^**

Associated with serum HDL-cholesterol

rs150552771 T/C (K200E) *TT* *TC*

1.50 ± 0.46 1.23 ± 0.47 **<1.0 × 10^-23^**

rs34429135 T/A (F115Y) *TT* *TA*

1.52 ± 0.46 1.19 ± 0.43 **<1.0 × 10^-23^**

rs17115182 G/A (P43S) *GG* *GA*

1.50 ± 0.46 1.31 ± 0.48 **<1.0 × 10^-23^**

rs116417209 G/A (V151I) *GG* *GA*

1.50 ± 0.46 1.22 ± 0.46 **<1.0 × 10^-23^**

rs3764261 G/T *GG* *GT* *TT*

1.44 ± 0.46 1.54 ± 0.48 1.61 ± 0.49 **<1.0 × 10^-23^**

rs247616 C/T *CC* *CT* *TT*

1.44 ± 0.46 1.54 ± 0.48 1.60 ± 0.49 **<1.0 × 10^-23^**

rs9261800 C/G *CC* *CG*

1.50 ± 0.46 1.23 ± 0.45 **<1.0 × 10^-23^**

rs150854849 C/T (R179Q) *CC* *CT*

1.50 ± 0.46 1.22 ± 0.47 **<1.0 × 10^-23^**

rs138533962 G/A (R379C) *GG* *GA*

1.50 ± 0.46 1.21 ± 0.44 **<1.0 × 10^-23^**

rs2303790 A/G (D459G) *AA* *AG* *GG*

1.47 ± 0.46 1.63 ± 0.50 1.98 ± 0.55 **<1.0 × 10^-23^**

rs2075291 C/A (G185C) *CC* *CA* *AA*

1.50 ± 0.47 1.38 ± 0.44 1.22 ± 0.40 **<1.0 × 10^-23^**

rs78010183 A/T (T1305S) *AA* *AT*

1.47 ± 0.47 1.69 ± 0.46 **<1.0 × 10^-23^**

rs1800588 T/C *TT* *TC* *CC*

1.55 ± 0.49 1.47 ± 0.46 1.42 ± 0.50 **<1.0 × 10^-23^**

rs141569282 G/A (A117T) *GG* *GA*

1.44 ± 0.46 1.67 ± 0.42 **<1.0 × 10^-23^**

rs201166643 C/A (R488S) *CC* *CA*

1.49 ± 0.46 1.21 ± 0.42 **<1.0 × 10^-23^**

rs1532624 G/T *GG* *GT* *TT*

1.44 ± 0.46 1.51 ± 0.47 1.55 ± 0.48 **4.00 × 10^-23^**

rs261334 G/C *GG* *GC* *CC*

1.44 ± 0.45 1.48 ± 0.47 1.54 ± 0.49 **9.90 × 10^-22^**

rs11066015 G/A *GG* *GA* *AA*

1.51 ± 0.48 1.45 ± 0.46 1.40 ± 0.43 **3.84 × 10^-21^**

rs671 G/A (E504K) *GG* *GA* *AA*

1.51 ± 0.48 1.45 ± 0.46 1.40 ± 0.43 **8.23 × 10^-21^**

rs173539 C/T *CC* *CT* *TT*

1.44 ± 0.45 1.50 ± 0.48 1.56 ± 0.51 **1.09 × 10^-20^**

rs35874056 G/A (G460S) *GG* *GA*

1.47 ± 0.46 1.21 ± 0.43 **1.12 × 10^-21^**

rs9939224 G/T *GG* *GT* *TT*

1.49 ± 0.47 1.41 ± 0.45 1.35 ± 0.44 **2.77 × 10^-19^**

rs3782886 A/G *AA* *AG* *GG*

1.51 ± 0.48 1.45 ± 0.46 1.41 ± 0.43 **2.96 × 10^-19^**

rs2074356 C/T *CC* *CT* *TT*

1.51 ± 0.48 1.45 ± 0.46 1.40 ± 0.43 **1.60 × 10^-18^**

rs11066280 T/A *TT* *TA* *AA*

1.51 ± 0.48 1.45 ± 0.46 1.41 ± 0.43 **1.65 × 10^-18^**

rs73055442 C/T (R103H) *CC* *CT*

1.50 ± 0.46 1.26 ± 0.49 **5.32 × 10^-19^**

rs200982668 G/A (E2501K) *GG* *GA*

1.47 ± 0.47 1.68 ± 0.45 **7.82 × 10^-18^**

rs199921354 C/T (R80Q) *CC* *CT*

1.47 ± 0.47 1.69 ± 0.45 **8.52 × 10^-18^**

rs61734696 G/T (Q137K) *GG* *GT*

1.47 ± 0.47 1.68 ± 0.45 **1.21 × 10^-17^**

rs143027124 C/T (V213I) *CC* *CT*

1.50 ± 0.46 1.23 ± 0.48 **3.56 × 10^-17^**

rs139537100 C/T (R24Q) *CC* *CT*

1.47 ± 0.47 1.68 ± 0.45 **6.40 × 10^-17^**

rs199576535 G/A (V341I) *GG* *GA*

1.47 ± 0.47 1.70 ± 0.49 **8.37 × 10^-17^**

rs200787930 C/T (E1095K) *CC* *CT*

1.47 ± 0.47 1.68 ± 0.45 **1.18 × 10^-16^**

rs188378669 G/T *GG* *GT*

1.47 ± 0.47 1.67 ± 0.45 **2.08 × 10^-16^**

rs115287176 G/A (R277W) *GG* *GA*

1.47 ± 0.47 1.68 ± 0.44 **4.08 × 10^-16^**

rs192210727 G/T (R580I) *GG* *GT* *TT*

1.47 ± 0.47 1.66 ± 0.44 1.84 ± 0.48 **5.71 × 10^-15^**

rs146879198 G/A (R340*) *GG* *GA*

1.47 ± 0.47 1.67 ± 0.45 **1.07 × 10^-15^**

rs146092501 C/T (E1386K) *CC* *CT*

1.47 ± 0.47 1.67 ± 0.45 **2.87 × 10^-15^**

rs200134435 G/A (R103W) *GG* *GA*

1.50 ± 0.46 1.21 ± 0.40 **4.19 × 10^-15^**

rs609636 G/A (D76N) *GG* *GA*

1.50 ± 0.46 1.33 ± 0.50 **6.39 × 10^-15^**

rs15285 G/A *GG* *GA* *AA*

1.46 ± 0.46 1.52 ± 0.48 1.54 ± 0.47 **6.62 × 10^-13^**

rs13702 A/G *AA* *AG* *GG*

1.46 ± 0.46 1.52 ± 0.48 1.54 ± 0.47 **8.02 × 10^-13^**

rs7499892 C/T *CC* *CT* *TT*

1.50 ± 0.46 1.44 ± 0.47 1.41 ± 0.53 **2.11 × 10^-12^**

rs1800775 A/C *AA* *AC* *CC*

1.52 ± 0.49 1.47 ± 0.46 1.44 ± 0.46 **2.25 × 10^-12^**

rs7773955 C/T *CC* *CT* *TT*

1.51 ± 0.46 1.45 ± 0.47 1.45 ± 0.49 **4.41 × 10^-14^**

rs326 A/G *AA* *AG* *GG*

1.46 ± 0.46 1.52 ± 0.48 1.53 ± 0.47 **1.57 × 10^-12^**

rs2197089 C/T *CC* *CT* *TT*

1.45 ± 0.46 1.50 ± 0.47 1.53 ± 0.47 **5.18 × 10^-12^**

rs2083637 T/C *TT* *TC* *CC*

1.46 ± 0.46 1.52 ± 0.48 1.54 ± 0.48 **5.36 × 10^-12^**

rs301 T/C *TT* *TC* *CC*

1.46 ± 0.46 1.52 ± 0.48 1.53 ± 0.48 **3.52 × 10^-12^**

rs1441756 T/G *TT* *TG* *GG*

1.46 ± 0.46 1.52 ± 0.48 1.54 ± 0.48 **6.07 × 10^-12^**

rs17482753 G/T *GG* *GT* *TT*

1.46 ± 0.46 1.53 ± 0.48 1.53 ± 0.48 **1.06 × 10^-11^**

rs10096633 C/T *CC* *CT* *TT*

1.46 ± 0.46 1.53 ± 0.48 1.53 ± 0.48 **1.43 × 10^-11^**

rs1532085 A/G *AA* *AG* *GG*

1.51 ± 0.47 1.47 ± 0.48 1.43 ± 0.43 **7.59 × 10^-11^**

rs328 C/G (S474*) *CC* *CG* *GG*

1.46 ± 0.46 1.53 ± 0.48 1.53 ± 0.48 **3.32 × 10^-11^**

rs10503669 C/A *CC* *CA* *AA*

1.46 ± 0.46 1.53 ± 0.48 1.53 ± 0.49 **4.62 × 10^-11^**

rs12678919 A/G *AA* *AG* *GG*

1.46 ± 0.46 1.53 ± 0.48 1.53 ± 0.48 **4.96 × 10^-11^**

rs2266788 T/C *TT* *TC* *CC*

1.50 ± 0.47 1.46 ± 0.46 1.43 ± 0.45 **1.60 × 10^-10^**

rs12231744 C/T (R876K) *CC* *CT* *TT*

1.46 ± 0.46 1.48 ± 0.47 1.54 ± 0.49 **1.95 × 10^-10^**

rs10790162 G/A *GG* *GA* *AA*

1.50 ± 0.47 1.46 ± 0.46 1.43 ± 0.45 **3.86 × 10^-10^**

rs147284320 C/T (V503I) *CC* *CT*

1.52 ± 0.47 1.67 ± 0.44 **8.88 × 10^-11^**

rs964184 C/G *CC* *CG* *GG*

1.50 ± 0.47 1.46 ± 0.46 1.43 ± 0.46 **4.38 × 10^-10^**

rs200585398 A/G (M167V) *AA* *AG*

1.50 ± 0.46 1.22 ± 0.42 **1.77 × 10^-10^**

rs1883025 G/A *GG* *GA* *AA*

1.50 ± 0.48 1.46 ± 0.46 1.42 ± 0.44 **1.82 × 10^-9^**

rs7016880 G/C *GG* *GC* *CC*

1.46 ± 0.46 1.53 ± 0.48 1.52 ± 0.47 **3.68 × 10^-10^**

rs7969300 T/C (N248S) *TT* *TC* *CC*

1.45 ± 0.45 1.48 ± 0.47 1.53 ± 0.50 **1.65 × 10^-9^**

rs9326246 G/C *GG* *GC* *CC*

1.50 ± 0.47 1.46 ± 0.46 1.43 ± 0.45 **1.86 × 10^-9^**

rs61733202 G/A (G26R) *GG* *GA*

1.50 ± 0.46 1.06 ± 0.35 **4.21 × 10^-10^**

rs2075290 T/C *TT* *TC* *CC*

1.50 ± 0.47 1.46 ± 0.46 1.43 ± 0.45 **4.01 × 10^-9^**

rs2072134 C/T *CC* *CT* *TT*

1.49 ± 0.48 1.45 ± 0.46 1.42 ± 0.43 **3.01 × 10^-9^**

rs1610640 A/G *AA* *AG* *GG*

1.68 ± 0.44 1.56 ± 0.49 1.55 ± 0.55 **4.67 × 10^-11^**

rs1058026 T/G *TT* *TG* *GG*

1.46 ± 0.46 1.49 ± 0.47 1.53 ± 0.48 **1.48 × 10^-8^**

rs147186786 C/T (R268Q) *CC* *CT*

1.48 ± 0.47 1.79 ± 0.49 **3.46 × 10^-9^**

rs10468017 C/T *CC* *CT* *TT*

1.46 ± 0.46 1.50 ± 0.48 1.54 ± 0.48 **3.44 × 10^-8^**

rs200449136 G/A *GG* *GA*

1.49 ± 0.46 1.17 ± 0.40 **4.79 × 10^-9^**

rs2043085 A/G *AA* *AG* *GG*

1.51 ± 0.47 1.47 ± 0.48 1.44 ± 0.43 **4.65 × 10^-8^**

rs7350481 C/T *CC* *CT* *TT*

1.50 ± 0.47 1.46 ± 0.47 1.44 ± 0.46 **2.88 × 10^-8^**

rs4775041 G/C *GG* *GC* *CC*

1.46 ± 0.46 1.50 ± 0.48 1.54 ± 0.48 **6.53 × 10^-8^**

rs3129945 G/A *GG* *GA* *AA*

1.52 ± 0.46 1.47 ± 0.47 1.48 ± 0.47 **5.26 × 10^-9^**

rs139088883 G/A (A1840V) *GG* *GA*

1.50 ± 0.46 1.21 ± 0.43 **7.62 × 10^-8^**

rs2076528 T/G *TT* *TG* *GG*

1.46 ± 0.46 1.49 ± 0.48 1.55 ± 0.47 **1.84 × 10^-7^**

rs3763315 G/T *GG* *GT* *TT*

1.46 ± 0.46 1.49 ± 0.48 1.55 ± 0.47 **1.96 × 10^-7^**

rs41441651 C/T (D336N) *CC* *CT* *TT*

1.46 ± 0.46 1.49 ± 0.48 1.55 ± 0.47 **1.89 × 10^-7^**

rs28362675 C/A (E454*) *CC* *CA* *AA*

1.46 ± 0.46 1.49 ± 0.48 1.55 ± 0.47 **1.89 × 10^-7^**

rs41417449 T/C (M295V) *TT* *TC* *CC*

1.46 ± 0.46 1.49 ± 0.48 1.55 ± 0.47 **1.90 × 10^-7^**

rs78587369 G/A (T165I) *GG* *GA* *AA*

1.46 ± 0.46 1.49 ± 0.48 1.55 ± 0.47 **1.95 × 10^-7^**

rs7412 C/T (R176C) *CC* *CT* *TT*

1.47 ± 0.47 1.55 ± 0.50 1.52 ± 0.49 **3.07 × 10^-7^**

rs34423804 T/A (V283D) *TT* *TA* *AA*

1.46 ± 0.46 1.49 ± 0.48 1.55 ± 0.47 **2.00 × 10^-7^**

rs75326924 C/T (P90S) *CC* *CT* *TT*

1.47 ± 0.47 1.54 ± 0.49 1.59 ± 0.43 **6.34 × 10^-7^**

rs7792133 G/A (R665H) *GG* *GA*

1.51 ± 0.46 1.21 ± 0.53 **2.03 × 10^-7^**

rs4314 C/T (R561W) *CC* *CT*

1.51 ± 0.46 1.20 ± 0.43 **2.42 × 10^-7^**

rs3806156 G/T *GG* *GT* *TT*

1.45 ± 0.46 1.48 ± 0.47 1.51 ± 0.47 **1.73 × 10^-6^**

rs150565858 G/A (R301Q) *GG* *GA*

1.49 ± 0.47 1.16 ± 0.41 **4.04 × 10^-7^**

rs3873352 G/C *GG* *GC* *CC*

1.46 ± 0.47 1.49 ± 0.47 1.52 ± 0.48 **2.22 × 10^-6^**

rs592229 G/T *GG* *GT* *TT*

1.51 ± 0.47 1.47 ± 0.47 1.45 ± 0.47 **2.56 × 10^-6^**

rs2523849 A/G *AA* *AG* *GG*

1.46 ± 0.47 1.49 ± 0.47 1.52 ± 0.48 **2.39 × 10^-6^**

rs2517518 G/A *GG* *GA* *AA*

1.46 ± 0.47 1.49 ± 0.47 1.52 ± 0.48 **2.39 × 10^-6^**

rs7914982 T/C (H244R) *TT* *TC*

1.50 ± 0.46 1.21 ± 0.35 **6.05 × 10^-7^**

rs9295895 T/C *TT* *TC* *CC*

1.47 ± 0.47 1.49 ± 0.46 1.53 ± 0.47 **3.41 × 10^-6^**

rs3895681 G/C *GG* *GC* *CC*

1.48 ± 0.47 1.40 ± 0.44 1.47 ± 0.55 **1.72 × 10^-6^**

rs139421991 G/A (R320Q) *GG* *GA*

1.48 ± 0.47 1.24 ± 0.46 **6.83 × 10^-7^**

rs138406927 C/T (A1096T) *CC* *CT*

1.50 ± 0.46 1.60 ± 0.42 **7.60 × 10^-7^**

rs2066714 C/T (M883I) *CC* *CT* *TT*

1.50 ± 0.48 1.47 ± 0.46 1.44 ± 0.46 **3.81 × 10^-6^**

Associated with hypo–HDL-cholesterolemia

rs147317864 C/T (A262T) *CC* *CT*

1.50 ± 0.46 1.16 ± 0.54 **3.16 × 10^-5^**

_________________________________________________________________________

Data were compared among genotypes by one-way ANOVA. Based on Bonferroni’s correction, *P* values of <4.76 × 10^–4^ (0.05/105) were considered statistically significant and are shown in bold.
