# Supplementary material for: Identification of eight genetic variants as novel determinants of dyslipidemia in Japanese by exome-wide association studies
Source: Oncotarget. 2017 Apr 17;8(24):38950–61. doi: 10.18632/oncotarget.17159 (PMC5503585; doi:10.18632/oncotarget.17159)
Supplement: Supplementary file 14 [file oncotarget-08-38950-s014.docx]

**Supplementary Table 15.** Relation of SNPs to the serum concentration of LDL-cholesterol.

_________________________________________________________________________

SNP Serum LDL-cholesterol (mmol/L) *P*

_________________________________________________________________________

Associated with serum LDL-cholesterol and hyper–LDL-cholesterolemia

rs2853969 C/T *CC* *CT* *TT*

3.10 ± 0.86 3.19 ± 0.88 3.30 ± 0.82 **1.32 × 10^-6^**

Associated with serum LDL-cholesterol

rs7412 C/T (R176C) *CC* *CT* *TT*

3.15 ± 0.86 2.74 ± 0.82 2.62 ± 1.27 **<1.0 × 10^-23^**

rs445925 C/T *CC* *CT* *TT*

3.14 ± 0.86 2.94 ± 0.86 3.01 ± 1.07 **4.33 × 10^-18^**

rs13306206 G/A (P955S) *GG* *GA* *AA*

3.10 ± 0.86 3.33 ± 0.95 4.12 ± 0.78 **1.64 × 10^-16^**

rs151193009 C/T (R93C) *CC* *CT* *TT*

3.13 ± 0.86 2.75 ± 0.74 2.36 ± 0.50 **3.24 × 10^-13^**

rs769449 G/A *GG* *GA* *AA*

3.10 ± 0.86 3.25 ± 0.85 3.17 ± 0.85 **1.53 × 10^-11^**

rs599839 A/G *AA* *AG* *GG*

3.14 ± 0.87 3.01 ± 0.81 2.88 ± 0.74 **1.23 × 10^-9^**

rs629301 A/C *AA* *AC* *CC*

3.14 ± 0.87 3.01 ± 0.81 2.86 ± 0.75 **1.57 × 10^-9^**

rs13306194 G/A (R532W) *GG* *GA* *AA*

3.14 ± 0.88 3.03 ± 0.81 2.97 ± 0.76 **1.90 × 10^-9^**

rs12740374 G/T *GG* *GT* *TT*

3.14 ± 0.87 3.01 ± 0.81 2.87 ± 0.76 **3.53 × 10^-9^**

rs602633 C/A *CC* *CA* *AA*

3.14 ± 0.87 3.02 ± 0.81 2.85 ± 0.77 **3.53 × 10^-9^**

rs646776 A/G *AA* *AG* *GG*

3.14 ± 0.87 3.01 ± 0.81 2.89 ± 0.75 **3.98 × 10^-9^**

rs1053878 G/A (P156L) *GG* *GA* *AA*

3.08 ± 0.85 3.17 ± 0.88 3.20 ± 0.87 **4.74 × 10^-9^**

rs651007 G/A *GG* *GA* *AA*

3.07 ± 0.85 3.17 ± 0.88 3.17 ± 0.87 **6.19 × 10^-9^**

rs579459 T/C *TT* *TC* *CC*

3.07 ± 0.85 3.17 ± 0.88 3.17 ± 0.87 **6.42 × 10^-9^**

rs635634 G/A *GG* *GA* *AA*

3.07 ± 0.85 3.16 ± 0.88 3.17 ± 0.87 **7.38 × 10^-9^**

rs507666 G/A *GG* *GA* *AA*

3.07 ± 0.85 3.16 ± 0.88 3.16 ± 0.87 **8.22 × 10^-9^**

rs117024916 A/G (T71A) *AA* *AG* *GG*

3.10 ± 0.86 3.19 ± 0.89 3.28 ± 0.84 **5.23 × 10^-7^**

rs11751198 G/A *GG* *GA* *AA*

3.10 ± 0.86 3.19 ± 0.88 3.31 ± 0.84 **1.03 × 10^-6^**

rs147733073 C/G (H486Q) *CC* *CG* *GG*

3.10 ± 0.84 3.18 ± 0.86 3.30 ± 0.84 **1.20 × 10^-6^**

rs11754464 C/T *CC* *CT* *TT*

3.10 ± 0.86 3.19 ± 0.88 3.31 ± 0.83 **1.74 × 10^-6^**

rs5030798 C/T (V1055I) *CC* *CT* *TT*

3.10 ± 0.86 3.19 ± 0.88 3.31 ± 0.84 **2.15 × 10^-6^**

rs11538264 G/A (V1774M) *GG* *GA* *AA*

3.10 ± 0.86 3.19 ± 0.88 3.31 ± 0.83 **2.22 × 10^-6^**

rs150142878 C/T (R371Q) *CC* *CT* *TT*

3.10 ± 0.86 3.22 ± 0.90 3.25 ±0.99 **1.83 × 10^-6^**

rs6457452 C/T *CC* *CT* *TT*

3.10 ± 0.86 3.18 ± 0.88 3.31 ± 0.82 **2.15 × 10^-6^**

rs117894946 G/C (G75A) *GG* *GC* *CC*

3.10 ± 0.86 3.19 ± 0.88 3.31 ± 0.83 **2.31 × 10^-6^**

rs11968400 C/T *CC* *CT* *TT*

3.10 ± 0.86 3.18 ± 0.88 3.31 ± 0.83 **2.40 × 10^-6^**

rs12210887 G/T *GG* *GT* *TT*

3.10 ± 0.86 3.18 ± 0.88 3.31 ± 0.83 **2.70 × 10^-6^**

rs4576240 G/T (P142T) *GG* *GT* *TT*

3.10 ± 0.86 3.23 ± 0.90 3.22 ± 1.00 **2.01 × 10^-6^**

rs2596574 G/A *GG* *GA* *AA*

3.10 ± 0.86 3.19 ± 0.88 3.29 ± 0.82 **3.51 × 10^-6^**

rs6922302 C/G (P128A) *CC* *CG* *GG*

3.10 ± 0.85 3.19 ± 0.90 3.26 ± 0.85 **4.08 × 10^-6^**

rs13118 T/A *TT* *TA* *AA*

3.10 ± 0.86 3.18 ± 0.88 3.30 ± 0.84 **3.70 × 10^-6^**

rs76463649 A/G (N15S) *AA* *AG* *GG*

3.10 ± 0.85 3.19 ± 0.90 3.28 ± 0.84 **4.34 × 10^-6^**

rs17200983 C/A (P34Q) *CC* *CA* *AA*

3.10 ± 0.86 3.18 ± 0.88 3.31 ± 0.83 **3.91 × 10^-6^**

rs3129029 A/C *AA* *AC* *CC*

3.09 ± 0.86 3.13 ± 0.86 3.25 ± 0.90 **9.91 × 10^-7^**

rs9267546 G/A *GG* *GA* *AA*

3.10 ± 0.86 3.18 ± 0.88 3.31 ± 0.82 **4.87 × 10^-6^**

rs9267547 G/A (A107T) *GG* *GA* *AA*

3.10 ± 0.86 3.18 ± 0.87 3.33 ± 0.84 **3.81 × 10^-6^**

rs140770834 C/G (L2271V) *CC* *CG* *GG*

3.10 ± 0.86 3.19 ± 0.88 3.31 ± 0.85 **6.40 × 10^-6^**

rs11751545 A/C *AA* *AC* *CC*

3.10 ± 0.86 3.19 ± 0.88 3.31 ± 0.85 **6.40 × 10^-6^**

rs4148249 C/A *CC* *CA* *AA*

3.10 ± 0.86 3.19 ± 0.89 3.25 ± 0.85 **7.20 × 10^-6^**

Associated with hyper–LDL-cholesterolemia

rs7771335 A/G *AA* *AG* *GG*

3.09 ± 0.86 3.14 ± 0.86 3.24 ± 0.90 **5.71 × 10^-6^**

rs76974938 C/T (D67N) *CC* *CT*

3.13 ± 0.86 3.04 ± 0.77 0.0134

rs2071653 C/T *CC* *CT* *TT*

3.09 ± 0.86 3.13 ± 0.86 3.22 ± 0.90 **2.26 × 10^-5^**

rs2269704 C/T *CC* *CT* *TT*

3.10 ± 0.85 3.17 ± 0.89 3.20 ± 0.85 **4.34 × 10^-5^**

rs2269703 G/A *GG* *GA* *AA*

3.10 ± 0.86 3.17 ± 0.89 3.20 ± 0.85 **4.65 × 10^-5^**

rs495089 T/C *TT* *TC* *CC*

3.09 ± 0.86 3.12 ± 0.86 3.17 ± 0.87 0.0018

rs2269702 A/G *AA* *AG* *GG*

3.10 ± 0.85 3.16 ± 0.89 3.21 ± 0.88 **1.03 × 10^-4^**

rs1233399 C/T *CC* *CT* *TT*

3.13 ± 0.88 3.10 ± 0.84 3.08 ± 0.85 0.1451

_________________________________________________________________________

Data were compared among genotypes by one-way ANOVA. Based on Bonferroni’s correction, *P* values of <0.0010 (0.05/48) were considered statistically significant and are shown in bold.
