# Supplementary material for: Identification of eight genetic variants as novel determinants of dyslipidemia in Japanese by exome-wide association studies
Source: Oncotarget. 2017 Apr 17;8(24):38950–61. doi: 10.18632/oncotarget.17159 (PMC5503585; doi:10.18632/oncotarget.17159)
Supplement: Supplementary file 15 [file oncotarget-08-38950-s015.docx]

**Supplementary Table 16.** Relation of genes, loci, and SNPs identified in the present analysis of serum triglycerides to phenotypes previously examined in GWASs.

| Gene  (or chr. locus) | SNP | Nucleotide  (amino acid)  substitution | Previously examined phenotypes |
| --- | --- | --- | --- |
| Associated with serum triglycerides and hypertriglyceridemia | | | |
| *BUD13* | rs10790162 | G/A | [**Triglyceride levels**](http://www.ebi.ac.uk/gwas/search?query=Triglyceride%20levels) (PMID: 26763881), [HDL-cholesterol](http://www.ebi.ac.uk/gwas/search?query=HDL%20cholesterol) (PMID: 26780889), [postbronchodilator FEV1/FVC ratio](http://www.ebi.ac.uk/gwas/search?query=Post%20bronchodilator%20FEV1/FVC%20ratio) (PMID: 26634245), [severe influenza A (H1N1) infection](http://www.ebi.ac.uk/gwas/search?query=Severe%20influenza%20A%20(H1N1)%20infection) (PMID: 26379185), [manganese levels](http://www.ebi.ac.uk/gwas/search?query=Manganese%20levels) (PMID: 26025379), [HDL-cholesterol–**triglycerides**](http://www.ebi.ac.uk/gwas/search?query=HDL%20Cholesterol%20-%20Triglycerides%20(HDLC-TG)), [waist circumference–**triglycerides**](http://www.ebi.ac.uk/gwas/search?query=Waist%20Circumference%20-%20Triglycerides%20(WC-TG)), and [metabolic syndrome](http://www.ebi.ac.uk/gwas/search?query=Metabolic%20syndrome) (PMID: 21386085) |
| 11q23.3 | rs7350481 | C/T | [**Triglycerides**](http://www.ebi.ac.uk/gwas/search?query=Triglycerides), [HDL-cholesterol](http://www.ebi.ac.uk/gwas/search?query=HDL%20cholesterol), and total [cholesterol](http://www.ebi.ac.uk/gwas/search?query=Cholesterol,%20total) (PMID: 26780889), [**triglycerides**](http://www.ebi.ac.uk/gwas/search?query=Triglycerides) and [hematologic and biochemical traits](http://www.ebi.ac.uk/gwas/search?query=Hematological%20and%20biochemical%20traits) (PMID: 20139978), [parental extreme longevity](http://www.ebi.ac.uk/gwas/search?query=Parental%20extreme%20longevity%20(95%20years%20and%20older)) (PMID: 27015805), [aggressiveness in attention deficit–hyperactivity disorder](http://www.ebi.ac.uk/gwas/search?query=Aggressiveness%20in%20attention%20deficit%20hyperactivity%20disorder) (PMID: 27021288) |
| Associated with serum triglycerides | | | |
| *APOA5* | rs2075291  rs2266788 | C/A (G185C)  T/C | [**Triglycerides**](http://www.ebi.ac.uk/gwas/search?query=Triglycerides), [HDL-cholesterol](http://www.ebi.ac.uk/gwas/search?query=HDL%20cholesterol), and total [cholesterol](http://www.ebi.ac.uk/gwas/search?query=Cholesterol,%20total) (PMID: 26780889), [**triglyceride levels**](http://www.ebi.ac.uk/gwas/search?query=Triglyceride%20levels) (PMID: 26763881), [plasma omega-6 polyunsaturated fatty acid levels](http://www.ebi.ac.uk/gwas/search?query=Plasma%20omega-6%20polyunsaturated%20fatty%20acid%20levels%20(dihomo-gamma-linolenic%20acid)) (PMID: 26584805), [**triglycerides**–blood pressure](http://www.ebi.ac.uk/gwas/search?query=Triglycerides-Blood%20Pressure%20(TG-BP)), [HDL-cholesterol–**triglycerides**](http://www.ebi.ac.uk/gwas/search?query=HDL%20Cholesterol%20-%20Triglycerides%20(HDLC-TG)), [waist circumference–**triglycerides**](http://www.ebi.ac.uk/gwas/search?query=Waist%20Circumference%20-%20Triglycerides%20(WC-TG)), and [metabolic syndrome](http://www.ebi.ac.uk/gwas/search?query=Metabolic%20syndrome) (PMID: 21386085) |
| *ZPR1* | rs964184  rs2075290 | C/G  T/C | [**Triglycerides**](http://www.ebi.ac.uk/gwas/search?query=Triglycerides) and total [cholesterol](http://www.ebi.ac.uk/gwas/search?query=Cholesterol,%20total) (PMID: 26780889), [**postprandial triglyceride response to high-fat diet meal**](http://www.ebi.ac.uk/gwas/search?query=Postprandial%20triglyceride%20response%20to%20high%20fat%20diet%20meal) (PMID: 26256467), [total cholesterol](http://www.ebi.ac.uk/gwas/search?query=Cholesterol,%20total) and [LDL-cholesterol](http://www.ebi.ac.uk/gwas/search?query=LDL%20cholesterol) (PMID: 25961943), [HDL-cholesterol–**triglycerides**](http://www.ebi.ac.uk/gwas/search?query=HDL%20Cholesterol%20-%20Triglycerides%20(HDLC-TG)), [waist circumference–**triglycerides**](http://www.ebi.ac.uk/gwas/search?query=Waist%20Circumference%20-%20Triglycerides%20(WC-TG)), and [metabolic syndrome](http://www.ebi.ac.uk/gwas/search?query=Metabolic%20syndrome) (PMID: 21386085) |
| 11q23.3 | rs9326246  rs12269901  rs4938303 | G/C  G/C  T/C | Total [cholesterol](http://www.ebi.ac.uk/gwas/search?query=Cholesterol,%20total), [HDL-cholesterol](http://www.ebi.ac.uk/gwas/search?query=HDL%20cholesterol), and [**triglycerides**](http://www.ebi.ac.uk/gwas/search?query=Triglycerides) (PMID: 26780889), [HDL-cholesterol](http://www.ebi.ac.uk/gwas/search?query=HDL%20cholesterol) (PMID: 21347282), [**triglycerides**](http://www.ebi.ac.uk/gwas/search?query=Triglycerides) (PMID: 20864672), [aggressiveness in attention deficit–hyperactivity disorder](http://www.ebi.ac.uk/gwas/search?query=Aggressiveness%20in%20attention%20deficit%20hyperactivity%20disorder) (PMID: 27021288), [parental extreme longevity](http://www.ebi.ac.uk/gwas/search?query=Parental%20extreme%20longevity%20(95%20years%20and%20older)) (PMID: 27015805) |
| *MTFR2* | rs143974258 | G/A (R360*) | [Bipolar disorder](http://www.ebi.ac.uk/gwas/search?query=Bipolar%20disorder%20(body%20mass%20index%20interaction)) (PMID: 24322204) |
| *APOA4* | rs5104 | T/C (N147S) | [**Triglyceride levels**](http://www.ebi.ac.uk/gwas/search?query=Triglyceride%20levels) (PMID: 26763881), [**triglycerides**](http://www.ebi.ac.uk/gwas/search?query=Triglycerides) and [HDL-cholesterol](http://www.ebi.ac.uk/gwas/search?query=HDL%20cholesterol) (PMID: 26582766), [lipid traits](http://www.ebi.ac.uk/gwas/search?query=Lipid%20traits) (PMID: 24386095, PMID: 24023260) |
| *C21orf59* | rs76974938 | C/T (D67N) | None |
| *LPL* | rs328  rs15285  rs13702  rs326  rs301 | C/G (S474*)  G/A  A/G  A/G  T/C | [**Triglyceride levels**](http://www.ebi.ac.uk/gwas/search?query=Triglyceride%20levels) (PMID: 26763881, PMID: 26582766, PMID: 22171074, PMID: 18193044, PMID: 23726366, PMID: 18193046), [lipid traits](http://www.ebi.ac.uk/gwas/search?query=Lipid%20traits) (PMID: 24386095), [HDL-cholesterol](http://www.ebi.ac.uk/gwas/search?query=HDL%20cholesterol) (PMID: 26780889, PMID: 26763881, PMID: 26582766, PMID: 18193044, PMID: 23726366), [**triglycerides**–blood pressure](http://www.ebi.ac.uk/gwas/search?query=Triglycerides-Blood%20Pressure%20(TG-BP)), [HDL-cholesterol–**triglycerides**](http://www.ebi.ac.uk/gwas/search?query=HDL%20Cholesterol%20-%20Triglycerides%20(HDLC-TG)), and [metabolic syndrome](http://www.ebi.ac.uk/gwas/search?query=Metabolic%20syndrome%20(bivariate%20traits)) (PMID: 21386085) |
| 8p21.3 | rs10096633  rs17482753  rs12678919  rs10503669  rs7016880  rs2197089  rs1441756  rs2083637 | C/T  G/T  A/G  C/A  G/C  C/T  T/G  T/C | [**Triglyceride levels**](http://www.ebi.ac.uk/gwas/search?query=Triglyceride%20levels) (PMID: 26763881, PMID: 20139978, PMID: 19060911, PMID: 26582766, PMID: 24097068, PMID: 21909109, PMID: 18193043), [**hypertriglyceridemia**](http://www.ebi.ac.uk/gwas/search?query=Hypertriglyceridemia) (PMID: 20657596), [HDL-cholesterol](http://www.ebi.ac.uk/gwas/search?query=HDL%20cholesterol) (PMID: 26780889, PMID: 26763881, PMID: 21347282, PMID: 20031538, PMID: 26582766, PMID: 24097068, PMID: 21909109, PMID: 18193043, PMID: 19060911), [metabolic traits](http://www.ebi.ac.uk/gwas/search?query=Metabolic%20traits) (PMID: 19060910), [lipid traits](http://www.ebi.ac.uk/gwas/search?query=Lipid%20traits) (PMID: 24023261), [metabolic syndrome](http://www.ebi.ac.uk/gwas/search?query=Metabolic%20syndrome%20(bivariate%20traits)) (PMID: 21386085, PMID: 20694148), [waist circumference and related phenotypes](http://www.ebi.ac.uk/gwas/search?query=Waist%20circumference%20and%20related%20phenotypes) (PMID: 18454146), [erythrocyte cadmium concentration in never-smokers](http://www.ebi.ac.uk/gwas/search?query=Erythrocyte%20cadmium%20concentration%20in%20never%20smokers) (PMID: 27005419), [response to lithium treatment in bipolar disorder](http://www.ebi.ac.uk/gwas/search?query=Response%20to%20lithium%20treatment%20in%20bipolar%20disorder) (PMID: 26806518) |
| *SIK3* | rs2075292  rs10047462 | G/T  G/T | [**Triglycerides**](http://www.ebi.ac.uk/gwas/search?query=Triglycerides) (PMID: 24886709, PMID: 18193046), [HDL-cholesterol](http://www.ebi.ac.uk/gwas/search?query=HDL%20cholesterol) (PMID: 24886709), [visceral adipose tissue adjusted for BMI](http://www.ebi.ac.uk/gwas/search?query=Visceral%20adipose%20tissue%20adjusted%20for%20BMI) (PMID: 22589738), [response to vitamin E supplementation](http://www.ebi.ac.uk/gwas/search?query=Response%20to%20Vitamin%20E%20supplementation) (PMID: 22437554), [iron status biomarkers](http://www.ebi.ac.uk/gwas/search?query=Iron%20status%20biomarkers) (PMID: 21149283), [hearing function](http://www.ebi.ac.uk/gwas/search?query=Hearing%20function) (PMID: 25060954) |
| *GCKR* | rs1260326  rs780093 | T/C (L446P)  A/G | [**Triglycerides**](http://www.ebi.ac.uk/gwas/search?query=Triglycerides) (PMID: 26780889), [circulating leptin levels](http://www.ebi.ac.uk/gwas/search?query=circulating%20leptin%20levels%20adjusted%20for%20BMI) (PMID: 26833098), [type 2 diabetes](http://www.ebi.ac.uk/gwas/search?query=Type%202%20diabetes) (PMID: 26818947), [glomerular filtration rate](http://www.ebi.ac.uk/gwas/search?query=Glomerular%20filtration%20rate%20(creatinine)) (PMID: 26831199), [plasma lactate levels](http://www.ebi.ac.uk/gwas/search?query=Plasma%20lactate%20levels) (PMID: 26433129), [inflammatory bowel disease](http://www.ebi.ac.uk/gwas/search?query=Inflammatory%20bowel%20disease) (PMID: 26192919), [serum alpha1-antitrypsin levels](http://www.ebi.ac.uk/gwas/search?query=Serum%20alpha1-antitrypsin%20levels) (PMID: 26174136), [glucose homeostasis traits](http://www.ebi.ac.uk/gwas/search?query=Glucose%20homeostasis%20traits) (PMID: 25524916) |
| 2p23 | rs1260333 | T/C | [**Triglycerides**](http://www.ebi.ac.uk/gwas/search?query=Triglycerides) (PMID: 26582766, PMID: 20864672), total [cholesterol](http://www.ebi.ac.uk/gwas/search?query=Cholesterol,%20total) (PMID: 26582766) |
| *TNC* | rs138406927 | C/T (A1096T) | [Developmental language disorder](http://www.ebi.ac.uk/gwas/search?query=Developmental%20language%20disorder%20(syntactic%20complexity)) (PMID: 27016271), [postbronchodilator FEV1 in chronic obstructive pulmonary disease](http://www.ebi.ac.uk/gwas/search?query=Post%20bronchodilator%20FEV1%20in%20COPD) (PMID: 26634245), [plasma omega-3 polyunsaturated fatty acid level](http://www.ebi.ac.uk/gwas/search?query=Plasma%20omega-3%20polyunsaturated%20fatty%20acid%20level%20(eicosapentaenoic%20acid)) (PMID: 26584805), [cerebral amyloid deposition in APOEε4 noncarriers](http://www.ebi.ac.uk/gwas/search?query=Cerebral%20amyloid%20deposition%20in%20APOEe4%20non-carriers%20(PET%20imaging)) (PMID: 26252872), [glucose homeostasis traits](http://www.ebi.ac.uk/gwas/search?query=Glucose%20homeostasis%20traits) (PMID: 25524916) |
| *LAIR2* | rs34429135 | T/A (F115Y) | None |
| *PAFAH1B2* | rs7112513  rs4936367 | G/A  A/G (V151M) | [Aspirin hydrolysis](http://www.ebi.ac.uk/gwas/search?query=Aspirin%20hydrolysis%20(plasma)) (PMID: 23508960), [protein quantitative trait loci](http://www.ebi.ac.uk/gwas/search?query=Protein%20quantitative%20trait%20loci) (PMID: 18464913) |
| *C2orf16* | rs1919128  rs1919127 | G/A (V774I)  C/T (A685V) | [**Hypertriglyceridemia**](http://www.ebi.ac.uk/gwas/search?query=Hypertriglyceridemia) (PMID: 23505323), [palmitoleic acid plasma levels](http://www.ebi.ac.uk/gwas/search?query=Palmitoleic%20acid%20(16:1n-7)%20plasma%20levels) (PMID: 23362303), [liver enzyme (gamma–glutamyl transferase)](http://www.ebi.ac.uk/gwas/search?query=Liver%20enzyme%20levels%20(gamma-glutamyl%20transferase)) levels (PMID: 22001757), [waist circumference–**triglycerides**](http://www.ebi.ac.uk/gwas/search?query=Waist%20Circumference%20-%20Triglycerides%20(WC-TG)) (PMID: 21386085), [postbronchodilator FEV1/FVC ratio](http://www.ebi.ac.uk/gwas/search?query=Post%20bronchodilator%20FEV1/FVC%20ratio) (PMID: 26634245) |
| *OR4F6* | rs141569282 | G/A (A117T) | None |
| 8q24.1 | rs2954038  rs2954033  rs2954026 | A/C  G/A  G/T | [**Triglycerides**–blood pressure](http://www.ebi.ac.uk/gwas/search?query=Triglycerides-Blood%20Pressure%20(TG-BP)) (PMID: 21386085) [HDL-cholesterol–**triglycerides**](http://www.ebi.ac.uk/gwas/search?query=HDL%20Cholesterol%20-%20Triglycerides%20(HDLC-TG)) (PMID: 21386085), [response to statin therapy](http://www.ebi.ac.uk/gwas/search?query=Response%20to%20statin%20therapy) (PMID: 20339536) |
| LOC101929011 | rs1240773 | G/T | [**Triglycerides**](http://www.ebi.ac.uk/gwas/search?query=Triglycerides) (PMID: 20864672), [LDL-cholesterol](http://www.ebi.ac.uk/gwas/search?query=LDL%20cholesterol) (PMID: 20864672), [HDL-cholesterol](http://www.ebi.ac.uk/gwas/search?query=HDL%20cholesterol) (PMID: 26780889, PMID: 21909109), [HIV-1 susceptibility](http://www.ebi.ac.uk/gwas/search?query=HIV-1%20susceptibility) (PMID: 21160409) |
| *COL6A5* | rs200982668 | G/A (E2501K) | [BMI](http://www.ebi.ac.uk/gwas/search?query=Body%20mass%20index) (PMID: 24348519) |
| 6p21.3 | rs1264429 | A/G | [Age-related macular degeneration](http://www.ebi.ac.uk/gwas/search?query=Age-related%20macular%20degeneration) (PMID: 22694956), [nevirapine-induced rash](http://www.ebi.ac.uk/gwas/search?query=Nevirapine-induced%20rash) (PMID: 21810746), [chronic lymphocytic leukemia](http://www.ebi.ac.uk/gwas/search?query=Chronic%20lymphocytic%20leukemia) (PMID: 21131588), [nasopharyngeal carcinoma](http://www.ebi.ac.uk/gwas/search?query=Nasopharyngeal%20carcinoma) (PMID: 19664746) |
| *MUC17* | rs78010183 | A/T (T1305S) | None |
| *MARCH1* | rs61734696 | G/T (Q137K) | [Type 2 diabetes](http://www.ebi.ac.uk/gwas/search?query=Type%202%20diabetes) (PMID: 21490949), [postbronchodilator FEV1/FVC ratio](http://www.ebi.ac.uk/gwas/search?query=Post%20bronchodilator%20FEV1/FVC%20ratio) (PMID: 26634245), [diisocyanate-induced asthma](http://www.ebi.ac.uk/gwas/search?query=Diisocyanate-induced%20asthma) (PMID: 25918132), [urinary uromodulin levels](http://www.ebi.ac.uk/gwas/search?query=Urinary%20uromodulin%20levels) (PMID: 24578125) |
| *MRVI1* | rs4909945 | C/T (V11I) | [Migraine](http://www.ebi.ac.uk/gwas/search?query=Migraine) (PMID: 23793025), [bipolar disorder and schizophrenia](http://www.ebi.ac.uk/gwas/search?query=Bipolar%20disorder%20and%20schizophrenia) (PMID: 20889312), [platelet aggregation](http://www.ebi.ac.uk/gwas/search?query=Platelet%20aggregation) (PMID: 20526338) |

Data were obtained from GWAS Catalog (http://www.ebi.ac.uk/gwas), and phenotypes related to serum triglycerides are shown in bold. PMID, PubMed ID; chr., chromosome.
