# Supplementary material for: Identification of eight genetic variants as novel determinants of dyslipidemia in Japanese by exome-wide association studies
Source: Oncotarget. 2017 Apr 17;8(24):38950–61. doi: 10.18632/oncotarget.17159 (PMC5503585; doi:10.18632/oncotarget.17159)
Supplement: Supplementary file 16 [file oncotarget-08-38950-s016.docx]

**Supplementary Table 17.** Relation of genes, loci, and SNPs identified in the present analysis of serum HDL–cholesterol to phenotypes examined in previous GWASs.

| Gene  (or chr. locus) | SNP | Nucleotide  (amino acid)  substitution | Previously examined phenotypes |
| --- | --- | --- | --- |
| Associated with serum HDL-cholesterol and hypo–HDL-cholesterolemia | | | |
| *USP4* | rs146515657 | T/C (N650S) | [Educational attainment](http://www.ebi.ac.uk/gwas/search?query=Educational%20attainment) (PMID: 27046643), [ulcerative colitis](http://www.ebi.ac.uk/gwas/search?query=Ulcerative%20colitis) (PMID: 25082827), [inflammatory bowel disease](http://www.ebi.ac.uk/gwas/search?query=Inflammatory%20bowel%20disease) (PMID: 23128233) |
| 12q24.1 | rs12229654 | T/G | [**HDL-cholesterol**](http://www.ebi.ac.uk/gwas/search?query=HDL%20cholesterol) (PMID: 21909109), [metabolic syndrome](http://www.ebi.ac.uk/gwas/search?query=Metabolic%20syndrome) (PMID: 25705158), BMI (PMID: 24861553), [glycemic traits](http://www.ebi.ac.uk/gwas/search?query=Glycemic%20traits) (PMID: 23575436), [gamma–glutamyl transpeptidase](http://www.ebi.ac.uk/gwas/search?query=Gamma%20glutamyl%20transpeptidase) (PMID: 21909109) |
| Associated with serum HDL-cholesterol | | | |
| *LPGAT1* | rs150552771 | T/C (K200E) | [Stearic acid (18:0) plasma levels](http://www.ebi.ac.uk/gwas/search?query=Stearic%20acid%20(18:0)%20plasma%20levels) (PMID: 23362303) |
| *LAIR2* | rs34429135 | T/A (F115Y) | None |
| *KRR1* | rs17115182 | G/A (P43S) | [Polycystic ovary syndrome](http://www.ebi.ac.uk/gwas/search?query=Polycystic%20ovary%20syndrome) (PMID: 26416764), [blood Cu levels](http://www.ebi.ac.uk/gwas/search?query=Blood%20trace%20element%20(Cu%20levels)) (PMID: 23720494) |
| *EHD3* | rs116417209 | G/A (V151I) | [Mean platelet volume](http://www.ebi.ac.uk/gwas/search?query=Mean%20platelet%20volume) (PMID: 24026423, PMID: 22139419, PMID: 19820697), [estradiol levels](http://www.ebi.ac.uk/gwas/search?query=Estradiol%20levels) (PMID: 22675492), [platelet count](http://www.ebi.ac.uk/gwas/search?query=Platelet%20count) (PMID: 22139419) |
| 16q13 | rs3764261  rs247616  rs173539 | G/T  C/T  C/T | [**HDL-cholesterol**](http://www.ebi.ac.uk/gwas/search?query=HDL%20cholesterol) (PMID: 26780889, PMID: 26763881, PMID: 26582766), [**low HDL-cholesterol levels**](http://www.ebi.ac.uk/gwas/search?query=Low%20high%20density%20lipoprotein%20cholesterol%20levels) (PMID: 26879886), [**HDL-cholesterol in HIV infection**](http://www.ebi.ac.uk/gwas/search?query=HDL%20Cholesterol%20in%20HIV-infection) (PMID: 25884002), [total cholesterol](http://www.ebi.ac.uk/gwas/search?query=Cholesterol,%20total) (PMID: 26582766), [**HDL-cholesterol**–triglycerides](http://www.ebi.ac.uk/gwas/search?query=HDL%20Cholesterol%20-%20Triglycerides%20(HDLC-TG)) (PMID: 21386085), [morning vs. evening chronotype](http://www.ebi.ac.uk/gwas/search?query=Morning%20vs.%20evening%20chronotype) (PMID: 26835600), [advanced age-related macular degeneration](http://www.ebi.ac.uk/gwas/search?query=Advanced%20age-related%20macular%20degeneration) (PMID: 26691988), [exudative age-related macular degeneration](http://www.ebi.ac.uk/gwas/search?query=Exudative%20age-related%20macular%20degeneration) (PMID: 25629512), [blood metabolite levels](http://www.ebi.ac.uk/gwas/search?query=Blood%20metabolite%20levels) (PMID: 24816252, PMID: 22916037), [lipoprotein-associated phospholipase A_2_ activity and mass](http://www.ebi.ac.uk/gwas/search?query=Lipoprotein-associated%20phospholipase%20A2%20activity%20and%20mass) (PMID: 22003152), [cardiovascular disease risk factors](http://www.ebi.ac.uk/gwas/search?query=Cardiovascular%20disease%20risk%20factors) (PMID: 20838585, PMID: 21943158), [metabolic syndrome](http://www.ebi.ac.uk/gwas/search?query=Metabolic%20syndrome%20(bivariate%20traits)) (PMID: 21386085) |
| 6p22.1 | rs9261800  rs9295895 | C/G  T/C | [Parental extreme longevity](http://www.ebi.ac.uk/gwas/search?query=Parental%20extreme%20longevity%20(95%20years%20and%20older)) (PMID: 27015805), [chronic lymphocytic leukemia](http://www.ebi.ac.uk/gwas/search?query=Chronic%20lymphocytic%20leukemia) (PMID: 26956414), [glomerular filtration rate](http://www.ebi.ac.uk/gwas/search?query=Glomerular%20filtration%20rate%20(creatinine)) (PMID: 26831199), [platelet count](http://www.ebi.ac.uk/gwas/search?query=Platelet%20count) (PMID: 26805783), [late-onset myasthenia gravis](http://www.ebi.ac.uk/gwas/search?query=Late-onset%20myasthenia%20gravis) (PMID: 26562150) |
| *DCLRE1C* | rs150854849 | C/T (R179Q) | [Migraine](http://www.ebi.ac.uk/gwas/search?query=Migraine) (PMID: 23793025) |
| *STYK1* | rs138533962 | G/A (R379C) | None |
| *CETP* | rs2303790  rs1532624  rs9939224  rs7499892  rs1800775 | A/G (D459G)  G/T  G/T  C/T  A/C | [**HDL-cholesterol**](http://www.ebi.ac.uk/gwas/search?query=HDL%20cholesterol) (PMID: 26780889, PMID: 26763881, PMID: 26582766, PMID: 23505323, PMID: 19060911, PMID: 20031564), [**low HDL-cholesterol levels**](http://www.ebi.ac.uk/gwas/search?query=Low%20high%20density%20lipoprotein%20cholesterol%20levels) (PMID: 26879886), [age-related macular degeneration](http://www.ebi.ac.uk/gwas/search?query=Advanced%20age-related%20macular%20degeneration) (PMID: 26691988, PMID: 25629512), [cholesterol](http://www.ebi.ac.uk/gwas/search?query=Cholesterol) (PMID: 20066028), [lipid metabolism phenotypes](http://www.ebi.ac.uk/gwas/search?query=Lipid%20metabolism%20phenotypes) (PMID: 19936222), [metabolic syndrome](http://www.ebi.ac.uk/gwas/search?query=Metabolic%20syndrome%20(bivariate%20traits)) (PMID: 21386085), [biochemical measures](http://www.ebi.ac.uk/gwas/search?query=Biochemical%20measures) (PMID: 19260141), [blood metabolite levels](http://www.ebi.ac.uk/gwas/search?query=Blood%20metabolite%20levels) (PMID: 24816252), [lipid traits](http://www.ebi.ac.uk/gwas/search?query=Lipid%20traits) (PMID: 24023260) |
| *APOA5* | rs2075291  rs2266788 | C/A (G185C)  T/C | [**HDL-cholesterol**](http://www.ebi.ac.uk/gwas/search?query=HDL%20cholesterol) (PMID: 26780889), [total cholesterol](http://www.ebi.ac.uk/gwas/search?query=Cholesterol,%20total) (PMID: 26780889), [triglycerides](http://www.ebi.ac.uk/gwas/search?query=Triglycerides) (PMID: 26780889, PMID: 26763881), [**HDL-cholesterol**–triglycerides](http://www.ebi.ac.uk/gwas/search?query=HDL%20Cholesterol%20-%20Triglycerides%20(HDLC-TG)), [triglycerides–blood pressure](http://www.ebi.ac.uk/gwas/search?query=Triglycerides-Blood%20Pressure%20(TG-BP)), [waist circumference–triglycerides](http://www.ebi.ac.uk/gwas/search?query=Waist%20Circumference%20-%20Triglycerides%20(WC-TG)), and [metabolic syndrome](http://www.ebi.ac.uk/gwas/search?query=Metabolic%20syndrome) (PMID: 21386085), [plasma omega-6 polyunsaturated fatty acid levels](http://www.ebi.ac.uk/gwas/search?query=Plasma%20omega-6%20polyunsaturated%20fatty%20acid%20levels%20(dihomo-gamma-linolenic%20acid)) (PMID: 26584805) |
| *MUC17* | rs78010183 | A/T (T1305S) | None |
| *LIPC* | rs1800588  rs261334 | T/C  G/C | [**HDL-cholesterol**](http://www.ebi.ac.uk/gwas/search?query=HDL%20cholesterol) (PMID: 26780889, PMID: 26582766, PMID: 18193044, PMID: 26780889, PMID: 20864672), [advanced age-related macular degeneration](http://www.ebi.ac.uk/gwas/search?query=Advanced%20age-related%20macular%20degeneration) (PMID: 26691988), [triglycerides](http://www.ebi.ac.uk/gwas/search?query=Triglycerides) (PMID: 26582766), [total cholesterol](http://www.ebi.ac.uk/gwas/search?query=Cholesterol,%20total) (PMID: 26582766) |
| *OR4F6* | rs141569282 | G/A (A117T) | None |
| *CYP4F8* | rs201166643 | C/A (R488S) | [Inflammatory biomarkers](http://www.ebi.ac.uk/gwas/search?query=Inflammatory%20biomarkers) (PMID: 22228203) |
| *ACAD10* | rs11066015 | G/A | [Advanced age-related macular degeneration](http://www.ebi.ac.uk/gwas/search?query=Advanced%20age-related%20macular%20degeneration) (PMID: 26691988), [colorectal cancer](http://www.ebi.ac.uk/gwas/search?query=Colorectal%20cancer) (PMID: 26151821), [coronary heart disease](http://www.ebi.ac.uk/gwas/search?query=Coronary%20heart%20disease) (PMID: 23364394), [mean platelet volume](http://www.ebi.ac.uk/gwas/search?query=Mean%20platelet%20volume) (PMID: 22423221), [esophageal cancer](http://www.ebi.ac.uk/gwas/search?query=Esophageal%20cancer) (PMID: 21642993) |
| *ALDH2* | rs671 | G/A (E504K) | [Ischemic stroke](http://www.ebi.ac.uk/gwas/search?query=Ischemic%20stroke) (PMID: 26708676), [triglycerides](http://www.ebi.ac.uk/gwas/search?query=Triglycerides) (PMID: 26582766), [primary biliary cirrhosis](http://www.ebi.ac.uk/gwas/search?query=Primary%20biliary%20cirrhosis) (PMID: 26394269), [systolic blood pressure](http://www.ebi.ac.uk/gwas/search?query=Systolic%20blood%20pressure) (PMID: 26390057), [serum alpha1-antitrypsin levels](http://www.ebi.ac.uk/gwas/search?query=Serum%20alpha1-antitrypsin%20levels) (PMID: 26174136), BMI (PMID: 24861553), [alcohol dependence](http://www.ebi.ac.uk/gwas/search?query=Alcohol%20dependence), [consumption, and](http://www.ebi.ac.uk/gwas/search?query=Alcohol%20consumption%20(maxi-drinks)) flushing response (PMID: 24277619) |
| *CACNA1D* | rs35874056 | G/A (G460S) | [Visceral adipose tissue/subcutaneous adipose tissue ratio](http://www.ebi.ac.uk/gwas/search?query=Visceral%20adipose%20tissue/subcutaneous%20adipose%20tissue%20ratio) (PMID: 26480920), [BMI](http://www.ebi.ac.uk/gwas/search?query=Body%20mass%20index) (PMID: 25673413), [trans–fatty acid levels](http://www.ebi.ac.uk/gwas/search?query=Trans%20fatty%20acid%20levels) (PMID: 25646338), [systolic blood pressure](http://www.ebi.ac.uk/gwas/search?query=Systolic%20blood%20pressure) (PMID: 25249183), [hypertension](http://www.ebi.ac.uk/gwas/search?query=Hypertension) (PMID: 25249183) |
| *BRAP* | rs3782886 | A/G | [Total cholesterol](http://www.ebi.ac.uk/gwas/search?query=Cholesterol,%20total) (PMID: 24097068), [primary biliary cirrhosis](http://www.ebi.ac.uk/gwas/search?query=Primary%20biliary%20cirrhosis) (PMID: 26394269), [colorectal cancer](http://www.ebi.ac.uk/gwas/search?query=Colorectal%20cancer) (PMID: 26151821), BMI (PMID: 25673413), [myocardial infarction](http://www.ebi.ac.uk/gwas/search?query=Myocardial%20infarction) (PMID: 24916648), [hematologic and biochemical traits](http://www.ebi.ac.uk/gwas/search?query=Hematological%20and%20biochemical%20traits) (PMID: 20139978) |
| *HECTD4* | rs2074356  rs11066280 | C/T  T/A | [**HDL-cholesterol**](http://www.ebi.ac.uk/gwas/search?query=HDL%20cholesterol) (PMID: 21909109), [thoracic-to-hip circumference ratio](http://www.ebi.ac.uk/gwas/search?query=Thoracic-to-hip%20circumference%20ratio) (PMID: 26675016), [triglycerides](http://www.ebi.ac.uk/gwas/search?query=Triglycerides) (PMID: 26582766), [systolic and diastolic blood pressure](http://www.ebi.ac.uk/gwas/search?query=Systolic%20blood%20pressure) (PMID: 26390057), BMI (PMID: 25673413), [glycemic traits](http://www.ebi.ac.uk/gwas/search?query=Glycemic%20traits) (PMID: 23575436), [renal function–related traits](http://www.ebi.ac.uk/gwas/search?query=Renal%20function-related%20traits%20(BUN)) (PMID: 22797727), [gamma–glutamyl transpeptidase](http://www.ebi.ac.uk/gwas/search?query=Gamma%20glutamyl%20transpeptidase) (PMID: 21909109), [esophageal cancer](http://www.ebi.ac.uk/gwas/search?query=Esophageal%20cancer) (PMID: 21642993), [thoracic-to-hip circumference ratio](http://www.ebi.ac.uk/gwas/search?query=Thoracic-to-hip%20circumference%20ratio) (PMID: 26675016), [metabolic syndrome](http://www.ebi.ac.uk/gwas/search?query=Metabolic%20syndrome) (PMID: 25705158) |
| *LILRB2* | rs73055442 | C/T (R103H) | [**HDL-cholesterol**](http://www.ebi.ac.uk/gwas/search?query=HDL%20cholesterol) (PMID: 20686565), [postbronchodilator FEV1/FVC ratio](http://www.ebi.ac.uk/gwas/search?query=Post%20bronchodilator%20FEV1/FVC%20ratio) (PMID: 26634245) |
| *COL6A5* | rs200982668 | G/A (E2501K) | BMI (PMID: 24348519) |
| *VPS33B* | rs199921354 | C/T (R80Q) | [Type 2 diabetes](http://www.ebi.ac.uk/gwas/search?query=Type%202%20diabetes) (PMID: 24509480) |
| *MARCH1* | rs61734696 | G/T (Q137K) | [Type 2 diabetes](http://www.ebi.ac.uk/gwas/search?query=Type%202%20diabetes) (PMID: 21490949), [urinary uromodulin levels](http://www.ebi.ac.uk/gwas/search?query=Urinary%20uromodulin%20levels) (PMID: 24578125), [diisocyanate-induced asthma](http://www.ebi.ac.uk/gwas/search?query=Diisocyanate-induced%20asthma) (PMID: 25918132), [postbronchodilator FEV1/FVC ratio](http://www.ebi.ac.uk/gwas/search?query=Post%20bronchodilator%20FEV1/FVC%20ratio) (PMID: 26634245) |
| *SLC9A3* | rs143027124 | C/T (V213I) | [Lung disease severity in cystic fibrosis](http://www.ebi.ac.uk/gwas/search?query=Lung%20disease%20severity%20in%20cystic%20fibrosis) (PMID: 26417704), [ulcerative colitis](http://www.ebi.ac.uk/gwas/search?query=Ulcerative%20colitis) (PMID: 23128233) |
| *MOB3C* | rs139537100 | C/T (R24Q) | None |
| *PRAMEF12* | rs199576535 | G/A (V341I) | None |
| *PLCB2* | rs200787930 | C/T (E1095K) | [Schizophrenia](http://www.ebi.ac.uk/gwas/search?query=Schizophrenia) (PMID: 25056061, PMID: 21926974) |
| *CXCL8* | rs188378669 | G/T | None |
| *TMOD4* | rs115287176 | G/A (R277W) | None |
| *ADGRL3* | rs192210727 | G/T (R580I) | [Postbronchodilator FEV1/FVC ratio in chronic obstructive pulmonary disease](http://www.ebi.ac.uk/gwas/search?query=Post%20bronchodilator%20FEV1/FVC%20ratio%20in%20COPD) (PMID: 26634245), [response to antipsychotic treatment in schizophrenia](http://www.ebi.ac.uk/gwas/search?query=Response%20to%20antipsychotic%20treatment%20in%20schizophrenia%20(working%20memory)) (PMID: 21107309), [partial epilepsies](http://www.ebi.ac.uk/gwas/search?query=Partial%20epilepsies) (PMID: 20522523) |
| *ZNF77* | rs146879198 | G/A (R340*) | None |
| *COL6A3* | rs146092501 | C/T (E1386K) | [Aging](http://www.ebi.ac.uk/gwas/search?query=Aging%20(time%20to%20event)) (PMID: 21782286), [prostate cancer](http://www.ebi.ac.uk/gwas/search?query=Prostate%20cancer) (PMID: 21743467) |
| *IQCF1* | rs200134435 | G/A (R103W) | [Amyotrophic lateral sclerosis](http://www.ebi.ac.uk/gwas/search?query=Amyotrophic%20lateral%20sclerosis%20(sporadic)) (PMID: 24529757) |
| *CYP4F12* | rs609636 | G/A (D76N) | None |
| *LPL* | rs15285  rs13702  rs326  rs301  rs328 | G/A  A/G  A/G  T/C  C/G (S474*) | [**HDL-cholesterol**](http://www.ebi.ac.uk/gwas/search?query=HDL%20cholesterol) (PMID: 26780889, PMID: 26763881, PMID: 26582766, PMID: 23726366, PMID: 18193044), [triglyceride](http://www.ebi.ac.uk/gwas/search?query=Triglyceride%20levels) (PMID: 26763881, PMID: 26582766, PMID: 23726366, PMID: 18193046, PMID: 22171074, PMID: 18193044), [triglycerides–blood pressure, [**HDL-cholesterol**–triglycerides](http://www.ebi.ac.uk/gwas/search?query=HDL%20Cholesterol%20-%20Triglycerides%20(HDLC-TG))](http://www.ebi.ac.uk/gwas/search?query=Triglycerides-Blood%20Pressure%20(TG-BP)), and [metabolic syndrome](http://www.ebi.ac.uk/gwas/search?query=Metabolic%20syndrome%20(bivariate%20traits)) (PMID: 21386085), [lipid traits](http://www.ebi.ac.uk/gwas/search?query=Lipid%20traits) (PMID: 24386095) |
| 6p21.3 | rs7773955  rs2517518 | C/T  G/A | [Age-related macular degeneration](http://www.ebi.ac.uk/gwas/search?query=Age-related%20macular%20degeneration) (PMID: 22694956), [nevirapine-induced rash](http://www.ebi.ac.uk/gwas/search?query=Nevirapine-induced%20rash) (PMID: 21810746), [chronic lymphocytic leukemia](http://www.ebi.ac.uk/gwas/search?query=Chronic%20lymphocytic%20leukemia) (PMID: 21131588), [nasopharyngeal carcinoma](http://www.ebi.ac.uk/gwas/search?query=Nasopharyngeal%20carcinoma) (PMID: 19664746) |
| 8p21.3 | rs2197089  rs2083637  rs1441756  rs17482753  rs10096633  rs10503669  rs12678919  rs7016880 | C/T  T/C  T/G  G/T  C/T  C/A  A/G  G/C | [**HDL-cholesterol**](http://www.ebi.ac.uk/gwas/search?query=HDL%20cholesterol) (PMID: 26780889, PMID: 26763881, PMID: 19060911, PMID: 20031538, PMID: 21347282, PMID: 21909109, PMID: 18193043, PMID: 26582766, PMID: 24097068), [triglyceride levels](http://www.ebi.ac.uk/gwas/search?query=Triglyceride%20levels) (PMID: 26763881, PMID: 20139978, PMID: 19060911, PMID: 21909109, PMID: 18193043, PMID: 26582766, PMID: 24097068), [erythrocyte cadmium concentration in never-smokers](http://www.ebi.ac.uk/gwas/search?query=Erythrocyte%20cadmium%20concentration%20in%20never%20smokers) (PMID: 27005419), [response to lithium treatment in bipolar disorder](http://www.ebi.ac.uk/gwas/search?query=Response%20to%20lithium%20treatment%20in%20bipolar%20disorder) (PMID: 26806518), [metabolic syndrome](http://www.ebi.ac.uk/gwas/search?query=Metabolic%20syndrome%20(bivariate%20traits)) (PMID: 21386085, PMID: 20694148), [waist circumference and related phenotypes](http://www.ebi.ac.uk/gwas/search?query=Waist%20circumference%20and%20related%20phenotypes) (PMID: 18454146), [lipid traits](http://www.ebi.ac.uk/gwas/search?query=Lipid%20traits) (PMID: 24023261), [metabolic traits](http://www.ebi.ac.uk/gwas/search?query=Metabolic%20traits) (PMID: 19060910), [hypertriglyceridemia](http://www.ebi.ac.uk/gwas/search?query=Hypertriglyceridemia) (PMID: 20657596) |
| LOC101928635 | rs1532085  rs10468017  rs2043085  rs4775041 | A/G  C/T  A/G  G/C | [**HDL-cholesterol**](http://www.ebi.ac.uk/gwas/search?query=HDL%20cholesterol) (PMID: 26582766, PMID: 24097068, PMID: 18193043), [triglycerides](http://www.ebi.ac.uk/gwas/search?query=Triglycerides) (PMID: 26582766, PMID: 24097068, PMID: 18193043), [total cholesterol](http://www.ebi.ac.uk/gwas/search?query=Cholesterol,%20total) (PMID: 26582766, PMID: 24097068), [age-related macular degeneration](http://www.ebi.ac.uk/gwas/search?query=Advanced%20age-related%20macular%20degeneration) (PMID: 26691988, PMID: 21665990), [stroke](http://www.ebi.ac.uk/gwas/search?query=Stroke) (PMID: 26089329), [red blood cell traits](http://www.ebi.ac.uk/gwas/search?query=Red%20blood%20cell%20traits) (PMID: 23222517), [metabolite levels](http://www.ebi.ac.uk/gwas/search?query=Metabolite%20levels) (PMID: 22916037, PMID: 24816252, PMID: 19043545), [phospholipid levels](http://www.ebi.ac.uk/gwas/search?query=Phospholipid%20levels%20(plasma)) (PMID: 22359512), [cardiovascular disease risk factors](http://www.ebi.ac.uk/gwas/search?query=Cardiovascular%20disease%20risk%20factors) (PMID: 21943158), [metabolic syndrome](http://www.ebi.ac.uk/gwas/search?query=Metabolic%20syndrome%20(bivariate%20traits)) (PMID: 21386085), [lipid traits](http://www.ebi.ac.uk/gwas/search?query=Lipid%20traits) (PMID: 24386095) |
| *NAA25* | rs12231744 | C/T (R876K) | [Hypothyroidism](http://www.ebi.ac.uk/gwas/search?query=Hypothyroidism) (PMID: 22493691), [upper aerodigestive tract cancers](http://www.ebi.ac.uk/gwas/search?query=Upper%20aerodigestive%20tract%20cancers) (PMID: 21437268), [type 1 diabetes](http://www.ebi.ac.uk/gwas/search?query=Type%201%20diabetes) (PMID: 18978792, PMID: 17554300, PMID: 17554260) |
| *BUD13* | rs10790162 | G/A | [**HDL-cholesterol**](http://www.ebi.ac.uk/gwas/search?query=HDL%20cholesterol) (PMID: 26780889), [triglyceride levels](http://www.ebi.ac.uk/gwas/search?query=Triglyceride%20levels) (PMID: 26763881), [**HDL-cholesterol**–triglycerides, [waist circumference–triglycerides, and](http://www.ebi.ac.uk/gwas/search?query=Waist%20Circumference%20-%20Triglycerides%20(WC-TG)) [metabolic syndrome](http://www.ebi.ac.uk/gwas/search?query=Metabolic%20syndrome)](http://www.ebi.ac.uk/gwas/search?query=HDL%20Cholesterol%20-%20Triglycerides%20(HDLC-TG)) (PMID: 21386085), [postbronchodilator FEV1/FVC ratio](http://www.ebi.ac.uk/gwas/search?query=Post%20bronchodilator%20FEV1/FVC%20ratio) (PMID: 26634245), [severe influenza A (H1N1) infection](http://www.ebi.ac.uk/gwas/search?query=Severe%20influenza%20A%20(H1N1)%20infection) (PMID: 26379185), [manganese levels](http://www.ebi.ac.uk/gwas/search?query=Manganese%20levels) (PMID: 26025379) |
| *PTCH2* | rs147284320 | C/T (V503I) | None |
| *ZPR1* | rs964184  rs2075290 | C/G  T/C | [**HDL-cholesterol**–triglycerides, [waist circumference–triglycerides](http://www.ebi.ac.uk/gwas/search?query=Waist%20Circumference%20-%20Triglycerides%20(WC-TG)), and [metabolic syndrome](http://www.ebi.ac.uk/gwas/search?query=Metabolic%20syndrome)](http://www.ebi.ac.uk/gwas/search?query=HDL%20Cholesterol%20-%20Triglycerides%20(HDLC-TG)) (PMID: 21386085), [total cholesterol](http://www.ebi.ac.uk/gwas/search?query=Cholesterol,%20total) (PMID: 26780889, PMID: 25961943), [triglycerides](http://www.ebi.ac.uk/gwas/search?query=Triglycerides) (PMID: 26780889), [LDL-cholesterol](http://www.ebi.ac.uk/gwas/search?query=LDL%20cholesterol) (PMID: 25961943), [postprandial triglyceride response to high-fat diet meal](http://www.ebi.ac.uk/gwas/search?query=Postprandial%20triglyceride%20response%20to%20high%20fat%20diet%20meal) (PMID: 26256467) |
| *OR52I1* | rs200585398 | A/G (M167V) | None |
| *ABCA1* | rs1883025  rs2066714 | G/A  C/T (M883I) | [**HDL-cholesterol**](http://www.ebi.ac.uk/gwas/search?query=HDL%20cholesterol) (PMID: 26780889, PMID: 24097068, PMID: 20686565), [**low HDL-cholesterol levels**](http://www.ebi.ac.uk/gwas/search?query=Low%20high%20density%20lipoprotein%20cholesterol%20levels) (PMID: 26879886), [total cholesterol](http://www.ebi.ac.uk/gwas/search?query=Cholesterol,%20total) (PMID: 24097068, PMID: 20686565), [response of total cholesterol to fenofibrate](http://www.ebi.ac.uk/gwas/search?query=Response%20to%20fenofibrate%20(total%20cholesterol%20levels))  (PMID: 27002377), [posterior cortical atrophy and Alzheimer’s disease](http://www.ebi.ac.uk/gwas/search?query=Posterior%20cortical%20atrophy%20and%20Alzheimer) (PMID: 26993346), [advanced age-related macular degeneration](http://www.ebi.ac.uk/gwas/search?query=Advanced%20age-related%20macular%20degeneration) (PMID: 26691988), [metabolic syndrome](http://www.ebi.ac.uk/gwas/search?query=Metabolic%20syndrome) (PMID: 22399527) |
| *ATXN2* | rs7969300 | T/C (N248S) | [Glaucoma](http://www.ebi.ac.uk/gwas/search?query=Glaucoma%20(high%20intraocular%20pressure)) (PMID: 26752265), [systemic lupus erythematosus](http://www.ebi.ac.uk/gwas/search?query=Systemic%20lupus%20erythematosus) (PMID: 26502338) |
| 11q23.3 | rs9326246  rs7350481 | G/C  C/T | [**HDL-cholesterol**](http://www.ebi.ac.uk/gwas/search?query=HDL%20cholesterol) (PMID: 26780889), [triglycerides](http://www.ebi.ac.uk/gwas/search?query=Triglycerides) (PMID: 26780889, PMID: 20139978), [total cholesterol](http://www.ebi.ac.uk/gwas/search?query=Cholesterol,%20total) (PMID: 26780889), [aggressiveness in attention deficit–hyperactivity disorder](http://www.ebi.ac.uk/gwas/search?query=Aggressiveness%20in%20attention%20deficit%20hyperactivity%20disorder) (PMID: 27021288), [parental extreme longevity](http://www.ebi.ac.uk/gwas/search?query=Parental%20extreme%20longevity%20(95%20years%20and%20older)) (PMID: 27015805), [hematologic and biochemical traits](http://www.ebi.ac.uk/gwas/search?query=Hematological%20and%20biochemical%20traits) (PMID: 20139978) |
| *TCF19* | rs61733202 | G/A (G26R) | [Type 2 diabetes](http://www.ebi.ac.uk/gwas/search?query=Type%202%20diabetes) (PMID: 24509480), [chronic hepatitis B infection](http://www.ebi.ac.uk/gwas/search?query=Chronic%20hepatitis%20B%20infection) (PMID: 25802187, PMID: 23760081), [marginal zone lymphoma](http://www.ebi.ac.uk/gwas/search?query=Marginal%20zone%20lymphoma) (PMID: 25569183), [multiple myeloma](http://www.ebi.ac.uk/gwas/search?query=Multiple%20myeloma) (PMID: 23955597) |
| *OAS3* | rs2072134 | C/T | [**HDL-cholesterol**](http://www.ebi.ac.uk/gwas/search?query=HDL%20cholesterol) (PMID: 21909109), [response of adiponectin to fenofibrate](http://www.ebi.ac.uk/gwas/search?query=Response%20to%20fenofibrate%20(adiponectin%20levels))  (PMID: 23149075), [alcohol consumption](http://www.ebi.ac.uk/gwas/search?query=Alcohol%20consumption) (PMID: 21270382) |
| *LOC554223* | rs1610640 | A/G | None |
| *HLA-B* | rs1058026 | T/G | [Crohn’s disease](http://www.ebi.ac.uk/gwas/search?query=Crohn) (PMID: 26891255), [psoriatic arthritis](http://www.ebi.ac.uk/gwas/search?query=Psoriatic%20arthritis) and [psoriasis vulgaris](http://www.ebi.ac.uk/gwas/search?query=Psoriasis%20vulgaris) (PMID: 26626624), [systemic lupus erythematosus](http://www.ebi.ac.uk/gwas/search?query=Systemic%20lupus%20erythematosus) (PMID: 26606652), [setpoint viral load in HIV-1 infection](http://www.ebi.ac.uk/gwas/search?query=Setpoint%20viral%20load%20in%20HIV-1%20infection) (PMID: 26553974) |
| *PLCD1* | rs147186786 | C/T (R268Q) | None |
| *DENND1C* | rs200449136 | G/A | [Adverse response to chemotherapy](http://www.ebi.ac.uk/gwas/search?query=Adverse%20response%20to%20chemotherapy%20(neutropenia/leucopenia)%20(cyclophosphamide)) (PMID: 23648065) |
| *LOC101929163* | rs3129945 | G/A | [Triglycerides](http://www.ebi.ac.uk/gwas/search?query=Triglycerides) (PMID: 25961943), [drug-induced liver injury](http://www.ebi.ac.uk/gwas/search?query=Drug-induced%20liver%20injury) (PMID: 26959717), [Epstein-Barr virus nuclear antigen 1 IgG levels](http://www.ebi.ac.uk/gwas/search?query=Epstein%20Barr%20virus%20nuclear%20antigen%201%20IgG%20levels) (PMID: 26819262), [schizophrenia](http://www.ebi.ac.uk/gwas/search?query=Schizophrenia) (PMID: 26198764), [type 1 diabetes and autoimmune thyroid diseases](http://www.ebi.ac.uk/gwas/search?query=Type%201%20diabetes%20and%20autoimmune%20thyroid%20diseases) (PMID: 25936594) |
| *ANKRD11* | rs139088883 | G/A (A1840V) | [Multiple myeloma](http://www.ebi.ac.uk/gwas/search?query=Multiple%20myeloma%20(IgH%20translocation)) (PMID: 23502783) |
| *BTNL2* | rs2076528  rs3763315  rs41441651  rs28362675  rs41417449  rs78587369  rs34423804  rs3806156 | T/G  G/T  C/T (D336N)  C/A (E454*)  T/C (M295V)  G/A (T165I)  T/A (V283D)  G/T | [Epstein-Barr virus nuclear antigen 1 IgG levels](http://www.ebi.ac.uk/gwas/search?query=Epstein%20Barr%20virus%20nuclear%20antigen%201%20IgG%20levels) (PMID: 26819262), [*Staphylococcus aureus* infection](http://www.ebi.ac.uk/gwas/search?query=Staphylococcus%20aureus%20infection) (PMID: 26450422), [thionamide-induced agranulocytosis in Graves’ disease](http://www.ebi.ac.uk/gwas/search?query=Thionamide-induced%20agranulocytosis%20in%20Graves) (PMID: 26151496), [HIV-1 viral setpoint](http://www.ebi.ac.uk/gwas/search?query=HIV-1%20viral%20setpoint) (PMID: 26039976), [type 1 diabetes and autoimmune thyroid diseases](http://www.ebi.ac.uk/gwas/search?query=Type%201%20diabetes%20and%20autoimmune%20thyroid%20diseases) (PMID: 25936594), [vitiligo](http://www.ebi.ac.uk/gwas/search?query=Vitiligo) (PMID: 20410501) |
| *APOE* | rs7412 | C/T (R176C) | [LDL-cholesterol](http://www.ebi.ac.uk/gwas/search?query=LDL%20cholesterol) (PMID: 23067351), [response of [LDL-cholesterol](http://www.ebi.ac.uk/gwas/search?query=LDL%20cholesterol) to statin therapy](http://www.ebi.ac.uk/gwas/search?query=Response%20to%20statin%20therapy%20(LDL-C)) (PMID: 22331829), [total cholesterol](http://www.ebi.ac.uk/gwas/search?query=Cholesterol,%20total) (PMID: 25961943), [parental longevity](http://www.ebi.ac.uk/gwas/search?query=Parental%20longevity%20(combined%20parental%20age%20at%20death)) (PMID: 27015805), [posterior cortical atrophy and Alzheimer’s disease](http://www.ebi.ac.uk/gwas/search?query=Posterior%20cortical%20atrophy%20and%20Alzheimer) (PMID: 26993346), [Alzheimer’s disease and age of onset](http://www.ebi.ac.uk/gwas/search?query=Alzheimer%20disease%20and%20age%20of%20onset) (PMID: 26830138), [body fat percentage](http://www.ebi.ac.uk/gwas/search?query=Body%20fat%20percentage) (PMID: 26833246), [ideal cardiovascular health](http://www.ebi.ac.uk/gwas/search?query=Ideal%20cardiovascular%20health%20(clinical%20and%20behavioural)) (PMID: 27179730), [lipid traits](http://www.ebi.ac.uk/gwas/search?query=Lipid%20traits) (PMID: 24023260) |
| *CD36* | rs75326924 | C/T (P90S) | [**HDL-cholesterol**](http://www.ebi.ac.uk/gwas/search?query=HDL%20cholesterol) (PMID: 23726366), [response to fenofibrate](http://www.ebi.ac.uk/gwas/search?query=Response%20to%20fenofibrate) (PMID: 22890011), [mean platelet volume](http://www.ebi.ac.uk/gwas/search?query=Mean%20platelet%20volume) (PMID: 22423221), [left ventricular mass](http://www.ebi.ac.uk/gwas/search?query=Left%20ventricular%20mass) (PMID: 19454037) |
| *NOS3* | rs7792133 | G/A (R665H) | [Coronary artery disease](http://www.ebi.ac.uk/gwas/search?query=Coronary%20artery%20disease) (PMID: 26343387), [eyebrow thickness](http://www.ebi.ac.uk/gwas/search?query=Eyebrow%20thickness) (PMID: 26926045) |
| *ACE* | rs4314 | C/T (R561W) | [Cardiovascular disease and myocardial infarction in hypertension](http://www.ebi.ac.uk/gwas/search?query=Cardiovascular%20disease%20in%20hypertension%20(ACE%20inhibitor%20interaction)) (PMID: 26516778), [cough in response to angiotensin-converting enzyme inhibitor drugs](http://www.ebi.ac.uk/gwas/search?query=Cough%20in%20response%20to%20angiotensin-converting%20enzyme%20inhibitor%20drugs) (PMID: 26169577), [cerebrospinal fluid levels of Alzheimer’s disease–related proteins](http://www.ebi.ac.uk/gwas/search?query=Cerebrospinal%20fluid%20levels%20of%20Alzheimer) (PMID: 25340798), [blood metabolite ratios](http://www.ebi.ac.uk/gwas/search?query=Blood%20metabolite%20ratios) (PMID: 24816252) |
| *TICRR* | rs150565858 | G/A (R301Q) | [Resting oxygen saturation in chronic obstructive pulmonary disease](http://www.ebi.ac.uk/gwas/search?query=Resting%20oxygen%20saturation%20in%20chronic%20osbtructive%20pulmonary%20disease%20(pulse%20oxymetry)) (PMID: 24825563), [obesity-related traits](http://www.ebi.ac.uk/gwas/search?query=Obesity-related%20traits) (PMID: 23251661) |
| *HCG22* | rs3873352  rs2523849 | G/C  A/G | [Plasma omega-3 polyunsaturated fatty acid level](http://www.ebi.ac.uk/gwas/search?query=Plasma%20omega-3%20polyunsaturated%20fatty%20acid%20level%20(eicosapentaenoic%20acid)) (PMID: 26584805), [thionamide-induced agranulocytosis in Graves’ disease](http://www.ebi.ac.uk/gwas/search?query=Thionamide-induced%20agranulocytosis%20in%20Graves) (PMID: 26151496), [change in intraocular pressure in response to steroid treatment](http://www.ebi.ac.uk/gwas/search?query=Change%20in%20intraocular%20pressure%20in%20response%20to%20steroid%20treatment%20(triamcinolone%20acetonide)) (PMID: 25813999), body [height](http://www.ebi.ac.uk/gwas/search?query=Height) (PMID: 25429064), [hematology traits](http://www.ebi.ac.uk/gwas/search?query=Hematology%20traits) (PMID: 23263863) |
| *SKIV2L* | rs592229 | G/T | [Age-related macular degeneration](http://www.ebi.ac.uk/gwas/search?query=Advanced%20age-related%20macular%20degeneration) (PMID: 26691988, PMID: 23577725), [infant length](http://www.ebi.ac.uk/gwas/search?query=Infant%20length) (PMID: 25281659), [blood metabolite levels](http://www.ebi.ac.uk/gwas/search?query=Blood%20metabolite%20levels) (PMID: 24816252), [prostate cancer](http://www.ebi.ac.uk/gwas/search?query=Prostate%20cancer) (PMID: 23535732), [IgG glycosylation](http://www.ebi.ac.uk/gwas/search?query=IgG%20glycosylation) (PMID: 23382691) |
| *ZNF33B* | rs7914982 | T/C (H244R) | None |
| *PPP1R10* | rs3895681 | G/C | None |
| *CAT* | rs139421991 | G/A (R320Q) | [Cataracts in type 2 diabetes](http://www.ebi.ac.uk/gwas/search?query=Cataracts%20in%20type%202%20diabetes) (PMID: 23137000) |
| *TNC* | rs138406927 | C/T (A1096T) | [Developmental language disorder](http://www.ebi.ac.uk/gwas/search?query=Developmental%20language%20disorder%20(syntactic%20complexity)) (PMID: 27016271), [postbronchodilator FEV1 in chronic obstructive pulmonary disease](http://www.ebi.ac.uk/gwas/search?query=Post%20bronchodilator%20FEV1%20in%20COPD) (PMID: 26634245), [plasma omega-3 polyunsaturated fatty acid level](http://www.ebi.ac.uk/gwas/search?query=Plasma%20omega-3%20polyunsaturated%20fatty%20acid%20level%20(eicosapentaenoic%20acid)) (PMID: 26584805), [cerebral amyloid deposition in APOEε4 noncarriers](http://www.ebi.ac.uk/gwas/search?query=Cerebral%20amyloid%20deposition%20in%20APOEe4%20non-carriers%20(PET%20imaging)) (PMID: 26252872), [glucose homeostasis traits](http://www.ebi.ac.uk/gwas/search?query=Glucose%20homeostasis%20traits) (PMID: 25524916) |
| Associated with hypo–HDL-cholesterolemia | | | |
| *TRABD2B* | rs147317864 | C/T (A262T) | [Obesity-related traits](http://www.ebi.ac.uk/gwas/search?query=Obesity-related%20traits) (PMID: 23251661), [white matter integrity](http://www.ebi.ac.uk/gwas/search?query=White%20matter%20integrity) (PMID: 22425255) |

Data were obtained from GWAS Catalog (http://www.ebi.ac.uk/gwas), and phenotypes related to serum HDL-cholesterol are shown in bold. PMID, PubMed ID; chr., chromosome.
