# Supplementary material for: Identification of eight genetic variants as novel determinants of dyslipidemia in Japanese by exome-wide association studies
Source: Oncotarget. 2017 Apr 17;8(24):38950–61. doi: 10.18632/oncotarget.17159 (PMC5503585; doi:10.18632/oncotarget.17159)
Supplement: Supplementary file 17 [file oncotarget-08-38950-s017.docx]

**Supplementary Table 18.** Relation of genes, loci, and SNPs identified in the present analysis of serum LDL-cholesterol to phenotypes previously examined in GWASs.

| Gene  (or chr. locus) | SNP | Nucleotide  (amino acid)  substitution | Previously examined phenotypes |
| --- | --- | --- | --- |
| Associated with serum LDL-cholesterol and hyper–LDL-cholesterolemia | | | |
| 6p21.3 | rs2853969 | C/T | [Age-related macular degeneration](http://www.ebi.ac.uk/gwas/search?query=Age-related%20macular%20degeneration) (PMID: 22694956), [nevirapine-induced rash](http://www.ebi.ac.uk/gwas/search?query=Nevirapine-induced%20rash) (PMID: 21810746), [chronic lymphocytic leukemia](http://www.ebi.ac.uk/gwas/search?query=Chronic%20lymphocytic%20leukemia) (PMID: 21131588), [nasopharyngeal carcinoma](http://www.ebi.ac.uk/gwas/search?query=Nasopharyngeal%20carcinoma) (PMID: 19664746) |
| Associated with serum LDL-cholesterol | | | |
| *APOE* | rs7412  rs769449 | C/T (R176C)  G/A | [**LDL-cholesterol**](http://www.ebi.ac.uk/gwas/search?query=LDL%20cholesterol) (PMID: 23067351), total [cholesterol](http://www.ebi.ac.uk/gwas/search?query=Cholesterol,%20total) (PMID: 25961943), [lipid traits](http://www.ebi.ac.uk/gwas/search?query=Lipid%20traits) (PMID: 24023260), [**response of** [**LDL-cholesterol**](http://www.ebi.ac.uk/gwas/search?query=LDL%20cholesterol) **to statin therapy**](http://www.ebi.ac.uk/gwas/search?query=Response%20to%20statin%20therapy%20(LDL-C)) (PMID: 22331829), [parental longevity](http://www.ebi.ac.uk/gwas/search?query=Parental%20longevity%20(combined%20parental%20age%20at%20death)) (PMID: 27015805), [posterior cortical atrophy and Alzheimer’s disease](http://www.ebi.ac.uk/gwas/search?query=Posterior%20cortical%20atrophy%20and%20Alzheimer) (PMID: 26993346), [Alzheimer’s disease and age of onset](http://www.ebi.ac.uk/gwas/search?query=Alzheimer%20disease%20and%20age%20of%20onset) (PMID: 26830138), [body fat percentage](http://www.ebi.ac.uk/gwas/search?query=Body%20fat%20percentage) (PMID: 26833246), [ideal cardiovascular health](http://www.ebi.ac.uk/gwas/search?query=Ideal%20cardiovascular%20health%20(clinical%20and%20behavioural)) (PMID: 27179730), [cingulate cortical amyloid beta load](http://www.ebi.ac.uk/gwas/search?query=Cingulate%20cortical%20amyloid%20beta%20load) (PMID: 26421299), [cognitive decline (age-related)](http://www.ebi.ac.uk/gwas/search?query=Cognitive%20decline%20(age-related)) (PMID: 24468470), [Alzheimer’s disease biomarkers](http://www.ebi.ac.uk/gwas/search?query=Alzheimer) (PMID: 23562540), [C-reactive protein](http://www.ebi.ac.uk/gwas/search?query=C-reactive%20protein) (PMID: 18439548) |
| *APOC1* | rs445925 | C/T | [**Response of LDL-cholesterol to statins**](http://www.ebi.ac.uk/gwas/search?query=Response%20to%20statins%20(LDL%20cholesterol%20change)) (PMID: 25350695), [**response of LDL-cholesterol to fenofibrate**](http://www.ebi.ac.uk/gwas/search?query=Response%20to%20fenofibrate%20(LDL%20cholesterol%20levels)) (PMID: 27002377), [blood metabolite ratios](http://www.ebi.ac.uk/gwas/search?query=Blood%20metabolite%20ratios) (PMID: 24816252), [lipoprotein-associated phospholipase A_2_ activity and mass](http://www.ebi.ac.uk/gwas/search?query=Lipoprotein-associated%20phospholipase%20A2%20activity%20and%20mass) (PMID: 23118302), [apolipoprotein levels](http://www.ebi.ac.uk/gwas/search?query=Apolipoprotein%20Levels) (PMID: 23031429), [Alzheimer’s disease and age of onset](http://www.ebi.ac.uk/gwas/search?query=Alzheimer%20disease%20and%20age%20of%20onset) (PMID: 26830138), [body fat percentage](http://www.ebi.ac.uk/gwas/search?query=Body%20fat%20percentage) (PMID: 26833246), [ideal cardiovascular health](http://www.ebi.ac.uk/gwas/search?query=Ideal%20cardiovascular%20health%20(clinical)) (PMID: 27179730), [coronary artery disease](http://www.ebi.ac.uk/gwas/search?query=Coronary%20artery%20disease) (PMID: 26343387) |
| *APOB* | rs13306206  rs13306194 | G/A (P955S)  G/A (R532W) | [**LDL-cholesterol**](http://www.ebi.ac.uk/gwas/search?query=LDL%20cholesterol) (PMID: 26780889, PMID: 26582766, PMID: 25961943), [total cholesterol](http://www.ebi.ac.uk/gwas/search?query=Cholesterol,%20total) (PMID: 26582766, PMID: 25961943) |
| *PCSK9* | rs151193009 | C/T (R93C) | [**LDL-cholesterol**](http://www.ebi.ac.uk/gwas/search?query=LDL%20cholesterol) (PMID: 26582766), total [cholesterol](http://www.ebi.ac.uk/gwas/search?query=Cholesterol,%20total) (PMID: 26582766, PMID: 25961943), [coronary artery disease](http://www.ebi.ac.uk/gwas/search?query=Coronary%20artery%20disease) (PMID: 26343387), [myocardial infarction](http://www.ebi.ac.uk/gwas/search?query=Myocardial%20infarction) (PMID: 26343387) |
| *PSRC1* | rs599839 | A/G | [**LDL-cholesterol**](http://www.ebi.ac.uk/gwas/search?query=LDL%20cholesterol) (PMID: 25961943, PMID: 18262040, PMID: 18193043), [total cholesterol](http://www.ebi.ac.uk/gwas/search?query=Cholesterol,%20total) (PMID: 25961943, PMID: 24886709), [metabolite levels](http://www.ebi.ac.uk/gwas/search?query=Metabolite%20levels) (PMID: 21909109), [coronary heart disease](http://www.ebi.ac.uk/gwas/search?query=Coronary%20heart%20disease) (PMID: 21378988), [lipoprotein-associated phospholipase A_2_ activity and mass](http://www.ebi.ac.uk/gwas/search?query=Lipoprotein-associated%20phospholipase%20A2%20activity%20and%20mass) (PMID: 20442857) |
| *CELSR2* | rs629301  rs12740374  rs646776 | A/C  G/T  A/G | [**LDL-cholesterol**](http://www.ebi.ac.uk/gwas/search?query=LDL%20cholesterol) (PMID: 26780889, PMID: 24097068, PMID: 20686565, PMID: 21347282, PMID: 19060906, PMID: 25961943), [total cholesterol](http://www.ebi.ac.uk/gwas/search?query=Cholesterol,%20total) (PMID: 26780889, PMID: 25961943, PMID: 24097068, PMID: 20686565), [coronary artery disease](http://www.ebi.ac.uk/gwas/search?query=Coronary%20artery%20disease) (PMID: 26343387, PMID: 21378988, PMID: 21239051), [myocardial infarction](http://www.ebi.ac.uk/gwas/search?query=Myocardial%20infarction) (PMID: 26343387), [lipoprotein-associated phospholipase A_2_ activity and mass](http://www.ebi.ac.uk/gwas/search?query=Lipoprotein-associated%20phospholipase%20A2%20activity%20and%20mass) (PMID: 23118302), [progranulin levels](http://www.ebi.ac.uk/gwas/search?query=Progranulin%20levels) (PMID: 21087763) |
| 1p13.3 | rs602633 | C/A | [**LDL-cholesterol**](http://www.ebi.ac.uk/gwas/search?query=LDL%20cholesterol) (PMID: 26780889), [total cholesterol](http://www.ebi.ac.uk/gwas/search?query=Cholesterol,%20total) (PMID: 26780889), [glomerular filtration rate](http://www.ebi.ac.uk/gwas/search?query=Glomerular%20filtration%20rate%20(creatinine)) (PMID: 26831199), [Alzheimer’s disease and age of onset](http://www.ebi.ac.uk/gwas/search?query=Alzheimer%20disease%20and%20age%20of%20onset) (PMID: 26830138), [response to cognitive-behavioral therapy in anxiety disorder](http://www.ebi.ac.uk/gwas/search?query=Response%20to%20cognitive-behavioural%20therapy%20in%20anxiety%20disorder) (PMID: 26989097) |
| *ABO* | rs1053878 | G/A (P156L) | [**LDL-cholesterol**](http://www.ebi.ac.uk/gwas/search?query=LDL%20cholesterol) (PMID: 26582766), [total cholesterol](http://www.ebi.ac.uk/gwas/search?query=Cholesterol,%20total) (PMID: 26582766), [low vWF levels](http://www.ebi.ac.uk/gwas/search?query=Low%20vWF%20levels) (PMID: 26486471), [coronary artery disease](http://www.ebi.ac.uk/gwas/search?query=Coronary%20artery%20disease) (PMID: 26343387), [thrombosis](http://www.ebi.ac.uk/gwas/search?query=Thrombosis) (PMID: 26908601) |
| 9q34.2 | rs651007  rs579459  rs635634  rs507666 | G/A  T/C  G/A  G/A | [**LDL-cholesterol**](http://www.ebi.ac.uk/gwas/search?query=LDL%20cholesterol) (PMID: 26582766, PMID: 20686565), [total cholesterol](http://www.ebi.ac.uk/gwas/search?query=Cholesterol,%20total) (PMID: 26582766, PMID: 20686565, PMID: 25961943), [ADAMTS13 activity](http://www.ebi.ac.uk/gwas/search?query=ADAMTS13%20activity) (PMID: 25934476), [postbronchodilator FEV1](http://www.ebi.ac.uk/gwas/search?query=Post%20bronchodilator%20FEV1) and [FEV1/FVC ratio](http://www.ebi.ac.uk/gwas/search?query=Post%20bronchodilator%20FEV1/FVC%20ratio) (PMID: 26634245), [iron status biomarkers (ferritin levels)](http://www.ebi.ac.uk/gwas/search?query=Iron%20status%20biomarkers%20(ferritin%20levels)) (PMID: 25352340), [blood metabolite levels](http://www.ebi.ac.uk/gwas/search?query=Blood%20metabolite%20levels) (PMID: 24816252, PMID: 21909109), [serum alkaline phosphatase levels](http://www.ebi.ac.uk/gwas/search?query=Serum%20alkaline%20phosphatase%20levels) (PMID: 24094242), [end-stage coagulation](http://www.ebi.ac.uk/gwas/search?query=End-stage%20coagulation) (PMID: 23381943), [urinary metabolites](http://www.ebi.ac.uk/gwas/search?query=Urinary%20metabolites%20(H-NMR%20features)) (PMID: 24586186), [red blood cell traits](http://www.ebi.ac.uk/gwas/search?query=Red%20blood%20cell%20traits) (PMID: 23222517), [lipid traits](http://www.ebi.ac.uk/gwas/search?query=Lipid%20traits) (PMID: 24386095), [soluble ICAM-1](http://www.ebi.ac.uk/gwas/search?query=Soluble%20ICAM-1) (PMID: 21533024, PMID: 18604267) |
| *MUC22* | rs117024916 | A/G (T71A) | [Parental longevity](http://www.ebi.ac.uk/gwas/search?query=Parental%20longevity%20(mother) (PMID: 27015805), [plasma omega-3 polyunsaturated fatty acid level](http://www.ebi.ac.uk/gwas/search?query=Plasma%20omega-3%20polyunsaturated%20fatty%20acid%20level%20(eicosapentaenoic%20acid)) (PMID: 26584805), [severe influenza A (H1N1) infection](http://www.ebi.ac.uk/gwas/search?query=Severe%20influenza%20A%20(H1N1)%20infection) (PMID: 26379185), [thionamide-induced agranulocytosis in Graves’ disease](http://www.ebi.ac.uk/gwas/search?query=Thionamide-induced%20agranulocytosis%20in%20Graves) (PMID: 26151496), [change in intraocular pressure in response to steroid treatment](http://www.ebi.ac.uk/gwas/search?query=Change%20in%20intraocular%20pressure%20in%20response%20to%20steroid%20treatment%20(triamcinolone%20acetonide))  (PMID: 25813999) |
| *VARS* | rs11751198  rs5030798 | G/A  C/T (V1055I) | None |
| *CCHCR1* | rs147733073 | C/G (H486Q) | [Multiple myeloma](http://www.ebi.ac.uk/gwas/search?query=Multiple%20myeloma) (PMID: 23955597), [hematology traits](http://www.ebi.ac.uk/gwas/search?query=Hematology%20traits) (PMID: 23263863), [chronic obstructive pulmonary disease–related biomarkers](http://www.ebi.ac.uk/gwas/search?query=Chronic%20obstructive%20pulmonary%20disease-related%20biomarkers) (PMID: 23144326), [Stevens-Johnson syndrome and toxic epidermal necrolysis (SJS-TEN)](http://www.ebi.ac.uk/gwas/search?query=Stevens-Johnson%20syndrome%20and%20toxic%20epidermal%20necrolysis%20(SJS-TEN)) (PMID: 21912425), [nevirapine-induced rash](http://www.ebi.ac.uk/gwas/search?query=Nevirapine-induced%20rash) (PMID: 21810746) |
| 6p21.3 | rs12210887  rs2596574 | G/T  G/A | [Age-related macular degeneration](http://www.ebi.ac.uk/gwas/search?query=Age-related%20macular%20degeneration) (PMID: 22694956), [nevirapine-induced rash](http://www.ebi.ac.uk/gwas/search?query=Nevirapine-induced%20rash) (PMID: 21810746), [chronic lymphocytic leukemia](http://www.ebi.ac.uk/gwas/search?query=Chronic%20lymphocytic%20leukemia) (PMID: 21131588), [nasopharyngeal carcinoma](http://www.ebi.ac.uk/gwas/search?query=Nasopharyngeal%20carcinoma) (PMID: 19664746) |
| *MSH5* | rs11754464 | C/T | [Clozapine-induced agranulocytosis/granulocytopenia in treatment-resistant schizophrenia](http://www.ebi.ac.uk/gwas/search?query=Clozapine-induced%20agranulocytosis/granulocytopenia%20in%20treatment-resistant%20schizophrenia) (PMID: 26876947), [systemic lupus erythematosus](http://www.ebi.ac.uk/gwas/search?query=Systemic%20lupus%20erythematosus) (PMID: 26316170), [cutaneous lupus erythematosus](http://www.ebi.ac.uk/gwas/search?query=Cutaneous%20lupus%20erythematosus) (PMID: 25827949), [ulcerative colitis](http://www.ebi.ac.uk/gwas/search?query=Ulcerative%20colitis) (PMID: 24837172), [lung cancer](http://www.ebi.ac.uk/gwas/search?query=Lung%20cancer) (PMID: 18978787) |
| *PRRC2A* | rs11538264 | G/A (V1774M) | Body [height](http://www.ebi.ac.uk/gwas/search?query=Height) (PMID: 25282103), [blood metabolite ratios](http://www.ebi.ac.uk/gwas/search?query=Blood%20metabolite%20ratios) (PMID: 24816252), [psychosis](http://www.ebi.ac.uk/gwas/search?query=Psychosis%20(atypical)) (PMID: 24132900), [schizophrenia](http://www.ebi.ac.uk/gwas/search?query=Schizophrenia) (PMID: 23894747), [menopause](http://www.ebi.ac.uk/gwas/search?query=Menopause%20(age%20at%20onset))  (PMID: 23307926) |
| *FAM65B* | rs150142878 | C/T (R371Q) | [Postbronchodilator FEV1/FVC ratio](http://www.ebi.ac.uk/gwas/search?query=Post%20bronchodilator%20FEV1/FVC%20ratio) (PMID: 26634245), [diisocyanate-induced asthma](http://www.ebi.ac.uk/gwas/search?query=Diisocyanate-induced%20asthma) (PMID: 25918132), [electroencephalographic traits in alcoholism](http://www.ebi.ac.uk/gwas/search?query=Electroencephalographic%20traits%20in%20alcoholism) (PMID: 22554406) |
| *HSPA1B* | rs6457452 | C/T | [IgG glycosylation](http://www.ebi.ac.uk/gwas/search?query=IgG%20glycosylation) (PMID: 23382691), [HIV-1 control](http://www.ebi.ac.uk/gwas/search?query=HIV-1%20control) (PMID: 20041166) |
| *LY6G6C* | rs117894946 | G/C (G75A) | None |
| *C6orf48* | rs11968400 | C/T | [Schizophrenia](http://www.ebi.ac.uk/gwas/search?query=Schizophrenia) (PMID: 26198764), [thionamide-induced agranulocytosis in Graves’ disease](http://www.ebi.ac.uk/gwas/search?query=Thionamide-induced%20agranulocytosis%20in%20Graves) (PMID: 26151496), [IgG glycosylation](http://www.ebi.ac.uk/gwas/search?query=IgG%20glycosylation) (PMID: 23382691), [HIV-1 control](http://www.ebi.ac.uk/gwas/search?query=HIV-1%20control) (PMID: 20041166) |
| *KIAA0319* | rs4576240 | G/T (P142T) | [Radiation response](http://www.ebi.ac.uk/gwas/search?query=Radiation%20response) (PMID: 20923822) |
| *ZSCAN31* | rs6922302 | C/G (P128A) | [Schizophrenia](http://www.ebi.ac.uk/gwas/search?query=Schizophrenia) (PMID: 26198764), [pulmonary function](http://www.ebi.ac.uk/gwas/search?query=Pulmonary%20function) (PMID: 21946350) |
| *NEU1* | rs13118 | T/A | [Schizophrenia](http://www.ebi.ac.uk/gwas/search?query=Schizophrenia) (PMID: 26198764) |
| *ZSCAN26* | rs76463649 | A/G (N15S) | None |
| *LY6G6F* | rs17200983  rs9267546  rs9267547 | C/A (P34Q)  G/A  G/A (A107T) | None |
| 6p22.1 | rs3129029 | A/C | [Parental extreme longevity](http://www.ebi.ac.uk/gwas/search?query=Parental%20extreme%20longevity%20(95%20years%20and%20older)) (PMID: 27015805), [chronic lymphocytic leukemia](http://www.ebi.ac.uk/gwas/search?query=Chronic%20lymphocytic%20leukemia) (PMID: 26956414), [glomerular filtration rate](http://www.ebi.ac.uk/gwas/search?query=Glomerular%20filtration%20rate%20(creatinine)) (PMID: 26831199), [platelet count](http://www.ebi.ac.uk/gwas/search?query=Platelet%20count) (PMID: 26805783), [late-onset myasthenia gravis](http://www.ebi.ac.uk/gwas/search?query=Late-onset%20myasthenia%20gravis) (PMID: 26562150) |
| *TNXB* | rs140770834  rs11751545 | C/G (L2271V)  A/C | [Systemic lupus erythematosus](http://www.ebi.ac.uk/gwas/search?query=Systemic%20lupus%20erythematosus) (PMID: 26502338, PMID: 26316170), [schizophrenia](http://www.ebi.ac.uk/gwas/search?query=Schizophrenia) (PMID: 26198764), [type 1 diabetes and autoimmune thyroid diseases](http://www.ebi.ac.uk/gwas/search?query=Type%201%20diabetes%20and%20autoimmune%20thyroid%20diseases) (PMID: 25936594), [atopic dermatitis](http://www.ebi.ac.uk/gwas/search?query=Atopic%20dermatitis) (PMID: 25574825) |
| *ABCF1* | rs4148249 | C/A | [Mild influenza (H1N1) infection](http://www.ebi.ac.uk/gwas/search?query=Mild%20influenza%20(H1N1)%20infection) (PMID: 26379185) |
| Associated with hyper–LDL-cholesterolemia | | | |
| 6p22.1 | rs7771335  rs1233399 | A/G  C/T | [Parental extreme longevity](http://www.ebi.ac.uk/gwas/search?query=Parental%20extreme%20longevity%20(95%20years%20and%20older)) (PMID: 27015805), [chronic lymphocytic leukemia](http://www.ebi.ac.uk/gwas/search?query=Chronic%20lymphocytic%20leukemia) (PMID: 26956414), [glomerular filtration rate](http://www.ebi.ac.uk/gwas/search?query=Glomerular%20filtration%20rate%20(creatinine)) (PMID: 26831199), [platelet count](http://www.ebi.ac.uk/gwas/search?query=Platelet%20count) (PMID: 26805783), [late-onset myasthenia gravis](http://www.ebi.ac.uk/gwas/search?query=Late-onset%20myasthenia%20gravis) (PMID: 26562150) |
| *C21orf59* | rs76974938 | C/T (D67N) | None |
| *MOG* | rs2071653 | C/T | [Platelet count](http://www.ebi.ac.uk/gwas/search?query=Platelet%20count) (PMID: 26805783), [migraine without aura](http://www.ebi.ac.uk/gwas/search?query=Migraine%20without%20aura) (PMID: 23793025), [pulmonary function](http://www.ebi.ac.uk/gwas/search?query=Pulmonary%20function%20(interaction)) (PMID: 23284291), [Crohn’s disease](http://www.ebi.ac.uk/gwas/search?query=Crohn) (PMID: 22412388), [nasopharyngeal carcinoma](http://www.ebi.ac.uk/gwas/search?query=Nasopharyngeal%20carcinoma) (PMID: 19664746) |
| *PPP1R18* | rs2269704 | C/T | None |
| *NRM* | rs2269703 | G/A | None |
| 6p21.3 | rs495089 | T/C | [Age-related macular degeneration](http://www.ebi.ac.uk/gwas/search?query=Age-related%20macular%20degeneration) (PMID: 22694956), [nevirapine-induced rash](http://www.ebi.ac.uk/gwas/search?query=Nevirapine-induced%20rash) (PMID: 21810746), [chronic lymphocytic leukemia](http://www.ebi.ac.uk/gwas/search?query=Chronic%20lymphocytic%20leukemia) (PMID: 21131588), [nasopharyngeal carcinoma](http://www.ebi.ac.uk/gwas/search?query=Nasopharyngeal%20carcinoma) (PMID: 19664746) |
| *MDC1* | rs2269702 | A/G | [IgG glycosylation](http://www.ebi.ac.uk/gwas/search?query=IgG%20glycosylation) (PMID: 23382691) |

Data were obtained from GWAS Catalog (http://www.ebi.ac.uk/gwas), and phenotypes related to serum LDL-cholesterol are shown in bold. PMID, PubMed ID; chr., chromosome.
