# Supplementary material for: Identification of eight genetic variants as novel determinants of dyslipidemia in Japanese by exome-wide association studies
Source: Oncotarget. 2017 Apr 17;8(24):38950–61. doi: 10.18632/oncotarget.17159 (PMC5503585; doi:10.18632/oncotarget.17159)
Supplement: Supplementary file 18 [file oncotarget-08-38950-s018.docx]

**Supplementary Table 19.** Minor allele frequencies and effect sizes of the 46 SNPs associated with serum concentrations of triglycerides in the present study.

| Gene (or chr. locus) | SNP | Nocleotide (amino acid) substitution | Minor allele frequency (%) | Differences in serum triglycerides among genotypes (%) |
| --- | --- | --- | --- | --- |
| Associated with serum triglycerides and hypertriglyceridemia | | | | |
| *BUD13* | rs10790162 | G/A | 26.3 | 19.6 |
| 11q23.3 | rs7350481 | C/T | 27.7 | 19.4 |
| Associated with serum triglycerides | | | | |
| *APOA5* | rs2075291  rs2266788 | C/A (G185C)  T/C | 7.3  26.2 | 45.2  20.2 |
| *ZPR1* | rs964184  rs2075290 | C/G  T/C | 26.3  26.7 | 20.2  18.7 |
| 11q23.3 | rs9326246  rs12269901  rs4938303 | G/C  G/C  T/C | 26.5  36.6  43.0 | 19.2  9.4  7.4 |
| *MTFR2* | rs143974258 | G/A (R360*) | 3.3 | 19.8 |
| *APOA4* | rs5104 | T/C (N147S) | 35.7 | 11.7 |
| *C21orf59* | rs76974938 | C/T (D67N) | 2.4 | 19.8 |
| *LPL* | rs328  rs15285  rs13702  rs326  rs301 | C/G (S474*)  G/A  A/G  A/G  T/C | 12.9  19.2  19.2  19.4  19.3 | 7.5  6.8  6.8  6.8  6.8 |
| 8p21.3 | rs10096633  rs17482753  rs12678919  rs10503669  rs7016880  rs2197089  rs1441756  rs2083637 | C/T  G/T  A/G  C/A  G/C  C/T  T/G  T/C | 12.7  12.6  12.6  12.6  12.0  27.6  19.0  19.0 | 7.5  7.5  6.8  6.8  6.2  7.4  6.2  6.2 |
| *SIK3* | rs2075292  rs10047462 | G/T  G/T | 42.7  47.6 | 11.4  10.7 |
| *GCKR* | rs1260326  rs780093 | T/C (L446P)  A/G | 43.6  43.0 | 11.3  11.3 |
| 2p23 | rs1260333 | T/C | 42.9 | 11.3 |
| *TNC* | rs138406927 | C/T (A1096T) | 2.1 | 18.4 |
| *LAIR2* | rs34429135 | T/A (F115Y) | 2.5 | 17.8 |
| *PAFAH1B2* | rs7112513  rs4936367 | G/A  A/G (V151M) | 45.3  45.3 | 9.3  9.3 |
| *C2orf16* | rs1919128  rs1919127 | G/A (V774I)  C/T (A685V) | 42.2  42.2 | 9.3  9.2 |
| *OR4F6* | rs141569282 | G/A (A117T) | 1.7 | 19.0 |
| 8q24.1 | rs2954038  rs2954033  rs2954026 | A/C  G/A  G/T | 30.3  33.3  33.1 | 9.7  8.6  8.6 |
| LOC101929011 | rs1240773 | G/T | 29.0 | 9.7 |
| *COL6A5* | rs200982668 | G/A (E2501K) | 1.3 | 18.8 |
| 6p21.3 | rs1264429 | A/G | 13.4 | 10.2 |
| *MUC17* | rs78010183 | A/T (T1305S) | 1.8 | 14.6 |
| *MARCH1* | rs61734696 | G/T (Q137K) | 1.2 | 18.1 |
| *MRVI1* | rs4909945 | C/T (V11I) | 0.3 | 28.1 |
