# Supplementary material for: Identification of eight genetic variants as novel determinants of dyslipidemia in Japanese by exome-wide association studies
Source: Oncotarget. 2017 Apr 17;8(24):38950–61. doi: 10.18632/oncotarget.17159 (PMC5503585; doi:10.18632/oncotarget.17159)
Supplement: Supplementary file 19 [file oncotarget-08-38950-s019.docx]

**Supplementary Table 20.** Minor allele frequencies and effect sizes of the 104 SNPs associated with serum concentrations of HDL-cholesterol in the present study.

| Gene (or chr. locus) | SNP | Nocleotide (amino acid) substitution | Minor allele frequency (%) | Differences in serum HDL-cholesterol among genotypes (%) |
| --- | --- | --- | --- | --- |
| Associated with serum HDL-cholesterol and hypo-HDL-cholesterolemia | | | | |
| *USP4* | rs146515657 | T/C (N650S) | 0.5 | 21.3 |
| 12q24.1 | rs12229654 | T/G | 22.5 | 4.0 |
| Associated with serum HDL-cholesterol | | | | |
| *LPGAT1* | rs150552771 | T/C (K200E) | 5.0 | 18.0 |
| *LAIR2* | rs34429135 | T/A (F115Y) | 2.5 | 21.7 |
| *KRR1* | rs17115182 | G/A (P43S) | 7.0 | 12.7 |
| *EHD3* | rs116417209 | G/A (V151I) | 3.5 | 18.7 |
| 16q13 | rs3764261  rs247616  rs173539 | G/T  C/T  C/T | 19.8  19.7  30.0 | 10.6  10.0  7.7 |
| 6p22.1 | rs9261800  rs9295895 | C/G  T/C | 2.8  27.6 | 18.0  3.9 |
| *DCLRE1C* | rs150854849 | C/T (R179Q) | 2.4 | 18.7 |
| *STYK1* | rs138533962 | G/A (R379C) | 2.0 | 19.3 |
| *CETP* | rs2303790  rs1532624  rs9939224  rs7499892  rs1800775 | A/G (D459G)  G/T  G/T  C/T  A/C | 3.6  29.6  9.5  17.2  45.0 | 25.8  7.1  9.4  6.0  5.3 |
| *APOA5* | rs2075291  rs2266788 | C/A (G185C)  T/C | 7.3  26.2 | 18.7  4.7 |
| *MUC17* | rs78010183 | A/T (T1305S) | 1.8 | 13.0 |
| *LIPC* | rs1800588  rs261334 | T/C  G/C | 49.9  46.1 | 8.4  6.5 |
| *OR4F6* | rs141569282 | G/A (A117T) | 1.7 | 13.8 |
| *CYP4F8* | rs201166643 | C/A (R488S) | 1.1 | 18.8 |
| *ACAD10* | rs11066015 | G/A | 27.5 | 7.3 |
| *ALDH2* | rs671 | G/A (E504K) | 27.6 | 7.3 |
| *CACNA1D* | rs35874056 | G/A (G460S) | 2.0 | 17.7 |
| *BRAP* | rs3782886 | A/G | 29.3 | 6.6 |
| *HECTD4* | rs2074356  rs11066280 | C/T  T/A | 25.4  29.0 | 7.3  6.6 |
| *LILRB2* | rs73055442 | C/T (R103H) | 1.6 | 16.0 |
| *COL6A5* | rs200982668 | G/A (E2501K) | 1.3 | 12.5 |
| *VPS33B* | rs199921354 | C/T (R80Q) | 1.2 | 13.0 |
| *MARCH1* | rs61734696 | G/T (Q137K) | 1.2 | 12.5 |
| *SLC9A3* | rs143027124 | C/T (V213I) | 1.1 | 18.0 |
| *MOB3C* | rs139537100 | C/T (R24Q) | 1.2 | 12.5 |
| *PRAMEF12* | rs199576535 | G/A (V341I) | 1.0 | 13.5 |
| *PLCB2* | rs200787930 | C/T (E1095K) | 1.2 | 12.5 |
| *CXCL8* | rs188378669 | G/T | 1.2 | 12.0 |
| *TMOD4* | rs115287176 | G/A (R277W) | 1.2 | 12.5 |
| *ADGRL3* | rs192210727 | G/T (R580I) | 1.3 | 11.4 |
| *ZNF77* | rs146879198 | G/A (R340*) | 1.2 | 12.0 |
| *COL6A3* | rs146092501 | C/T (E1386K) | 1.2 | 12.0 |
| *IQCF1* | rs200134435 | G/A (R103W) | 0.8 | 19.3 |
| *CYP4F12* | rs609636 | G/A (D76N) | 2.3 | 11.3 |
| *LPL* | rs15285  rs13702  rs326  rs301  rs328 | G/A  A/G  A/G  T/C  C/G (S474*) | 19.2  19.2  19.4  19.3  12.9 | 5.2  5.2  4.6  4.6  4.6 |
| 6p21.3 | rs7773955  rs2517518 | C/T  G/A | 26.3  28.3 | 4.0  3.9 |
| 8p21.3 | rs2197089  rs2083637  rs1441756  rs17482753  rs10096633  rs10503669  rs12678919  rs7016880 | C/T  T/C  T/G  G/T  C/T  C/A  A/G  G/C | 27.6  19.0  19.0  12.6  12.7  12.6  12.6  12.0 | 5.2  5.2  5.2  4.6  4.6  4.6  4.6  3.9 |
| LOC101928635 | rs1532085  rs10468017  rs2043085  rs4775041 | A/G  C/T  A/G  G/C | 42.1  20.3  40.9  20.2 | 5.3  5.2  4.6  5.2 |
| *NAA25* | rs12231744 | C/T (R876K) | 35.1 | 5.2 |
| *BUD13* | rs10790162 | G/A | 26.3 | 4.7 |
| *PTCH2* | rs147284320 | C/T (V503I) | 2.0 | 9.0 |
| *ZPR1* | rs964184  rs2075290 | C/G  T/C | 26.3  26.7 | 4.7  4.7 |
| *OR52I1* | rs200585398 | A/G (M167V) | 0.5 | 18.7 |
| *ABCA1* | rs1883025  rs2066714 | G/A  C/T (M883I) | 28.8  36.0 | 5.3  4.0 |
| *ATXN2* | rs7969300 | T/C (N248S) | 38.8 | 5.2 |
| 11q23.3 | rs9326246  rs7350481 | G/C  C/T | 26.5  27.7 | 4.7  4.0 |
| *TCF19* | rs61733202 | G/A (G26R) | 0.2 | 29.3 |
| *OAS3* | rs2072134 | C/T | 17.6 | 4.7 |
| *LOC554223* | rs1610640 | A/G | 41.6 | 7.7 |
| *HLA-B* | rs1058026 | T/G | 33.4 | 4.6 |
| *PLCD1* | rs147186786 | C/T (R268Q) | 0.2 | 17.3 |
| *DENND1C* | rs200449136 | G/A | 0.3 | 21.5 |
| *LOC101929163* | rs3129945 | G/A | 33.6 | 3.3 |
| *ANKRD11* | rs139088883 | G/A (A1840V) | 0.3 | 19.3 |
| *BTNL2* | rs2076528  rs3763315  rs41441651  rs28362675  rs41417449  rs78587369  rs34423804  rs3806156 | T/G  G/T  C/T (D336N)  C/A (E454*)  T/C (M295V)  G/A (T165I)  T/A (V283D)  G/T | 23.0  23.0  23.0  23.0  23.0  23.0  23.0  49.1 | 5.8  5.8  5.8  5.8  5.8  5.8  5.8  4.0 |
| *APOE* | rs7412 | C/T (R176C) | 4.3 | 5.2 |
| *CD36* | rs75326924 | C/T (P90S) | 4.5 | 7.5 |
| *NOS3* | rs7792133 | G/A (R665H) | 0.3 | 19.9 |
| *ACE* | rs4314 | C/T (R561W) | 0.3 | 20.5 |
| *TICRR* | rs150565858 | G/A (R301Q) | 0.2 | 22.1 |
| *HCG22* | rs3873352  rs2523849 | G/C  A/G | 27.1  28.3 | 3.9  3.9 |
| *SKIV2L* | rs592229 | G/T | 42.4 | 4.0 |
| *ZNF33B* | rs7914982 | T/C (H244R) | 0.3 | 19.3 |
| *PPP1R10* | rs3895681 | G/C | 3.3 | 5.4 |
| *CAT* | rs139421991 | G/A (R320Q) | 0.3 | 16.2 |
| *TNC* | rs138406927 | C/T (A1096T) | 2.1 | 6.3 |
