# Supplementary material for: Identification of eight genetic variants as novel determinants of dyslipidemia in Japanese by exome-wide association studies
Source: Oncotarget. 2017 Apr 17;8(24):38950–61. doi: 10.18632/oncotarget.17159 (PMC5503585; doi:10.18632/oncotarget.17159)
Supplement: Supplementary file 20 [file oncotarget-08-38950-s020.docx]

**Supplementary Table 21.** Minor allele frequencies and effect sizes of the 40 SNPs associated with serum concentrations of LDL-cholesterol in the present study.

| Gene (or chr. locus) | SNP | Nocleotide (amino acid) substitution | Minor allele frequency (%) | Differences in serum LDL-cholesterol among genotypes (%) |
| --- | --- | --- | --- | --- |
| Associated with serum LDL-cholesterol and hyper-LDL-cholesterolemia | | | | |
| 6p21.3 | rs2853969 | C/T | 9.7 | 6.1 |
| Associated with serum LDL-cholesterol | | | | |
| *APOE* | rs7412  rs769449 | C/T (R176C)  G/A | 4.3  7.7 | 16.8  4.6 |
| *APOC1* | rs445925 | C/T | 6.6 | 4.1 |
| *APOB* | rs13306206  rs13306194 | G/A (P955S)  G/A (R532W) | 3.2  12.1 | 24.8  5.4 |
| *PCSK9* | rs151193009 | C/T (R93C) | 1.1 | 24.6 |
| *PSRC1* | rs599839 | A/G | 7.9 | 8.3 |
| *CELSR2* | rs629301  rs12740374  rs646776 | A/C  G/T  A/G | 7.8  7.7  7.7 | 8.9  8.6  8.0 |
| 1p13.3 | rs602633 | C/A | 7.6 | 9.2 |
| *ABO* | rs1053878 | G/A (P156L) | 22.8 | 3.8 |
| 9q34.2 | rs651007  rs579459  rs635634  rs507666 | G/A  T/C  G/A  G/A | 27.9  27.9  27.8  27.8 | 3.2  3.2  3.2  2.8 |
| *MUC22* | rs117024916 | A/G (T71A) | 9.9 | 5.5 |
| *VARS* | rs11751198  rs5030798 | G/A  C/T (V1055I) | 9.5  9.5 | 6.3  6.3 |
| *CCHCR1* | rs147733073 | C/G (H486Q) | 10.2 | 6.1 |
| 6p21.3 | rs12210887  rs2596574 | G/T  G/A | 9.7  9.7 | 6.3  5.8 |
| *MSH5* | rs11754464 | C/T | 9.5 | 6.3 |
| *PRRC2A* | rs11538264 | G/A (V1774M) | 9.5 | 6.3 |
| *FAM65B* | rs150142878 | C/T (R371Q) | 5.6 | 4.6 |
| *HSPA1B* | rs6457452 | C/T | 9.7 | 6.3 |
| *LY6G6C* | rs117894946 | G/C (G75A) | 9.5 | 6.3 |
| *C6orf48* | rs11968400 | C/T | 9.7 | 6.3 |
| *KIAA0319* | rs4576240 | G/T (P142T) | 5.5 | 3.7 |
| *ZSCAN31* | rs6922302 | C/G (P128A) | 9.6 | 4.9 |
| *NEU1* | rs13118 | T/A | 9.7 | 6.1 |
| *ZSCAN26* | rs76463649 | A/G (N15S) | 9.6 | 5.5 |
| *LY6G6F* | rs17200983  rs9267546  rs9267547 | C/A (P34Q)  G/A  G/A (A107T) | 9.5  9.8  10.0 | 6.3  6.3  6.9 |
| 6p22.1 | rs3129029 | A/C | 23.0 | 4.9 |
| *TNXB* | rs140770834  rs11751545 | C/G (L2271V)  A/C | 8.8  8.8 | 6.3  6.3 |
| *ABCF1* | rs4148249 | C/A | 10.1 | 4.6 |
